# Predicting RNA-binding sites of proteins using support vector machines and evolutionary information

**DOI:** 10.1186/1471-2105-9-S12-S6

**Published:** 2008-12-12

**Authors:** Cheng-Wei Cheng, Emily Chia-Yu Su, Jenn-Kang Hwang, Ting-Yi Sung, Wen-Lian Hsu

**Affiliations:** 1 Institute of Information Systems and Applications, National Tsing Hua University, Hsinchu, Taiwan; 2 Institute of Bioinformatics, National Chiao Tung University, Hsinchu, Taiwan; 3 Bioinformatics Program, Taiwan International Graduate Program, Academia Sinica, Taipei, Taiwan; 4 Bioinformatics Lab., Institute of Information Science, Academia Sinica, Taipei, Taiwan

## Abstract

**Background:**

RNA-protein interaction plays an essential role in several biological processes, such as protein synthesis, gene expression, posttranscriptional regulation and viral infectivity. Identification of RNA-binding sites in proteins provides valuable insights for biologists. However, experimental determination of RNA-protein interaction remains time-consuming and labor-intensive. Thus, computational approaches for prediction of RNA-binding sites in proteins have become highly desirable. Extensive studies of RNA-binding site prediction have led to the development of several methods. However, they could yield low sensitivities in trade-off for high specificities.

**Results:**

We propose a method, RNAProB, which incorporates a new smoothed position-specific scoring matrix (PSSM) encoding scheme with a support vector machine model to predict RNA-binding sites in proteins. Besides the incorporation of evolutionary information from standard PSSM profiles, the proposed smoothed PSSM encoding scheme also considers the correlation and dependency from the neighboring residues for each amino acid in a protein. Experimental results show that smoothed PSSM encoding significantly enhances the prediction performance, especially for sensitivity. Using five-fold cross-validation, our method performs better than the state-of-the-art systems by 4.90%~6.83%, 0.88%~5.33%, and 0.10~0.23 in terms of overall accuracy, specificity, and Matthew's correlation coefficient, respectively. Most notably, compared to other approaches, RNAProB significantly improves sensitivity by 7.0%~26.9% over the benchmark data sets. To prevent data over fitting, a three-way data split procedure is incorporated to estimate the prediction performance. Moreover, physicochemical properties and amino acid preferences of RNA-binding proteins are examined and analyzed.

**Conclusion:**

Our results demonstrate that smoothed PSSM encoding scheme significantly enhances the performance of RNA-binding site prediction in proteins. This also supports our assumption that smoothed PSSM encoding can better resolve the ambiguity of discriminating between interacting and non-interacting residues by modelling the dependency from surrounding residues. The proposed method can be used in other research areas, such as DNA-binding site prediction, protein-protein interaction, and prediction of posttranslational modification sites.

## Background

RNA-protein interaction plays an important role in various biological processes, such as protein synthesis, gene expression, posttranscriptional regulation, and viral infectivity. The prediction results of RNA-binding sites in proteins can provide biological insights for investigating RNA-protein interaction. For instance, the ribosome is a protein synthesis complex consisting of ribosomal RNAs (rRNAs) and proteins. Sunita *et al. *[1] applied predicted RNA-binding sites to study the relationship between RNA methyltransferases RsmC and 16S rRNA. In addition, Bechara *et al. *[2] incorporated predicted results from a RNA-binding site predictor to inspect the connection between fragile X mental retardation protein and G-quartet RNA structure. Moreover, some RNA viruses, such as human immunodeficiency virus (HIV) and hepatitis C virus, have a RNA genome and replicate themselves by interacting with host proteins [3]. Therefore, identification of the RNA interacting residues in proteins provides valuable information for understanding the mechanisms of protein synthesis, gene regulation, and pathogen-host interaction.

In recent years, rapid advances in genomic and proteomic studies have yielded a tremendous amount of DNA and protein sequences. We used the keyword "RNA-binding" to search against the National Center for Biotechnology Information (NCBI) protein sequence database on June 9, 2008, and obtained 196,686 protein sequences. However, when searching against Protein Data Bank (PDB) [4] for molecular/chain type containing protein and RNA, we only retrieved 684 structures. In addition, experimental determination of RNA-protein interaction remains time-consuming and labor-intensive. Therefore, computational approaches for predicting RNA-binding sites in proteins have become increasingly important to understand the mechanisms of RNA-protein interaction.

### Previous work

Extensive studies of RNA-protein binding site prediction have lead to the development of several methods, which can be classified as follows.

#### 1. Amino acid composition-based methods

Jeong *et al. *[5] used an artificial neural network (ANN) to predict RNA-protein interacting residues based on amino acid compositions and predicted secondary structure elements. It achieved Matthew's correlation coefficient (MCC) of 0.29 and overall accuracy of 77.50% along with specificity of 87.29% and sensitivity of 40.30%. Terribilini *et al. *[6] presented RNABindR using a Naïve Bayes classifier on amino acid sequences to predict RNA binding sites in proteins. RNABindR attained MCC, overall accuracy, specificity, and sensitivity of 0.35, 84.80%, 93% and 38%, respectively.

#### 2. Evolutionary information-based methods

Jeong and Miyano [7] applied an ANN to predict the RNA interacting residues based on evolutionary information from the position-specific scoring matrix (PSSM), and achieved MCC, overall accuracy, specificity, and sensitivity of 0.39, 80.20%, 91.04%, and 43.40%, respectively. The MCC is further improved to 0.41 by the incorporation of weighted profiles. Kumar *et al. *proposed a predictor, PPRint [8], using PSSM profiles in a support vector machine (SVM) model, and it achieved MCC, overall accuracy, specificity, and sensitivity of 0.45, 81.16%, 89.55%, and 53.05%, respectively.

#### 3. Hybrid methods

Wang and Brown [9] developed an SVM-based classifier, BindN, using features including relative solvent accessible surface area, hydrophobicity index, side chain pKa value, molecular mass, and BLAST results. The overall accuracy, specificity, and sensitivity of BindN are 74.25%, 75.70%, and 65.78%, respectively.

### Challenges

Although many methods have been proposed for RNA-binding site prediction, several challenges still remain. First, many of previous methods yield low sensitivities in tradeoff for high specificities since some biological applications, such as identification of critical residues for site-specific mutagenesis, emphasize more on specificities rather than sensitivities [6,8]. These methods could suffer from low coverage of RNA-binding sites in high-throughput proteomic analyses. Second, the MCC values of existing methods remain in the range of 0.27~0.45, which presents a great scope for improvement in the complementary measure of prediction performance. Finally, in most methods parameters such as the size of the sliding window are selected from test results evaluated by *n*-fold cross-validation, which may lead to overestimation of the prediction performance. Thus, the performance would be worse if a more rigorous procedure is applied for parameter selection and performance evaluation.

### Our method and future applications

In this study, we propose a method, RNAProB (RNA-Protein Binding site prediction), for prediction of RNA-binding residues in proteins using SVM classifiers and a new smoothed PSSM encoding scheme. Besides incorporation of upstream and downstream residues in a standard PSSM generated by PSI-BLAST, smoothed PSSM encoding also considers, for each amino acid in a sequence, the dependency effect from its neighboring amino acids. Similar to the spatial domain method used in the research field of image processing [10], smoothed PSSM encoding calculates the evolutionary information of a central position based on the sum of those from surrounding residues. Experimental results show that the prediction performance of smoothed PSSM encoding performs better than the state-of-the-art approaches on the benchmark data sets. Evaluated by five-fold cross-validation, RNAProB outperforms the other approaches by 0.10~0.23 in MCC, 4.90%~6.83% in overall accuracy, and 0.88%~5.33% in specificity. Most notably, our method significantly improves sensitivity by 26.90%, 26.62%, and 7.05% for the RBP86, RBP109, and RBP107 data sets, respectively. To avoid data overfitting, we also incorporate a three-way data split procedure to evaluate the prediction performance of RNAProB. Our results show that our method not only achieves significant improvement on the performance, but also attains a high prediction accuracy evaluated by a three-way data split procedure. Moreover, our analysis indicates that smoothed PSSM could serve as a more discriminative feature for distinguishing between interacting and non-interacting residues. We believe that the proposed encoding scheme could be applicable to other research fields, such as DNA-binding sites, protein-protein interaction, and prediction of posttranslational modification sites.

## Methods

### Data sets

In this study, we apply three data sets used in previous studies to compare the performance of our method and other systems. Table 1 shows a summary of these data sets, which are detailed as follows and available in the supplementary material [see Additional files 1, 2, and 3].

**Table 1:** Summary of three benchmark data sets

**Data set**	**RBP86**	**RBP109**	**RBP107**
Number of protein chains	86	109	107
X-ray crystallography resolution	>3 Å	>3.5 Å	>3.5 Å
Sequence identity	≤70%	≤30%	≤25%
Number of interacting residues	4,568	3,581	2,555
Number of non-interacting residues	15,503	21,526	19,496
Non-interacting/interacting residues	3.39	6.01	7.63
Total number of residues	20,071	25,107	22,051

#### 1. RBP86

The RBP86 data set consists of 86 protein chains extracted from RNA-protein complexes with X-ray crystallography resolution better than 3 Å in PDB. Sequence redundancy in the data set is removed so that no protein pair has a sequence identity greater than 70%. In the RNA-protein complexes, a residue is regarded as interacting with RNA if the distance between an RNA molecule and the residue in the protein is less than 6 Å. The resultant data set contains 4,568 RNA interacting residues and 15,503 non-interacting residues. The RBP86 data set has been used in Terribilini *et al. *[6] and Kumar *et al. *[8]. In Kumar *et al.*, it is also referred to as the "main" data set.

#### 2. RBP109

The RBP109 data set contains 109 protein sequences obtained from 56 RNA-protein complexes with X-ray crystallography resolution better than 3.5 Å in PDB. For any two protein chains, the sequence identity is no more than 30%. The numbers of interacting and non-interacting residues are 3,581 and 21,526, respectively. The RBP109 data set is downloaded from RNABindR web server [11]. In Terribilini *et al. *[6], this is named as the "RB109" data set.

#### 3. RBP107

Derived from 61 RNA-protein complexes in PDB, the RBP107 data set is comprised of 107 protein chains with X-ray crystallography resolution better than 3.5 Å and sequence identity no more than 25%. Based on the cut-off distance of 3.5 Å, the RBP107 data set contains 2,555 interacting residues and 19,496 non-interacting ones. Wang and Brown [9] applied this data set to construct and evaluated their approach. In Kumar *et al. *[8], it is referred to as the "alternate" data set.

### Support vector machines (SVM)

SVM is a machine learning approach proposed by Vapnik [12] based on structural risk minimization principle of statistics learning theory. It can be used to deal with classification or regression. Distinguishing RNA binding residues form non-binding residues in a protein could be regarded as a binary classification problem. For a set of given input data vectors *x*_*i *_(x_i _∈ ℝ^d^, i = 1, 2,..., n) with labels *y*_*i *_(y_i _∈ {+1, -1}, i = 1, 2,..., n; where "+1" represents a positive instance and "-1" denotes a negative instance), the mission in the training procedure is to optimize the following equation that maps input vectors into a higher dimensional feature space (i.e., Hilbert space), and seeks a separation hyperplane with a maximum margin to divide positive instances from negative ones. The calculation of SVM is defined in Equation (1).

(1) $$\begin{array}{c} {\text{Min}}_{\text{w},\text{b},\xi_{\text{i}}}\left(\frac{1}{2}{\text{w}}^{\text{T}}\text{w}+\text{C}\sum_{\text{i}=1}^{\text{n}}\xi_{\text{i}}\right)\\ {\text{subject to y}}_{\text{i}}({\text{w}}^{\text{T}}\Phi ({\text{x}}_{\text{i}})+\text{b})\geq 1-\xi_{\text{i}},\xi_{\text{i}}\geq 0,\text{i}=1,2,...,\text{n}, \end{array}$$

where w ∈ ℝ^d ^is a weight vector, *b *is a bias (constant), and Φ is a mapping function. For more flexible classification, SVM allows instance *i *positions at the wrong side of hyperplane with slack variable *ξ*_*i *_and cost parameter *C*. In SVM, a kernel function K(x_i_, x_j_), such as linear, polynomial, radial basis function (RBF), and sigmoid function, is used to present Φ(x_i_)·Φ(x_j_) where *x*_*i *_and *x*_*j *_are two data vectors. In this study, we use RBF as the kernel function in the SVM. The formulation of RBF is defined in Equation (2), where *γ *is a training parameter.

(2) K(x_i_, x_j_) = exp(-*γ*||x_i _- x_j_||^2^)

Developed by Lin *et al. *[13], LIBSVM is a powerful and well-known SVM package used by many researchers. We apply LIBSVM to implement our classifiers for prediction of RNA-binding sites in proteins.

### Feature extraction and representation

Evolutionary information has been shown to be effective for RNA-binding site prediction [8]. For this reason, we use PSI-BLAST [14] to search against NCBI non-redundant (nr) database and generate a PSSM based on BLOSUM62 substitution matrix [15] for each protein with e-value as 0.001 and iteration number as 3. A PSSM is comprised of *L *vectors (*L *denotes the length of the protein), in which contain the log-likelihoods for different amino acids in a position. Next, we illustrate two different encoding schemes to represent the PSSM.

#### 1. Standard PSSM encoding scheme

**Figure 1:**
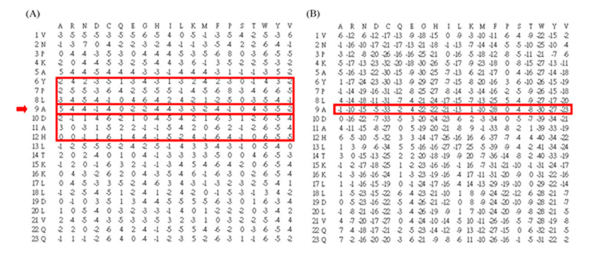
Examples of (A) standard PSSM and (B) smoothed PSSM generated by PSI-BLAST (e-value = 0.001, iteration number = 3).

Standard PSSM has been used for RNA-binding site prediction by Kumar *et al. *[8]. For a PSSM profile, the feature representation of a residue *α*_*i *_at position *i *in a protein sequence is presented by an evolutionary information vector *V*_*i *_comprised of log-likelihoods for 20 different amino acids. Considering the surrounding residues of *α*_*i*_, we apply a sliding window of size *w *to incorporate the evolutionary information from upstream and downstream neighbors. The feature vector of a residue *α*_i _is represented by (V_i-(w-1)/2_,..., V_i_,..., V_i+(w-1)/2_). For the N-terminal and C-terminal of a protein, (w-1)/2 ZERO vectors, consisting of 20 zero elements, are appended to the hand or tail of a PSSM profile. The feature values in each vector are normalized to a range between -1 and 1. In our study, we apply different sliding window sizes ranging from 3 to 41 with a step as 2 (i.e., *w *= 3, 5,..., 41). Figure 1(A) shows an example of standard PSSM of a protein with e-value as 0.001 and iteration number as 3 in PSI-BLAST.

#### 2. Smoothed PSSM encoding scheme

In addition to the consideration of neighbors of a residue *α*_*i*_, we propose a new encoding scheme to incorporate the dependency of surrounding residues. In a standard PSSM profile, the log-likelihood at each position is calculated based on an assumption that each position is independent from the others. However, Terribilini *et al. *[6] observed that RNA binding residues tend to occur in clusters. Their analysis revealed that 95% of interacting residues in the RBP109 data set have at least one additional interacting residue among the four amino acids on either side, and 49% of those have at least four. Inspired by the consideration of adjacent pixels used in the spatial domain method from the research field of image processing [10], we present a new encoding scheme to model the dependency or correlation among surrounding neighbors of a central residue. Similar to the feature representation in standard PSSM encoding, we use a sliding window of size *w *to incorporate the evolutionary information from upstream and downstream residues. In the construction of a smoothed PSSM, each row vector of a residue *α*_*i *_is represented and smoothed by the summation of *ws *surrounding row vectors (V_smoothed_i _= V_i-(ws-1)/2 _+ ... + V_i _+ ... + V_i+(ws-1)/2_). For the N-terminal and C-terminal of a protein, (w-1)/2 ZERO vectors, are appended to the hand or tail of a smoothed PSSM profile. Using the smoothed PSSM encoding scheme, the feature vector of a residue *α*_*i *_is represented by (V_smoothed_i-(w-1)/2_,..., V_smoothed_i_,..., V_smoothed_i+(w-1)/2_). The feature values in each vector are normalized to a range between -1 and 1. Here, we apply different smoothing window sizes from 3 to 11 with a step as 2 (i.e., *ws *= 3, 5,..., 11). Figure 1(B) illustrates an example of a smoothed PSSM profile. At position 9, the corresponding value of amino acid 'A' represented by a smoothed PSSM encoding is the sum of [(-2)+(-2)+(-3)+5+(-2)+3+0].

### Window size selection and parameter optimization

In order to optimize the performance of RNAProB, we have to determine the best combination of several parameters, including the sliding window size *w*, cost parameter *C *and kernel parameter *γ *in the SVM classifier, the smoothing window size *ws*, and the weight parameters *w*_1 _and *w*_-1 _in SVM. Table 2 shows the workflow of window size selection and parameter optimization. In our study, the best parameters are optimized with respect to overall accuracy. First, we test the performance of different sliding window sizes *w *from 3, 5, 7,..., 41 in standard PSSM encoding scheme using default *C *and *γ *parameters in SVM, and initial weight parameter *w*_1 _as 1 and *w*_-1 _as the ratio of the number of non-interacting residues to that of interacting residues in a data set. As shown in Table 1, the ratios of the numbers of non-interacting residues to those of interacting residues in the RBP86, RBP109, and RBP107 data sets are 1:3.39, 1:6.01, and 1:7.63, respectively. Second, based on the optimized sliding window size *w *selected from the first step, the best combination of cost parameter *C *and kernel parameter *γ *is determined with initial weight parameters. The log_2_C and log_2_*γ *ranged from -3 to 12 and -3 to -15, respectively. Third, the prediction performance of different smoothing window sizes *ws *ranged from 3 to 11 with a step 2 is evaluated using initial weight parameters and previously selected parameters (i.e., *w*, *C*, and *γ*). Fourth, due to data set imbalance, the weight parameters *w*_1 _and *w*_-1 _are tuned with optimized *w*, *C*, *γ*, and *ws*. After these steps, the optimal parameters, including sliding window size *w*, cost parameter *C*, kernel parameter *γ*, smoothing window size *ws*, and weight parameters *w*_1 _and *w*_-1_, are determined.

**Table 2:** The workflow of window size selection and parameter optimization.

	**Sliding window size (*w*)**	***C *and *γ***	**Smoothing window size (*ws*)**	**Weight parameter (*w*_1 _and *w*_-1_)**
Step 1	3 ≤ *w *≤ 41 (step = 2)	Default	-	Default ratio

Step 2	Optimized *w *from step 1	-3 ≤ log_2_C ≤ 12 (step = 1)		Default ratio
		-3 ≤ log_2_*γ *≤ -15 (step = -1)		

Step 3	Optimized *w *from step 1	Optimized *C *and *γ *from step 2	3 ≤ *ws *≤ 11 (step = 2)	Default ratio

Step 4	Optimized *w *from step 1	Optimized *C *and *γ *from step 2	Optimized *ws *from step 3	1 ≤ *w*_1 _≤ 8^# ^(step = 1), *w*_-1 _= 1

Final	Optimized *w *from step 1	Optimized *C *and *γ *from step 2	Optimized *ws *from step 3	Optimized *w*_1 _and *w*_-1 _from step4

^# ^In the RBP107 data set, we test *w*_1 _ranged from 1 to 10 (step = 1).

### System architecture

**Figure 2:**
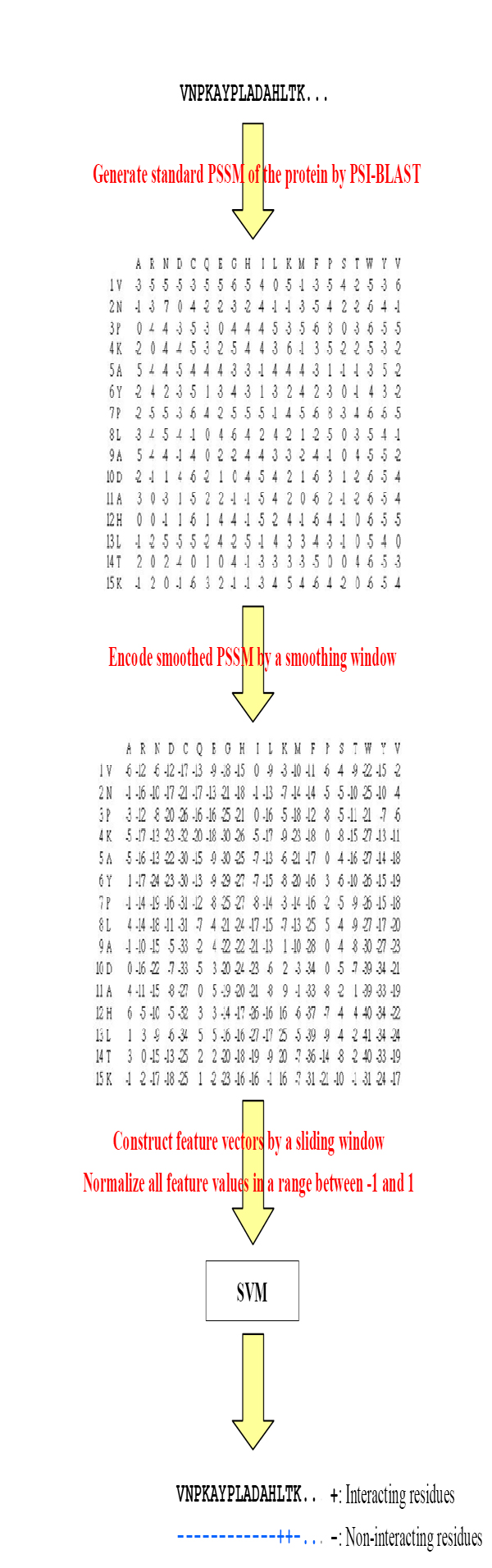
System architecture of RNAProB.

The system architecture of RNAProB is shown in Figure 2. Given a protein sequence, RNAProB performs the following steps:

1. Apply PSI-BLAST to generate a standard PSSM of the protein.

2. Generate a smoothed PSSM of the protein using an optimized smoothing window size.

3. Construct a feature vector for each residue in the protein sequence by an optimized sliding window size, and normalize all feature values in the vector into a range of -1 and 1.

4. Use a trained SVM classifier with optimized parameters (*C*, *γ*, *w*_1_, *w*_-1_) to predict the interacting and non-interacting residues in the protein.

After the above steps, RNAProB outputs the corresponding interacting or non-interacting state of each residue in the protein.

### Training and testing

The performance of RNAProB is assessed by *n*-fold cross-validation and three-way data split. To compare with other approaches, we use five-fold cross-validation to evaluate the performance of RNAProB. However, to prevent data-overfitting, a three-way data split procedure is applied to assess our predictor. The performance of RNAProB is evaluated as follows.

#### 1. *n*-fold cross-validation

A data set is randomly divided into five distinct non-overlapping sets of positive and negative instances (i.e., *n *= 5), four of which are used to train the predictor and the accuracy of the predictor is evaluated on the remaining set. This procedure is repeated five times.

#### 2. Three-way data split

To avoid over fitting, we use a more stringent three-way data split procedure [16,17] to evaluate the performance of RNAProB. A data set is randomly partitioned into three non-overlapping sets: a training set for classifier learning, a validation set for parameter selection, and a test set for performance evaluation. In this paper, we divide a data set into five distinct sets, three for training, one for validation, and one for testing. The procedure is also iterated 5 times.

### Performance evaluation measures

For comparison with other approaches, we follow the measures used in previous work [8,9,18], including specificity (Spec), sensitivity (Sens), MCC [19], and overall accuracy (Acc). Specificity and sensitivity measure how well the binary classifier recognizes negative and positive cases, respectively. A specificity of 100% and a sensitivity of 100% imply that the classifier identifies all non-interacting residues as non-interacting and all interacting residues as interacting, correspondingly. When a predictor's specificity increases, its sensitivity often decreases. On the other hand, MCC, which considers both under- and over-predictions, gives a complementary measure of the prediction performance, where MCC = 1 denotes a perfect prediction, MCC = 0 indicates a completely random assignment, and MCC = -1 means a perfectly reverse correlation. Moreover, overall accuracy presents how well the classifier distinguishes true positives and true negatives, and 100% overall accuracy denotes a perfect prediction. The definitions of specificity, sensitivity, MCC, and overall accuracy are defined in Equations (3), (4), (5), and (6), respectively. In the equations, *TP*, *TN*, *FP*, and *FN *denote the numbers of true positives, true negatives, false positives, and false negatives, correspondingly.

(3) Specificity = TN/(TN + FP) × 100

(4) Sensitivity = TP/(TP + FN) × 100

(5) $$\text{MCC}=(\text{TP}\times \text{TN}-\text{FP}\times \text{FN})/\sqrt{\left(\text{TP}+\text{FP})\times (\text{TP}+\text{FN})\times (\text{TN}+\text{FP})\times (\text{TN}+\text{FN}\right)}$$

(6) Acc = (TP + TN)/(TP + TN + FP + FN) × 100

In addition to the above measures, we also use the receiver operating characteristic (ROC) curve [20] and area under the ROC curve (AUC) [21] to evaluate the performance of standard and smoothed PSSM encoding schemes. In an ROC curve plot, the X-axis represents false positive rate (i.e., 1-specificity) and Y-axis denotes true positive rate (i.e., sensitivity). We incorporate different thresholds in the SVM classifier to plot the true positive rates against false positive rates in an ROC curve. Moreover, AUC calculates the area under an ROC curve and the maximum value of AUC is 1, which denotes a perfect prediction. A random guess results in an AUC value close to 0.5.

To determine the thresholds in the SVM classifiers, we follow the criteria used in the previous work. We notice that the thresholds in other approaches are optimized with respect to different measures. For example, Kumar *et al. *[8] and Jeong and Miyano [7] both optimized their results in the RBP86 data set based on MCC. In addition, Terribilini *et al. *[6] also selected the thresholds with the best MCC for the RBP109 data set. On the other hand, Wang and Brown [18] determined the best thresholds in the RBP107 data set based on the average of specificity and sensitivity. Therefore, the thresholds in RNAProB are optimized with respect to MCC for the RBP86 and RBP109 data sets, while the threshold is determined by the average of sensitivity and specificity for the RBP107 data set.

## Results

### Effect of smoothed PSSM encoding scheme

Here we compare the performance of smoothed PSSM and standard PSSM encoding scheme in terms of MCC, overall accuracy, ROC curve, and AUC for the benchmark data sets. Table 3 shows the performance comparison of standard PSSM and smoothed PSSM using five-fold cross-validation and three-way data split. Evaluated by five-fold cross-validation, smoothed PSSM encoding scheme attains overall accuracy of 87.99%, 89.70%, and 80.44% compared to 83.39%, 87.38%, and 77.80% by standard PSSM encoding for the RBP86, RBP109, and RBP107 data sets, respectively. Moreover, smoothed PSSM encoding scheme achieves improvements of 0.06~0.178 in MCC compared to standard PSSM. Similarly, assessed by three-way data split, smoothed PSSM encoding also performs better than standard PSSM in terms of both overall accuracy and MCC in the three data sets.

**Table 3:** Performance comparison of standard PSSM and smoothed PSSM.

**Data set**	**Smoothed PSSM**		**Standard PSSM**	
		
	Acc (%)	MCC	Acc (%)	MCC
RBP86	87.99 (87.65)	0.68 (0.67)	83.39 (83.35)	0.502 (0.496)
RBP109	89.70 (89.36)	0.58 (0.56)	87.38 (86.95)	0.45 (0.43)
RBP107	80.44 (79.84)	0.42 (0.40)	77.80 (77.55)	0.36 (0.35)

^§ ^The performance of incorporating a three-way data split procedure is shown in the parentheses.

**Figure 3:**
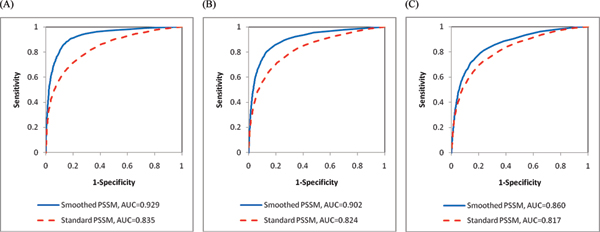
ROC curves and AUC of the (A) RBP86, (B) RBP109, and (C) RBP107 data sets.

Figure 3(A), (B), and 3(C) illustrate the ROC curves and AUC of smoothed PSSM and standard PSSM encoding schemes for the three benchmark data sets. The solid blue line and dotted red line represent the ROC curves plotted according to the performance of smoothed PSSM and standard PSSM encoding schemes, respectively. When smoothed PSSM encoding scheme is used to represent the proteins, AUC achieve 0.929, 0.902, and 0.860 on the RBP86, RBP109, and RBP107 data sets, respectively; on the other hand, standard PSSM only attains AUC of 0.835, 0.824, and 0.817.

Experimental results demonstrate that our proposed smoothed PSSM encoding scheme not only achieves good prediction performance, but also yields a significant improvement over standard PSSM encoding. Smoothed PSSM encoding scheme outperforms standard PSSM by 2.32%~4.60% in overall accuracy and 0.06~0.178 in MCC. The consideration of dependency among neighboring residues works well in distinguishing interacting residues from non-interacting ones; accordingly, the prediction performance of smoothed PSSM encoding scheme is substantially improved. This supports our assumption that the incorporation of the correlation between surrounding residues in PSSM profiles can significantly enhance the performance of RNA-binding site prediction.

### RNAProB prediction performance on the benchmark data sets

For each data set, we used five-fold cross-validation and three-way data split to evaluate the prediction performance, which is detailed below and summarized in Table 4.

**Table 4:** Performance of five-fold cross-validation and three-way data split for the benchmark data sets.

**Data set**	**Measurements**	**Spec. (%)**	**Sens. (%)**	**Acc (%)**	**MCC**	**Threshold**
RBP86	5-fold CV	90.36	79.95	87.99	0.68	0.36
	3-way data split	90.01	79.64	87.65	0.67	0.36

RBP109	5-fold CV	93.88	64.62	89.70	0.58	0.35
	3-way data split	94.14	60.63	89.36	0.56	0.35

RBP107	5-fold CV	80.87	77.14	80.44	0.42	0.11
	3-way data split	80.65	73.62	79.84	0.40	0.12

#### 1. Performance comparison with other approaches on the RBP86 data set

**Figure 4:**
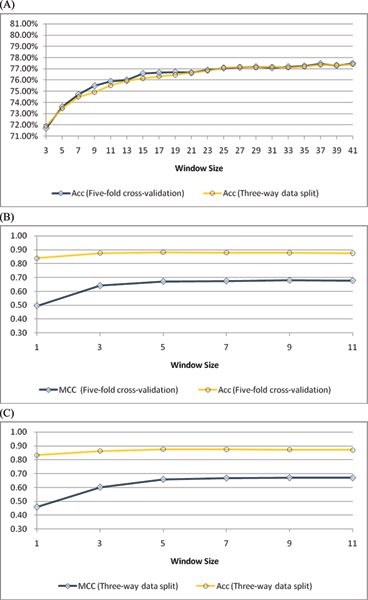
(A) Accuracy with respect to different sliding window sizes using five-fold cross-validation and three-way data split for the RBP86 data set, respectively. (B) The performance of the RBP86 data set with different smoothing window sizes by five-fold cross-validation. (C) The performance of the RBP86 data set with different smoothing window sizes by three-way data split.

The window sizes, including the sliding window size *w *and smoothing window size *ws*, and other parameters in RNAProB are selected with respect to overall accuracy. First, Figure 4(A) shows the overall accuracy of applying different sliding window sizes on the RBP86 data set. The overall accuracy evaluated by both five-fold cross-validation and three-way data split grows rapidly before it reaches 77%. However, a slow growth in the overall accuracy is observed as the size of sliding window is greater than 25. Thus, the sliding window size *w *is set as 25 for the RBP86 data set. Next the prediction performance of different smoothing window sizes based on previously determined sliding window size (i.e. *w *= 25) is illustrated in Figure 4(B) and 4(C). In Figure 4(B), although there is a very slow growth in the overall accuracy, we observe that MCC is improved from 0.50 to 0.67 when the size of smoothing window is increased from 1 to 7. Nevertheless, the performance improvement in MCC (i.e. improvement < 0.01) is not significant as the size of smoothing window is greater than 7. Similar trends in MCC and overall accuracy are also observed in Figure 4(C). Therefore, we use 7 as the smoothing window size *ws *in our method. As shown in Table 4, the performance of RNAProB evaluated by five-fold cross-validation achieves MCC, overall accuracy, specificity, and sensitivity of 0.68, 87.99%, 90.36%, and 79.95%, (with sliding window size *w *= 25, smoothing window size *ws *= 7, cost parameter *C *= 4, kernel function parameter *γ *= 0.015625, weight parameter *w*_1 _= 4, *w*_-1 _= 1, and threshold value = 0.36), respectively. Besides, using a more rigorous three-way data split procedure, our method also attains MCC, overall accuracy, specificity, and sensitivity of 0.67, 87.65%, 90.01%, and 79.64%, (with *w *= 25, *ws *= 7, *C *= 1, *γ *= 0.03125, *w*_1 _= 4, *w*_-1 _= 1, and threshold value = 0.36), correspondingly. The experimental results of window size selection and parameter optimization on the RBP86 data set are shown in the supplementary material [see Additional file 4].

The performance comparison with two other approaches developed on the same data set is shown in Table 5. Jeong and Miyano [7] used an ANN to incorporate evolutionary information and obtained MCC, overall accuracy, specificity, and sensitivity of 0.39, 80.20%, 91.04%, and 43.40%, respectively. The MCC of their proposed method was further improved to 0.41 based on a weighted profile approach. In addition, Kumar *et al*. developed PPRint [8], which incorporated PSSM profiles in an SVM model, and attained MCC, overall accuracy, specificity, and sensitivity of 0.45, 81.16%, 89.55%, and 53.05%, respectively. Compared to these approaches, our method not only achieves high overall accuracy but also significantly improves the sensitivity by 26.90%~36.55% using five-fold cross-validation. Moreover, RNAProB achieves 0.68 in MCC, compared to 0.45 by PPRint and 0.41 by Jeong and Miyano.

**Table 5:** Performance comparison of different approaches using five-fold cross-validation for the benchmark data sets.

**Data set**	**Method**	**Spec. (%)**	**Sens. (%)**	**Acc (%)**	**MCC**	**Threshold**
RBP86	Jeong 2006	91.04	43.4	80.2	0.39 (0.41)*	--
	PPRint	89.55	53.05	81.16	0.45	--
	**RNAProB **^§^	**90.36**	**79.95**	**87.99**	**0.68**	**0.36**
	**RNAProB **^#^	**90.01**	**79.64**	**87.65**	**0.67**	**0.36**

RBP109	RNABindR	93.00	38.00	84.80	0.35	--
	**RNAProB **^§^	**93.88**	**64.62**	**89.70**	**0.58**	**0.35**
	**RNAProB **^#^	**94.14**	**60.63**	**89.36**	**0.56**	**0.35**

RBP107	BindN-PCP^&^	69.84	66.28	69.32	0.27	--
	BindN-ALL^&^	75.70	65.78	74.25	--	--
	PPRint	75.54	70.09	75.43	0.32	--
	**RNAProB **^§^	**80.87**	**77.14**	**80.44**	**0.42**	**0.11**
	**RNAProB **^#^	**80.65**	**73.62**	**79.84**	**0.40**	**0.12**

^§ ^presents the performance by five-fold cross-validation.

^# ^denotes the performance by a three-way data split procedure.

* indicates the performance of weighted profiles by Jeong and Miyano [7].

^&^BindN-PCP represents the results based only on physicochemical properties, while BindN-ALL shows the performance using physicochemical properties, relative solvent accessible surface area, and BLAST results.

#### 2. Performance comparison with RNABindR on the RBP109 data set

**Figure 5:**
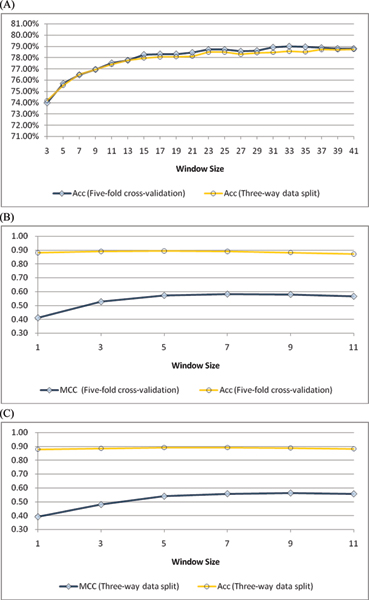
(A) Accuracy with respect to different sliding window sizes using five-fold cross-validation and three-way data split for the RBP109 data set, respectively. (B) The performance of the RBP109 data set with different smoothing window sizes by five-fold cross-validation. (C) The performance of the RBP109 data set with different smoothing window sizes by three-way data split.

Figure 5 illustrates the experimental results of different sliding and smoothing window sizes on the RBP109 data set. Similar to the RBP86 data set, the RBP109 data set exhibits a slow growth in the prediction performance when sliding window size *w *is greater than 25 or smoothing window size *ws *is larger than 7. Thus, we also select *w *as 25 and *ws *as 7 for this data set. Table 4 shows that RNAProB attains 0.58, 89.70%, 93.88%, and 64.62% in MCC, overall accuracy, specificity, and sensitivity using five-fold cross-validation (with *w *= 25, *ws *= 7, *C *= 4, *γ *= 0.015625, *w*_1 _= 4, *w*_-1 _= 1, and threshold value = 0.35), respectively. Besides, evaluated by three-way data split, our method obtains MCC, overall accuracy, specificity, and sensitivity of 0.56, 89.36%, 94.14%, and 60.63% (with *w *= 25, *ws *= 7, *C *= 8, *γ *= 0.015625, *w*_1 _= 4, *w*_-1 _= 1, and threshold value = 0.35), respectively. The prediction performance of different window sizes and parameters on the RBP109 data set is detailed in the supplementary material [see Additional file 5].

Table 5 illustrates the performance comparison with RNABindR [6,11], a Naïve Bayes based method developed on the same data set. Using five-fold cross-validation, RNAProB achieves 0.58, 89.70%, 93.88%, and 64.62% in MCC, overall accuracy, specificity, and sensitivity, respectively, compared favourably to 0.35, 84.80%, 93.00%, and 38.00% by RNABindR. Particularly, our method significantly outperforms RNABindR by 26.62% in terms of sensitivity.

#### 3. Performance comparison with other approaches on the RBP107 data set

**Figure 6:**
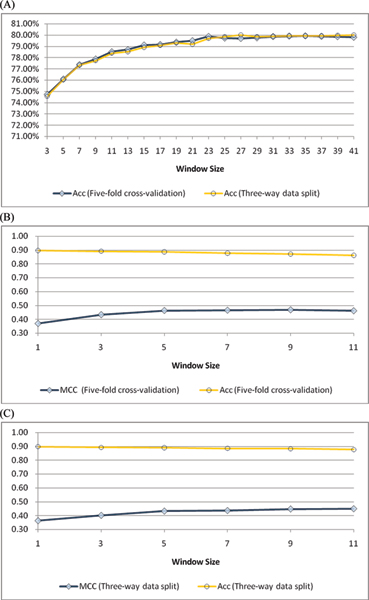
(A) Accuracy with respect to different sliding window sizes using five-fold cross-validation and three-way data split for the RBP107 data set, respectively. (B) The performance of the RBP107 data set with different smoothing window sizes by five-fold cross-validation. (C) The performance of the RBP107 data set with different smoothing window sizes by three-way data split.

The prediction performance of different sliding and smoothing window sizes on the RBP107 data set is demonstrated in Figure 6. Similar to the RBP86 data set, we observe that the overall accuracy converges as sliding window size is greater than 25 on the RBP107 data set in Figure 6(B). Moreover, the MCC shows a slight peak when the smoothing window size reaches 7 in Figure 6(C). Thus RNAProB also selects *w *as 25 and *ws *as 7 for this data set. As illustrated in Table 4, our method reaches 0.42, 80.44%, 80.87%, and 77.14% in MCC, overall accuracy, specificity, and sensitivity by five-fold cross-validation (with *w *= 25, *ws *= 7, *C *= 4, *γ *= 0.015625, *w*_1 _= 4, *w*_-1 _= 1, and threshold value = 0.11), respectively. In addition, RNAProB also attains MCC, overall accuracy, specificity, and sensitivity of 0.40, 79.84%, 80.65%, and 73.62% by three-way data split (with *w *= 25, *ws *= 7, *C *= 8, *γ *= 0.015625, *w*_1 _= 4, *w*_-1 _= 1, and threshold value = 0.12), correspondingly. The detailed experimental results on the RBP109 data set are summarized in the supplementary material [see Additional file 6].

Table 5 compares the performance of RNAProB with other approaches on the RBP107 data set. Based on physicochemical properties, BindN (i.e. referred to as BindN-PCP in Table 5) attains MCC, overall accuracy, specificity, and sensitivity of 0.27, 69.32%, 69.84%, and 66.28%, respectively [9]. Incorporated with more biological features, BindN (i.e. denoted as BindN-ALL in Table 5) further improves specificity and accuracy by 5.86% and 4.93% with a slight decrease in sensitivity [18]. PPRint improves sensitivity to 70.09% with the other measures performed comparable to those of BindN-ALL. Our method significantly outperforms the-state-of-the-art approaches by 0.10, 5.10%, 5.33%, and 7.05% in MCC, overall accuracy, specificity, and sensitivity, respectively. This demonstrates that RNAProB not only achieves accurate performance, but also substantially improves sensitivity in the prediction of RNA-binding sites.

## Discussion

### Physicochemical preferences of interacting and non-interacting residues

**Figure 7:**
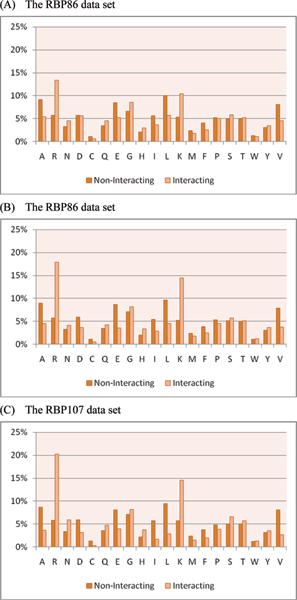
Amino acid compositions of interacting and non-interacting residues in the benchmark data sets.

In this section, we examine the physicochemical properties of RNA interacting and non-interacting residues. Figure 7(A), (B), and 7(C) show the amino acid compositions of interacting and non-interacting residues in the RBP86, RBP109, and RBP107 data sets, respectively. It is observed that interacting and non-interacting residues show preferences for different amino acids. RNA interacting residues tend to have high compositions for Arginine (R), Asparagine (N), Glutamine (Q), Glycine (G), Histidine (H), and Lysine (K). For example, there are relatively high proportions for Arginine (R) and Lysine (K), which may interact with negatively charged RNA with their positive side chains. In addition, the smallest amino acid, Glycine (G), also has a high composition in interacting residues because it rotates easily and provides flexibility to interact with RNA molecules. Moreover, positively charged Histidine (H) can have an aromatic interaction with RNA molecules due to its specific pKa value and imidazole ring. On the other hand, non-interacting residues show slight preferences for Alanine (A), Aspartic acid (D), Glutamic acid (E), Isoleucine (I), Leucine (L), Phenylalanine (F), and Valine (V). Cysteine (C), Aspartic acid (D), and Glutamic acid (E) are favoured by non-interacting residues because of their negatively charged side chains. In addition, although Kumar *et al. *[8] reported that Aspartic acid (D) showed no preference for interacting or non-interacting residues in their main data set (i.e., the RBP86 data set in our study), we observed that the Aspartic acid (D) composition of non-interacting residues is significantly higher than that of interacting residues in both of the RBP109 and RBP107 data sets. Our analysis indicates that the finding from Kumar *et al*. could be a bias from the data set.

**Figure 8:**
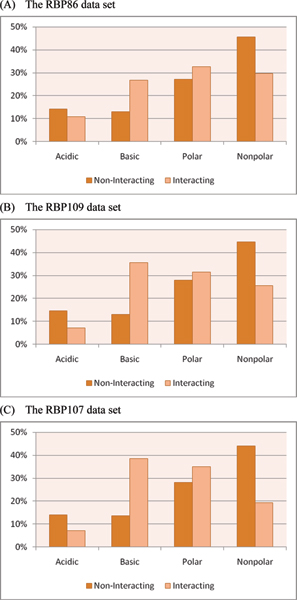
Grouped amino acid compositions of interacting and non-interacting residues in the benchmark data sets.

To further analyze the physicochemical properties of the RNA interacting and non-interacting residues, each amino acid is classified into one of the four groups: acidic (DE), basic (HKR), polar (CGNQSTY), and non-polar (AFILMPVW) [22]. Figure 8 shows the grouped amino acid compositions of interacting and non-interacting residues for the benchmark data sets. It is observed among the three data sets that basic and polar amino acids tend to interact with RNA, and acidic and non-polar amino acids are not favoured by RNA molecules. Particularly, our analysis shows that the compositions of basic amino acids exhibit significantly over-represented patterns for interacting residues.

**Figure 9:**
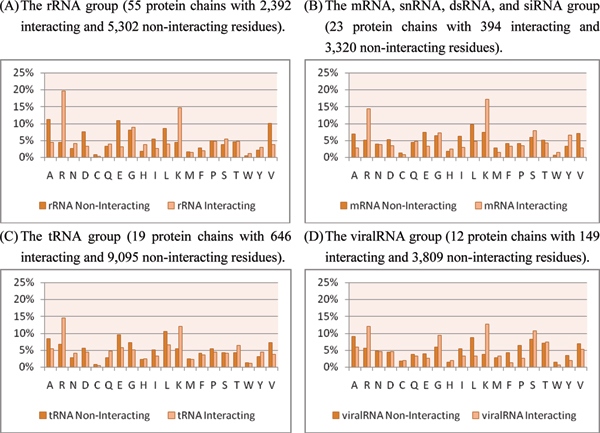
Amino acid compositions of interacting and non-interacting residues in four different RNA groups of the RBP109 data set.

Furthermore, we inspect the amino acid compositions of proteins that interact with different RNA molecules. The proteins in the RBP109 data set are divided into four categories according to the definition in Terribilini *et al *[6]. Figure 9(A), (B), (C), and 9(D) show the amino acid compositions of (A) rRNA, (B) mRNA, snRNA, dsRNA, and siRNA, (C) tRNA, and (D) viralRNA, respectively. It is observed that viralRNA group shows a different amino acid composition compared to the other groups. Proteins that interact with viralRNA evolve fast and induce conformational changes in the active sites. Thus, these proteins exhibit a specific mechanism to interact with viralRNA.

### Comparison of smoothed PSSM and standard PSSM

Here we examine the correlation between interacting and non-interacting residues for both smoothed PSSM and standard PSSM encoding schemes. We incorporate Pearson correlation coefficient (PCC) [23] to measure the correlation between the evolutionary information of interacting and non-interacting for an amino acid. For each amino acid *a*, we use two vectors, *X *and *Y*, to present the sum of PSSM evolutionary information vectors for interacting and non-interacting amino acid *a*, respectively. The Pearson correlation coefficient for a series of *n *measurements for variables *X *and *Y *is defined in Equation (7).

(7) $$\text{PCC}={\text{r}}_{\text{xy}}=(\text{n}\sum {\text{x}}_{\text{i}}{\text{y}}_{\text{i}}-\sum {\text{x}}_{\text{i}}\sum {\text{y}}_{\text{i}})/\sqrt{\text{n}\sum {\text{x}}_{\text{i}}^{2}-{\left(\sum {\text{x}}_{\text{i}}\right)}^{2}}\sqrt{\text{n}\sum {\text{y}}_{\text{i}}^{2}-{\left(\sum {\text{y}}_{\text{i}}\right)}^{2}}$$

**Figure 10:**
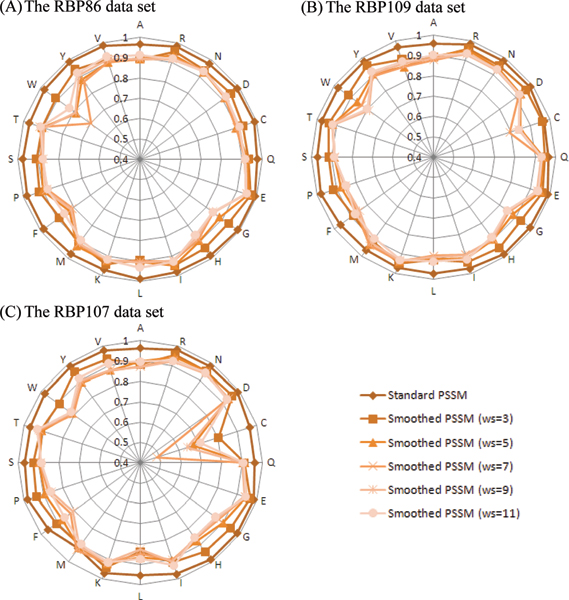
Pearson correlation coefficient between interacting and non-interacting evolutionary vectors generated by different PSSM encoding schemes in the benchmark data sets.

Figure 10 shows the Pearson correlation coefficient between interacting and non-interacting evolutionary information vectors based on different PSSM encoding schemes in the benchmark data sets. It is observed that the correlation coefficients calculated from smoothed PSSM encoding scheme are lower than those from standard PSSM, especially for Cysteine (C) and Tryptophan (W). In Figure 10(A), smoothed PSSM encoding attains lower correlation coefficients not only in interacting residues, such as arginine (R), asparagine (N), glutamine (Q), glycine (G), histidine (H), and lysine (K), but also in non-interacting residues, including alanine (A), aspartic acid (D), glutamic acid (E), isoleucine (I), leucine (L), phenylalanine (F), and valine (V). Similarly, Figure 10(B) and 10(C) also show lower correlation coefficients between interacting and non-interacting residues based on smoothed PSSM encoding. Furthermore, it is observed that the correlation coefficients calculated with smoothing window size *ws *= 7 are usually lower than those generated by other smoothing window sizes. If an encoding scheme leads to a lower Pearson correlation coefficient, it indicates that the encoding scheme can better resolve ambiguity in discriminating interacting residues from non-interacting ones. Our analysis lends support to our assumption that smoothed PSSM encoding scheme can improve the recognition RNA interacting and non-interacting sites by modelling the dependency from surrounding residues.

## Conclusion

We present RNAProB, which combines a new smoothed PSSM encoding scheme with a SVM model for prediction of RNA-binding sites in proteins. In a standard PSSM profile, evolutionary information is calculated based on an assumption that each position is independent of others. However, the correlation or dependency from surrounding residues is incorporated in the proposed smoothed PSSM encoding. Experimental results show that the prediction performance of smoothed PSSM encoding performs better than the state-of-the-art approaches on the benchmark data sets. Evaluated by five-fold cross-validation, RNAProB outperforms the other approaches by 0.10~0.23 in MCC, 4.90%~6.83% in overall accuracy, and 0.88%~5.33% in specificity. Most notably, our method significantly improves sensitivity by 26.90%, 26.62%, and 7.05% for the RBP86, RBP109, and RBP107 data sets, respectively. Performance improvement in RNAProB not only demonstrates that smoothed PSSM can better resolve the ambiguity in discriminating RNA interacting and non-interacting residues, but also supports our assumption that consideration of correlation between neighboring residues can significantly enhance prediction accuracy. To prevent data over fitting, a rigorous three-way data split procedure is incorporated to evaluate our prediction performance. The proposed method can be used in other research topics, such as DNA-binding site prediction, protein-protein interaction, and prediction of posttranslational modification sites.

## Competing interests

The authors declare that they have no competing interests.

## Authors' contributions

CWC developed the method, implemented the system, and drafted the manuscript. ECYS provided biological knowledge, participated in the experimental design, and refined the manuscript. TYS and WLH coordinated this study. All of authors read and approved the final manuscript.

## Supplementary Material

Additional file 1 The RBP86 data set. Click here for file

Additional file 2 The RBP109 data set. Click here for file

Additional file 3 The RBP107 data set. Click here for file

Additional file 4 Detailed experimental results on the RBP86 data set. Click here for file

Additional file 5 Detailed experimental results on the RBP109 data set. Click here for file

Additional file 6 Detailed experimental results on the RBP107 data set. Click here for file
