# Supplementary material for: Predicting RNA-binding sites of proteins using support vector machines and evolutionary information
Source: BMC Bioinformatics. 2008 Dec 12;9(Suppl 12):S6. doi: 10.1186/1471-2105-9-S12-S6 (PMC2638146; doi:10.1186/1471-2105-9-S12-S6)
Supplement: Additional file 1 — The RBP86 data set. [file 1471-2105-9-S12-S6-S1.doc]

# The RBP86 dataset

>2BBV_C

TQTAPVPQQNVPALTRLSQPGLAFLKCAFAPPDFNTDPGKGIPDRFEGKVVTRKDVLNQSINFTANRDTFILIAPTPGVAYWVADVPAGTFPISTTTFNAVNFPGFNSMFGNAAASRSDQVSSFRYASMNVGIYPTSNLMQFAGSITVWKCPVKLSNVQFPVATTPATSALVHTLVGLDGVLAVGPDNFSESFIKGVFSQSVCNEPDFEFSDILEGIQTLPPANVTVATSGQPFNLAAGAEAVSGIVGWGNMDTIVIRVSAPTGAVNSAILKTWACLEYRPNPNAMLYQFGHDSPPCDEVALQEYRTVARSLPVAVIAAQN

000000000000111000100100010000000100000000000000000000000000000000000000000000000000000000000000000000000000000000000000000000000000000000000000000000000000000000000000000000000000000000000000000000000000000000000000000000000000000000000000000000000000000000000000000000000000000000000000000000000000000000000000000000000

>1A9N_D

IRPNHTIYINNMNDKIKKEELKRSLYALFSQFGHVVDIVALKTMKMRGQAFVIFKELGSSTNALRQLQGFPFYGKPMRIQYAKTDSDIISKMR

100001010111011011110100000000000000111111111111101010000000000000000000001001011111111100000

>1ASZ_B

EDTAKDNYGKLPLIQSRDSDRTGQKRVKFVDLDEAKDSDKEVLFRARVHNTRQQGATLAFLTLRQQASLIQGLVKANKEGTISKNMVKWAGSLNLESIVLVRGIVKKVDEPIKSATVQNLEIHITKIYTISETPEALPILLEDASRSEAEAEAAGLPVVNLDTRLDYRVIDLRTVTNQAIFRIQAGVCELFREYLATKKFTEVHTPKLLGAPSEGGSSVFEVTYFKGKAYLAQSPQFNKQQLIVADFERVYEIGPVFRAENSNTHRHMTEFTGLDMEMAFEEHYHEVLDTLSELFVFIFSELPKRFAHEIELVRKQYPVEEFKLPKDGKMVRLTYKEGIEMLRAAGKEIGDFEDLSTENEKFLGKLVRDKYDTDFYILDKFPLEIRPFYTMPDPANPKYSNSYDFFMRGEEILSGAQRIHDHALLQERMKAHGLSPEDPGLKDYCDGFSYGCPPHAGGGIGLERVVMFYLDLKNIRRASLFPRDPKRLRP

0000000000000000000000000000000000000000000000000111111111010000000000101010000000000001000101100000000000011111111000001010000000000011111101100100000011111111111000100000000000000000000000000000000000000000000111111100000000000000110110000000000000000000010111111010000000000000000000000000000000000000000000000000000000000000000000000000000000000000011111001000000000000000000000001011100000000000000000000000010000000000000000000100000110000000000000000000000100000000010000000001011000

>1AV6_A

VVSLDKPFMYFEEIDNELDYEPESANEVAKKLPYQGQLKLLLGELFFLSKLQRHGILDGATVVYIGSAPGTHIRYLRDHFYNLGVIIKWMLIDGRHHDPILNGLRDVTLVTRFVDEEYLRSIKKQLHPSKIILISDVRSPSTADLLSNYALQNVMISILNPVASSLKWRCPFPDQWIKDFYIPHGNKMLQPFAPSYSAEMRLLSIYTGENMRLTRVTKSDAVNYEKKMYYLNKIVRNKVVVNFDYPNQEYDYFHMYFMLRTVYCNKTFPTTKAKVLFLQQSIFRFLNIP

0000000000000000000000000000010111111111000000000000000000000000000111100000000000000000000000000000000000000000000000000000000000000001010100000000000000000000000000101000000000000000000001011100111010000000000000000000000000000000000000000000000000000000000000000000000000000000000000100

>1B23_P

AKGEFIRTKPHVNVGTIGHVDHGKTTLTAALTYVAAAENPNVEVKDYGDIDKAPEERARGITINTAHVEYETAKRHYSHVDCPGHADYIKNMITGAAQMDGAILVVSAADGPMPQTREHILLARQVGVPYIVVFMNKVDMVDDPELLDLVEMEVRDLLNQYEFPGDEVPVIRGSALLALEEMHKNPKTKRGENEWVDKIWELLDAIDEYIPTPVRDVDKPFLMPVEDVFTITGRGTVATGRIERGKVKVGDEVEIVGLAPETRKTVVTGVEMHRKTLQEGIAGDNVGLLLRGVSREEVERGQVLAKPGSITPHTKFEASVYILKKEEGGRHTGFFTGYRPQFYFRTTDVTGVVRLPQGVEMVMPGDNVTFTVELIKPVALEEGLRFAIREGGRTVGAGVVTKILE

010000000000000000000000000000000000000000000000000111100010001100000000000000000000001101100000000000000000000000000000000000000000000000000000000000000000000000000000000000000000000000000000000000000000000000000000000000000011111111001010000000000000000000000000000111111110000000001111100000000001000000000000000000000000000001111111111110100001110000000000000000000000001100000000000011110000000000000

>1B7F_B

SNTNLIVNYLPQDMTDRELYALFRAIGPINTCRIMRDYKTGYSYGYAFVDFTSEMDSQRAIKVLNGITVRNKRLKVSYARPGGESIKDTNLYVTNLPRTITDDQLDTIFGKYGSIVQKNILRDKLTGRPRGVAFVRYNKREEAQEAISALNNVIPEGGSQPLSVRLA

00010101111100000000000000000000101111100111110100000000000000000000001111101111111001001101111101000000000000000001111110000000011101010000000000000000000000000000111

>1C0A_A

MRTEYCGQLRLSHVGQQVTLCGWVNRRRDLGSLIFIDMRDREGIVQVFFDPDRADALKLASELRNEFCIQVTGTVRARDEKNINRDMATGEIEVLASSLTIINRADVLPLDSNHVNTEEARLKYRYLDLRRPEMAQRLKTRAKITSLVRRFMDDHGFLDIETPMLTKATPEGARDYLVPSRVHKGKFYALPQSPQLFKQLLMMSGFDRYYQIVKCFRDEDLRADRQPEFTQIDVETSFMTAPQVREVMEALVRHLWLEVKGVDLGDFPVMTFAEAERRYGSDKPDLRNPMELTDVADLLKSVEFAVFAGPANDPKGRVAALRVPGGASLTRKQIDEYGNFVKIYGAKGLAYIKVNERAKGLEGINSPVAKFLNAEIIEDILDRTAAQDGDMIFFGADNKKIVADAMGALRLKVGKDLGLTDESKWAPLWVIDFPMFEDDGEGGLTAMHHPFTSPKDMTAAELKAAPENAVANAYDMVINGYEVGGGSVRIHNGDMQQTVFGILGINEEEQREKFGFLLDALKYGTPPHAGLAFGLDRLTMLLTGTDNIRDVIAFPKTTAAACLMTEAPSFANPTALAELSIQVVK

000000000000000000000000011111111010100000000101011000000000000110000000000001001111011000001000000000000011111011111111110000000000000000000000000000000000000000000000111111000000000000000001111100000000000000000000101111111000001000000000000000000000000000000000000000000000000000000000000000000000000000000000000000000000000000000000000000110000000000000000000000000000000000000000000000000000000100010000000000000000000000000000000000000000011111100000000000000000000000000000000000000000000000000000000000000100000000000000000000001000000001101000000111111010000000000000000000000

>1C9S_L

TNSDFVVIKALEDGVNVIGLTRGADTRFHHSEKLDKGEVLIAQFTEHTSAIKVRGKAYIQTRHGVIESEG

0000000000001111010100000111111111100100000000000001010000000000000000

>1CVJ_H

ASLYVGDLHPDVTEAMLYEKFSPAGPILSIRVCRDMITRRSLGYAYVNFQQPADAERALDTMNFDVIKGKPVRIMWSQRDPSLRKSGVGNIFIKALYDTFSAFGNILSCKVVCSKGYGFVHFETQEAAEFKS

011101100000010000000000000011111111101001110111000000000000000000000000101111111011101001110000000000000111110000001011100000000111

>1DDL_C

NTKPSLLPPPVGNPPPVISYPFQITLASLGTEDAADSVSIASNSVLATYTALYRHAQLKHLKATIHPTYMAPKYPTSVALVWVPANSTATSTQVLDTYGGLHFCIGGSVNSVKPIDVEANLTNLNPIIKASTTFTDTPKLLYYSKAQATAPTSPTCYLTIQGQIELSSPLLQASS

0000000000000000000000000000000000000000000000000000000000000000000000000000000000000000000000101111111000000000000000010110000000000000000000000000000000000000000000000000000

>1DFU_P

MFTINAEVRKEQGKGASRRLRAANKFPAIIYGGKEAPLAIELDHDKVMNMQAKAEFYSEVLTIVVDGKEIKVKAQDVQRHPYKPKLQHIDFVRA

0000000010111111110010000011101100001110000000000000000000000000000000000001010000000011110000

>1DI2_B

MPVGSLQELAVQKGWRLPEYTVAQFTITCRVETFVETGSGTSKQVAKRVAAEKLLTKFKT

101110100000000010000000000000000000000000010011000000000000

>1DRZ_A

PETRPNHTIYINNLNEKIKKDELKKSLHAIFSRFGQILDILVSRSLKRGQAFVIFKEVSSATNALRSQGFPFYDKPRIQYAKTDSDIIAKK

0110010101011101101000000000000000000000101111111101010000000000000000000010101111111110000

>1DUL_A

FDLNDFLEQKVLVREAIINSTKERAKPEIIKGSRKRRIAAGSGQVQDVNRLLKQFDDQRKK

0000000000001111111111110000000011111101111000000000000000000

>1E6T_C

ASNFTQFVLVDNGGTGDVTVAPSNFANGVAEWISSNSRSQAYKVTCSVRQSSAQNRKYTIKVEVPKVATQTVGGVELPVAAWRSYLNMELTIPIFATNSDCELIVKAMQGLLKDGNPIPSAIAANSGIY

000000000000000000000000000011000000000000101110111100001011101000000000000000000010111010100000000000000000000000000000000000000

>1E7K_B

VNPKAYPLADAHLTKKLLDLVQQSCNYKQLRKGANEATKTLNRGISEFIVMAADAEPLEIILHLPLLCEDKNVPYVFVRSKQALGRACGVSRPVIACSVTIKEGSQLKQQIQSIQQSIERLLV

000000000000000000000000000000111111101000100000000001111001100000000000000000001000000001111111000000000000000000000000000

>1EC6_A

MKELVEIAVPENLVGAILGKGGKTLVEYQELTGARIQISKKGEFLPGTRNRRVTITGSPAATQAAQYLISQRVTYEQGVRASNPQKV

000000000011111111111111110010000011111111010000101000000000000000000001000100010000111

>1EFW_B

MRRTHYAGSLRETHVGEEVVLEGWVNRRRDLGGLIFLDLRDREGLVQLVAHPASPAYATAERVRPEWVVRAKGLVRLRPEPNPRLATGRVEVELSALEVLAEAKTPPFPVDAGWRGEEEKEASEELRLKYRYLDLRRRRMQENLRLRHRVIKAIWDFLDREGFVQVETPFLTKSTPEGARDFLVPYRHEPGLFYALPQSPQLFKQMLMVAGLDRYFQIARCFRDEDLRADRQPDFTQLDLEMSFVEVEDVLELNERLMAHVFREALGVELPLPFPRLSYEEAMERYGSDKPDLRFGLELKEVGPLFRQSGFRVFQEAESVKALALPKALSRKEVAELEEVAKRHKAQGLAWARVEEGGFSGGVAKFLEPVREALLQATEARPGDTLLFVAGPRKVAATALGAVRLRAADLLGLKREGFRFLWVVDFPLLEWDEEEEAWTYMHHPFTSPHPEDLPLLEKDPGRVRALAYDLVLNGVEVGGGSIRIHDPRLQARVFRLLGIGEEEQREKFGFFLEALEYGAPPHGGIAWGLDRLLALMTGSPSIREVIAFPKNKEGKDPLTGAPSPVPEEQLRELGLMVVRP

0000000000000000000000000011111111010100000000100011100000001001100000000000011111011000001000000000000011100000001010000010010000000000000000000000000000000000000000000000000000000000000000000000000000000000000000000000000000000000000000000000000000000000000000000000000000000000000000000000000000000000000000000000000000000000000000000011001000000000000000000000000000000000000000000000000000000000000001000000000000000000000000000000000000000000000000000000000000000000000000000000000000000000000000000000000000000000000000000000000000000000000000011000000000000000000000000000

>1F7U_A

ASTANMISQLKKLSIAEPAVAKDSHPDVNIVDLMRNYISQELSKISGVDSSLIFPALEWTNTMERGDLLIPIPRLRIKGANPKDLAVQWAEKFPCGDFLEKVEANGPFIQFFFNPQFLAKLVIPDILTRKEDYGSCKLVENKKVIIEFSSPNIAKPFHAGHLRSTIIGGFLANLYEKLGWEVIRMNYLGDWGKQFGLLAVGFERYGNEEALVKDPIHHLFDVYVRINKDIEEEGDSIPLEQSTNGKAREYFKRMEDGDEEALKIWKRFREFSIEKYIDTYARLNIKYDVYSGESQVSKESMLKAIDLFKEKGLTHEDKGAVLIDLTKFNKKLGKAIVQKSDGTTLYLTRDVGAAMDRYEKYHFDKMIYVIASQQDLHAAQFFEILKQMGFEWAKDLQHVNFGMVQGMSTRKGTVVFLDNILEETKEKMHEVMKKNENKYAQIEHPEEVADLVGISAVMIQDMQGKRINNYEFKWERMLSFEGDTGPYLQYAHSRLRSVERNASGITQEKWINADFSLLKEPAAKLLIRLLGQYPDVLRNAIKTHEPTTVVTYLFKLTHQVSSCYDVLWVAGQTEELATARLALYGAARQVLYNGMRLLGLTPVERM

000000000000000000000000000000000000000000000000000000000110110010001010111100000000000000000000000000000011000000000000000000000000000000000000000000010000000000000000000000000000000000001111110000000000000000000000000000000000000000000000000000000010000000000000000000000000000000000000000010000000000000000000000011110100000000100111011110111110100000000000000000000001111110000000000000000000000000101000000000000000000000010011001001000000000000010000000000111111110000010001111101100110110011111000000000000000000000000000000000000000001010110010011011001111111111110000000000010000000000000000000111

>1F7Y_A

PITKEEKQKVQEFARFPGDTGSTEVQVALLTLRINRLSEHLKVHKKDHHSHRGLLVGQRRRLLRYLQREDPERYRLIEKLGI

1101001100000011011111111100100010011001001111111111110001001101100110001000000000

>1F8V_A

NRRNKARKVVSRSTALVPMAPASQRTGPAPRKPRKRNQALVRNPRLTDAGLAFLKCAFAAPDFSVDPGKGIPDNFHGRTLAIKDCNTTSVVFTPNTDTYIVVAPVPGFAYFRAEVAVGAQPTTFVGVPYPTYATNFGAGSQNGLPAVNNYSKFRYASMACGLYPTSNMMQFSGSVQVWRVDLNLSEAVNPAVTAITPAPGVFANFVDKRINGLRGIRPLAPRDNYSGNFIDGAYTFAFDKSTDFEWCDFVRSLEFSESNVLGAATAMKLLAPGGGTDTTLTGLGNVNTLVYKISTPTGAVNTAILRTWNCIELQPYTDSALFQFSGVSPPFDPLALECYHNLKMRFPVAVSSREN

1010111100000000000000000000000110000000000000000000000000000000000000000000000000000000000000000000000000000000000000000000000000000000000000000000000000000000000000000000000000000000000000000000000000000000000000000000000000000000000000000000000000000000000000000000000000000000000000000000000000000000000000000000000000000000000000000000000000000000000

>1FEU_D

MEYRLKAYYREGEKPSALRRAGKLPGLMYNRHLNRKVYVDLVEFDKVFRQASIHHVIVLELPDGQSLPTLVRQVNLDKRRRRPEHVDFFVLSDEPVEMYVPLRFVGTPAGVRAGGVLQEIHRDILVKVSPRNIPEFIEVDVSGLEIGDSLHASDLKLPPGVELAVSPEETIAAVVPPEDVEKLAEEAAA

000000000110101100100000111110001101000000000000000000000000000000000000101001100001111000000000000000000000000000000000000000000000000000000000000000000000000000000000000000000000000000000

>1FJG_B

VKELLEAGVHFGHERKRWNPKFARYIYAERNGIHIIDLQKTMEELERTFRFIEDLAMRGGTILFVGTKKQAQDIVRMEAERAGMPYVNQRWLGGMLTNFKTISQRVHRLEELEALFASPEIEERPKKEQVRLKHELERLQKYLSGFRLLKRLPDAIFVVDPTKEAIAVREARKLFIPVIALADTDSDPDLVDYIIPGNDDAIRSIQLILSRAVDLIIQARGGVVEPSPSYALVQEAE

000000000000000111111000000010100000000000000000000000000000000000000000000000000000000001111110110011011001000000000000000000100000011001101100000000000000000000000100110010000000000000000000000000000000000000000000000000000000000000000

>1FJG_D

GRYIGPVCRLCRREGVKLYLKGERCYSPKCAMERRPYPPGQHGQKRARRPSDYAVRLREKQKLRRIYGISERQFRNLFEEASKKKGVTGSVFLGLLESRLDNVVYRLGFAVSRRQARQLVRHGHITVNGRRVDLPSYRVRPGDEIAVAEKSRNLELIRQNLEAMKGRKVGPWLSLDVEGMKGKFLRLPDREDLALPVQENLVIEFYSR

1111111110011100000111111010101011101111110110010011110111011001100011111011000001110110000000000010000000000011111011001111000001111111110100000000011001110010000000000000000000000000000000000000000000011111

>1FJG_L

PTINQLVRKGREKVRKKSKVPALKGAPFRRGVCTVVRTVTPKKPNSALRKVAKVRLTSGYEVTAYIPGEGHNLQEHSVVLIRGGRVKDLPGVRYHIVRGVYDAAGVKDRKKSRSKYGTKKPKEAA

11111101111111111111100111111110000010011111111111001000110010001111111000000001111111111111101011111000000111111111111110100

>1FJG_M

ARIAGVEIPRNKRVDVALTYIYGIGKARAKEALEKTGINPATRVKDLTEAEVVRLREYVENTWKLEGELRAEVAANIKRLMDIGCYRGLRHRRGLPVRGQRTRTNARTRKGPRKTVAGKKKAPRK

00000000001110110011111111111000000000000010000000000000000000000000110110011011000001100110101111111111111111111111111101111

>1FJG_S

PRSLKKGVFVDDHLLEKVLELNAKGEKRLIKTWSRRSTIVPEMVGHTIAVYNGKQHVPVYITENMVGHKLGEFAPTRTYRGHGK

111111011010100010000000000000111111010000000000001111110000000000001111111111111111

>1FJG_T

RNLSALKRHRQSLKRRLRNKAKKSAIKTLSKKAVQLAQEGKAEEALKIMRKAESLIDKAAKGSTLHKNAAARRKSRLMRKVRQLLEAAGAPLIGGGLSA

111111111111111111111111101110110000000000000000111011101100101111111111111101110110000000000111111

>1G1X_F

MRRYEVNIVLNPNLDQSQLALEKEIIQRALENYGARVEKVEELGLRRLAYPIAKDPQGYFLWYQVEMPEDRVNDLARELRIRDNVRRVMVVKSQEPFL

01010000000000000000000000000000000000000000000000000000000000000000110110000000000000001111100000

>1G1X_G

PITKEEKQKVIQEFARFPGDTGSTEVQVALLTLRINRLSEHLKVHKKDHHSHRGLLMMVGQRRRLLRYLQREDPERYREIVEKLGLRG

1100000000000001101111110010000001001100100010011111111011001001100100110001000000000000

>1G1X_H

DLRDYRNVEVLKRFLSKILPRTGLSGKEQRILAKTIKRARILGLLPFT

000000000000001011111000000011001101110110000111

>1G2E_A

SKTNLIVNYLPQNMTQEEFRSLFGSIGEIESCKLVRDKITGQSLGYGFVNYIDPKDAEKAINTLNGLRLQTKTIKVSYARPSSASIRDANLYVSGLPKTMTQKELEQLFSQYGRIITSRILVDQVTGVSRGVGFIRFDKRIEAEEAIKGLNGQKPSGATEPITVKFA

00011101110100000000000000000000101101000011110100000000000000000000001111101111111101110101011100000000000000000001101111000000011101010000000000000000000000000000111

>1H38_D

NTINIAKNDFSDIELAAIPFNTLADHYGERLAREQLALEHESYEMGEARFRKMFERQLKAGEVADNAAAKPLITTLLPKMIARINDWFEEVKAKRGKRPTAFQFLQEIKPEAVAYITIKTTLACLTSADNTTVQAVASAIGRAIEDEARFGRIRDLEAKHFKKNVEEQLNKRVGHVYKKAFMQVVEADMLSKGLLGGEAWSSWHKEDSIHVGVRCIEMLIESTGMVSLHRQNAGVVGQDSETIELAPEYAEAIATRAGALAGISPMFQPCVVPPKPWTGITGGGYWANGRRPLALVRTHSKKALMRYEDVYMPEVYKAINIAQNTAWKINKKVLAVANVITKWKHCPVEDIPAIEALTAWKRAAAAVYRKDKARKSRRISLEFMLEQANKFANHKAIWFPYNMDWRGRVYAVSMFNPQGNDMTKGLLTLAKGKPIGKEGYYWLKIHGANCAGVDKVPFPERIKFIEENHENIMACAKSPLENTWWAEQDSPFCFLAFCFEYAGVQHHGLSYNCSLPLAFDGSCSGIQHFSAMLRDEVGGRAVNLLPSETVQDIYGIVAKKVNEILQADAINGTDNEVVTVTDENTGEISEKVKLGTKALAGQWLAYGVTRSVTKRSVMTLAYGSKEFGFRQQVLEDTIQPAIDSGKGLMFTQPNQAAGYMAKLIWESVSVTVVAAVEAMNWLKSAAKLLAAEVKDKKTGEILRKRCAVHWVTPDGFPVWQEYKKPIQTRLNLMFLGQFRDSEIDAHKQESGIAPNFVHSQDGSHLRKTVVWAHEKYGIESFALIHDSFGTIPADAANLFKAVRETMVDTYESCDVLADFYDQFADQLHESQLDKMPALPAKGNLNLRDILESDFAFA

00000000000000000000000000000000000000000000000010001001100001000000000000000000000000000000000000000000000000000000000000000000000000000000000000000000000000110010000000001000000000000000000000000000000000000000000000000000000000000000000000000000000000000000000011000000000000000000000000000000101000000000000000000000000000000000000000000000000000000000000000000000000100010010000000000000000000000001110101000100000000000000000000000000000000000000000000000000000000000000000000000000000000000000000000000000000000000000000000000000000000000000000000000000000000000000000000000000000000000000000000000000000000000010111110111001000000000000000000000000000000000000000000000000000000000000000100000000000000000000000001010000000000000000000101100110011001000000000000000000000000001000000000000000000000000000000000000000000000000000000000000000000000000

>1H3E_A

HTPEEALALLKRGAEEIVPEEELLAKLKEGRPLTVKLGADPTRPDLHLGHAVVLRKMRQFQELGHKVVLIIGDFTGMIGDPSGRSKTRPPLTLEETRENAKTYVAQAGKILRQEPHLFELRYNSEWLEGLTFKEVVRLTSLMTVAQMLEREDFKKRYEAGIPISLHELLYPFAQAYDSVAIRADVEMGGTDQRFNLLVGREVQRAYGQSPQVCFLMPLLVGLDGREKMSKSLDNYIGLTEPPEAMFKKLMRVPDPLLPSYFRLLTDLEEEEIEALLKAGPVPAHRVLARLLTAAYALPQIPPRIDRAFYESLGYAWEAFGRDKEAGPEEVRRAEARYDEVAKGGIPEEIPEVTIPASELKEGRIWVARLFTLAGLTPSNAEARRLIQNRGLRLDGEVLTDPMLQVDLSRPRILQRGKDRFVRVRLSD

0000000000000000000000000000000000000000000000000000000000000000000000000000000000000100000000000000000000000000000000000000000000000000000000000000000000001001111000000000000000000000000000000000000000000000000000000000001000000000000000000000000001111100000000000000000000000001100100000000000000000000000000000000000000000000000000001000100000000000000001111011100100100000111110111011111100000000010100000000001110100000000

>1H4S_B

KGLTPQSQDFSEWYLEVIQKAELADYGPVRGTIVVRPYGYAIWENIQQVLDRMFKETGHQNAYFPLFIPMSFLRKEAEHVEGFSPELAVVTHAGGEELEEPLAVRPTSETVIGYMWSKWIRSWRDLPQLLNQWGNVVRWEMRTRPFLRTSEFLWQEGHTAHATREEAEEEVRRMLSIYARLAREYAAIPVIEGLKTEKEKFAGAVYTTTIEALMKDGKALQAGTSHYLGENFARAFDIKFQDRDLQVKYVHTTSWGLSWRFIGAIIMTHGDDRGLVLPPRLAPIQVVIVPIYKDESRERVLEAAQGLRQALLAQGLRVHLDDRDQHTPGYKFHEWELKGVPFRVELGPKDLEGGQAVLASRLGGKETLPLAALPEALPGKLDAFHEELYRRALAFREDHTRKVDTYEAFKEAVQEGFALAFHCGDKACERLIQEETTATTRCVPFEAEPEEGFCVRCGRPSAYGKRVVFAKAY

11100000000000000000000000000000000000000000000000000000000000000000000000000000000000000000000000000000000000000000000000000000000000000000000000000000000000000000000000000000000000000000000000000000000000000000000000000000000000000000000000000000000000000000000000000000100000000000000000111100000000000000000000000000000101111111110110000010111111100001101000001000000000000000000000000000000000000000000000000000000000000000000000000000000000000000000000000000000000000

>1HQ1_A

GFDLNDFLEQLRQDDKVLVRMEAIINSMTMKERAKPEIIKGSRKRRIAAGSGMQVQDVNRLLKQFDDMQRMMKKMK

0000000000000000001101111111111110000000011111101111100000000000000000000000

>1I6U_A

SLMDPLANALNHISNCERVGKKVVYIKPASKLIGRVLKVQDNGYIGEFEFIEDGRAGIFKVELIGKINKCGAIKPRFPVKKFGYEKFEKRYLPARDFGILIVSTTQGVSHEEAKKRGLGGRLLAYVY

0101100000000000000000000001111100100000000000000000011100000000000000000001011111000000000000000000001111110000000011111010000

>1IVS_B

MDLPKAYDPKSVEPKWAEKWAKNPFVANPKSGKPPFVIFMPPPNVTGSLHMGHALDNSLQDALIRYKRMRGFEAVWLPGTDHAGIATQVVVERLLLKEGKTRHDLGREKFLERVWQWKEESGGTILKQLKRLGASADWSREAFTMDEKRSRAVRYAFSRYYHEGLAYRAPRLVNWCPRCETTLSDLEVETEPTPGKLYTLRYEVEGGGFIEIATVRPETVFADQAIAVHPEDERYRHLLGKRARIPLTEVWIPILADPAVEKDFGTGALKVTPAHDPLDYEIGERHGLKPVSVINLEGRMEGERVPEALRGLDRFEARRKAVELFREAGHLVKEEDYTIALATCSRCGTPIEYAIFPQWWLRMRPLAEEVLKGLRRGDIAFVPERWKKVNMDWLENVKDWNISRQLWWGHQIPAWYCEDCQAVNVPRPERYLEDPTSCEACGSPRLKRDEDVFDTWFSSALWPLSTLGWPEETEDLKAFYPGDVLVTGYDILFLWVSRMEVSGYHFMGERPFKTVLLHGLVLDEKGQKMSKSKGNVIDPLEMVERYGADALRFALIYLATGGQDIRLDLRWLEMARNFANKLYNAARFVLLSREGFQAKEDTPTLADRFMRSRLSRGVEEITALYEALDLAQAAREVYELVWSEFCDWYLEAAKPALKAGNAHTLRTLEEVLAVLLKLLHPMMPFLTSELYQALTGKEELALEAWPEPGGRDEEAERAFEALKQAVTAVRALKAEAGLPPAQEVRVYLEGETAPVEENLEVFRFLSRADLLPERPAKALVKAMPRVTARMPLEGLLDVEEWRRRQEKRLKELLALAERSQRKLASPGFREKAPKEVVEAEEARLKENLEQAERIREALSQIG

0001111000000000000000000000000000000000000000000000000000000000000000000000000000000000000000000000000000000000000000000000000000000000000000000000000000000000000000000000000000000000110001000000100000000000000011110000000000000000000000000000000000000000001111111111110000011110100010000000000000000000000000000000000000000000000000001111000000000000000000000000000000000000000000001100100110010100000000000000000000000000000000000000000000000000000000000000000000000000000000000000000010001000000000000000000000000000000001100000000000000000000000000000111110111101011011011101100111111010000000000000000000000000000000000000100100100110010001100110010001000000000000000000000000000000000000000000000000000000000000000000001001100110111111000000000000000000000100100000000000001000000000000000000000010010001000110110010011111011100100010011011000000000000000

>1JBR_A

ATWTCINQQLNPKTNKWEDKRLLYSQAKAESNSHHAPLSDGKTGSSYPHWFTNGYDGNGKLIKGRTPIKFGKADCDRPPKHSQNGMGKDDHYLLEFPTFPDGHDYKFDSKKPKEDPGPARVIYTYPNKVFCGIVAHQRGNQGDLRLCSH

00000000000000000000000000000000000000011111110011010110000001001100000000000000101000000000000000000000000001101000000000000000000000000000011100000

>1JID_A

AARSPADQDRFICIYPAYLNNKKTIAEGRRIPISKAVENPTATEIQDVCSAVGLNVFLEKNKMYSREWNRDVQYRGRVRVQLKQEDGSLCLVQFPSRKSVMLYAAEMIPKLKTR

000000001111111001000011110011111110000000000000000000000000000111000000000010100010000000000000110000000000000000

>1JJ2_1

GKKSKATKKRLAKLDNQNSRVPAWVMLKTDERNHKRRHWRRNDTDE

1111111111111111111111011011111111111111111111

>1JJ2_2

MQMPRRFNTYCPHCNEHQEHEVEKVRSGRQTGMKWIDRQRERNSGIGNDGKFSKVPGGDKPTKKTDLKYRCGECGKAHLREGWRAGRLEFQE

11110011111110111000000011111111111111101111111111111110111111111111010000111111111111100010

>1JJ2_A

GRRIQGQRRGRGTSTFRAPSHRYKADLEHRKVEDGDVIAGTVVDIEHDPARSAPVAAVEFEDGDRRLILAPEGVGVGDELQVGVDAEIAPGNTLPLAEIPEGVPVCNVESSPGDGGKFARASGVNAQLLTHDRNVAVVKLPSGEMKRLDPQCRATIGVVGGGGRTDKPFVKAGNKHHKMKARGTKWPNVRGVAMNAVDHPFGGGGRQHPGKPKSISRNAPPGRKVGDIASKRTGRGG

111111111111111111111111111000111100000000000011111110000000000000001000000000000000000000000000000000000000011111111111111100000000000000011111010010000000000011111111111111111111111111111111111111111111111111111111111111111111111111111

>1JJ2_B

PQPSRPRKGSLGFGPRKRSTSETPRFNSWPSDDGQPGVQGFAGYKAGMTHVVLVNDEPNSPREGMEETVPVTVIETPPMRAVALRAYEDTPYGQRPLTEVWTDEFHSELDRTLDVPEDHDPDAAEEQIRDAHEAGDLGDLRLITHTVPDAVPSVPKKKPDVMETRVGGGSVSDRLDHALDIVEDGGEHAMNDIFRAGEYADVAGVTKGKGTQGPVKRWGVQKRKGKHARQGWRRRIGNLGPWNPSRVRSTVPQQGQTGYHQRTELNKRLIDIGEGDEPTVDGGFVNYGEVDGPYTLVKGSVPGPDKRLVRFRPAVRPNDQPRLDPEVRYVSNESNQG

1111111111111111111111111111100000000000000110111100000010111000000111110000000000001010000111111110100000010111111111110000001000000000000010100000100111111111111100000000000000000000000000000000000010101111111111111111111111111111111111111111111111111111111111111111000000000000111111111000000001111111111101010011111000000000001011111

>1JJ2_C

MQATIYDLDGNTDGEVDLPDVFETPVRSDLIGKAVRAAQANRKQDYGSDEYAGLRTPAESFGSGRGQAHVPKLDGRARRVPQAVKGRSAHPPKTEKDRSLDLNDKERQLAVRSALAATADADLVADRGHEFDRDEVPVVVSDDFEDLVKTQEVVSLLEALDVHADIDRADETKIKAGQGSARGRKYRRPASILFVTSDEPSTAARNLAGADVATASEVNTEDLAPGGAPGRLTVFTESALAEVAER

000000000000000000000000001011011111111111111111111111111101111111111111111111111111111111111111111111111111101000000000000001111000000000000000000111100100000000100001110111111111111111111110000000010110111110011111111111001111000000000000000001

>1JJ2_D

FHEMREPRIEKVVVHMGIGHANAEDILGEITGQMPVRTKAKRTVGEFDIREGDPIGAKVTLRDEMAEEFLQTALPLAELATSQFDDTGNFSFGLDVTVNLVRPGYRVAKRDKASRSIPTKHRLNPADAVAFIESTYDVEV

00000000001011111110000000000000000011111101110100100011110100000000000000000010011111101111111111100000100010100001100001000000000000000000

>1JJ2_E

PRVELEIPEDVDAEQDHLDITVEGDNGSVTRRLWYPDIDVSVDGDTVVIESDEDNAKTMSTIGTFQSHIENMFHGVTEGWEYGMEVFYSHFPMQVNVEGDEVVIENFLGEKAPRRTTIHGDTDVEIDGEELTVSGPDIEAVGQTAADIEQLTRINDKDVRVFQDGVYITRKP

0000000000000000000000000000001111110000000000000000011110111111101100100000000101000000111000000000000000111111110000100000000000000000011011100110010010111111110000000011

>1JJ2_H

KPGAMYRNSSKPAYTRREYISGIPGKKIAQFDMGNNGAGPTYPAQVELVVEKPVQIRHNALEAARVAANRYVQNSGAAANYKFRIRKFPFHVIRENKDGMRAPFGKPVGTAARVHGANHIFIAWVNPDPNVEEAWRRAKMKVTPTINIDSSPAGNA

111111100111111111110000011000110001100000000000000100001011101100000100000000000001111101000111111111111100000010000110000000000000100110111111111000000001

>1JJ2_I

AEFDADVIVDARDCIMGRVASQVAEQALDGETVAVVNAERAVITGREEQIVEKYEKRVDIGNDNGYFYPKRPDGIFKRTIRGMLPHKKQRGREAFESVRVYLGNPYDEDGEVLDGTSLDRLSNIKFVTLGEISETLGANKTW

1000000000000011111111011001000000000000011111001100111111111111111111101110111111111111110100110111100000000000011001100111100000000111111000

>1JJ2_J

MEALGADVTQGLEKGSLITCADNTGARELKVISVHGYSGTKNRHPKAGLGDKITVSVTKGTPEMRRQVLEAVVVRQRKPIRRPDGTRVKFEDNAAVIVDENEDPRGTELKGPIAREVAQRFGSVASAATMIV

111101111111111111000000000001111111111111111100000001011100000001110100000011101000111010001000000000000000000000000001000000000000

>1JJ2_K

TSKKKRQRGSRTHGGGSHKNRRGAGHRGGRGDAGRDKHEFHNHEPLGKSGFKRPQKVQEEAATIDVREIDENVTLLAADDVAEFRVDVRDVVEEADDADYVKVLGAGQVRHELTLIADDFSEGAREKVEGAGGSVELTDLGEERQ

1111111111111111111111111111111111111111111011111111111111101010111101100000000000000000000011001011011111111100000000111111001000000000000000010

>1JJ2_L

ARSAYSYIREAWKRPKEGQIAELMWHRMQEWRNEPAVVRIERPTRLDRARSLGYKAKQGIIVVRVAIRKGSSRRTRFNKGRRSKRMMVNRITRKKNIQRIAEERANRKFPNLRVLNSYSVGEDGRHKWHEVILIDPDHPAIKSDDQLSWISRTRHRLRTFRGLTSAGRRCRGLRGQGKGSEKVRPSLRVNGAKA

11111100110111110000000010011101111110100110111101111111111110000111111111111111111111111111111111110011001111110011111001111010000000111111111111001001111111111111111111111111111111111111111111

>1JJ2_M

ATGPRYKVPMRRRREARTDYHQRLRLLKSGKPRLVARKSNKHVRAQLVTLGPNGDDTLASAHSSDLAEYGWEAPTGNMPSAYLTGLLAGLRAQEAGVEEAVLDIGLNSPTPGSKVFAIQEGAIDAGLDIPHNDDVLADWQRTRGAHIAEYDEQLEEPLYSGDFDAADLPEHFDELRETLLDGDIEL

111111111111011011110010000000001010111111110101011111111111010110010000011111100000000000000000000000101111111111100000000000000000000010001000011011001000011111000000000000000000000010

>1JJ2_N

SKTNPRLSSLIADLKSAARSSGGAVWGDVAERLEKPRRTHAEVNLGRIERYAQEDETVVVPGKVLGSGVLQKDVTVAAVDFSGTAETKIDQVGEAVSLEQAIENNPEGSHVRVIR

1111101100111011001101111001000111111111010111100110100100001011111110000000000111111000000000000000000000111111111

>1JJ2_O

TDLSAQKRLAADVLDVGKNRVWFNPERQGDIADAITREDVRELVDEGAIQAKDKKGNSRGRARERQKKRAKGHQKGAGSRKGKAGARQNSKEDWESRIRAQRTKLRELRDEGTLSSSQYRDLYDKAGGGEFDSVADLERYIDA

11111011101100011111111111011000001111001000000000011111111111111111111111111111111111111111111111111100010010000001111110111011110111110010000

>1JJ2_P

PSSNGPLEGTRGKLKNKPRDRGTSPPQRAVEEFDDGEKVHLKIDPSVPNGRFHPRFDGQTGTVEGKQGDAYKVDIVDGGKEKTIIVTAAHLRRQE

11111111111111111111111000000000000001011100111111111111111110000011110100011111111111100000111

>1JJ2_Q

GISYSVEADPDTTAKAMLRERQMSFKHSKAIAREIKGKTAGEAVDYLEAVIEGDQPVPFKQHNSGVGHKSKVDGWDAGRYPEKASKAFLDLLENAVGNADHQGFDGEAMTIKHVAAHKVGEQQGRKPRAMGRASAWNSPQVDVELILEEP

111111000000001111101111111010001001000000000000000000000011111111111111111111111111000001101100111011000000000011111111111111111111111111100001000000

>1JJ2_R

SWDVIKHPHVTEKAMNDMDFQNKLQFAVDDRASKGEVADAVEEQYDVTVEQVNTQNTMDGEKKAVVRLSEDDDAQEVASRI

111001001111110010001010100011111110000000000000001111111111011010100000000000000

>1JJ2_T

RECDYCGTDIEPGTGTMFVHKDGATTHFCSSKCENNADLGREARNLEWTDTAR

00000000000011110000000000001111111100111000011111001

>1JJ2_V

MHALVQLRGEVNMHTDIQDTLEMLNIHHVNHCTLVPETDAYRGMVAKVNDFVAFGEPSQETLETVLATRAEPLEGDADVDDEWVAEHTDYDDISGLAFALLSEETTLREQGLSPTLRLHPPRGGHDGVKHPVKEGGQLGKHDTEGIDDLLEAMR

0000001111111111111101111111111000000011111100100000000000000000000110111111000000000000000000000000000000010000111011111111111110000000000000000000000001

>1JJ2_X

TELQARGLTEKTPDLSDEDARLLTQRHRVGKPQFNRQDHHKKKRVSTSWRKPRGQLSKQRRGIKGKGDTVEAGFRSPTAVRGKHPSGFEEVRVHNVDDLEGVDGDTEAVRIASKVGARKRERIEEEAEDAGIRVLNPTYVEV

0010110010000000010111011111111111111111111111111111111111111111111111111111111111111100010101101100000000000111111111111100000000001011101000

>1JJ2_Z

TGAGTPSQGKKNTTTHTKCRRCGEKSYHTKKKVCSSCGFGKSAKRRDYEWQSKAGE

11111111111111111111111111111111111101111111111111111111

>1KNZ_A

TQQMAVSIINSSFEAAVVAATSALENMGIEYDYQDIYSRVKNKFDFVMDDSGVKNNPIGKAITIDQALNNKFGSAIRNRNWLADTSRPAKLDEDVNKLRMMLGIDQKMRVLNACFSVKRIPGKSSSIIKCTKLMRDKLERGEVEVDDSFVDEKM

0000000000000000000000000000000000000000000000001111001111111110010000001111111011000010011011101100100000000000000000101111111000000000000000000000000000

>1LNG_A

MIIWPSYIDKKKSRREGRKVPEELAIEKPSLKDIEKALKKLGLEPKIYRDKRYPRQHWEICGCVEVDYKGNKLQLLKEICKIIKGKN

111100100001111101111110000000000000000000000000011111111100000001111111110010000000000

>1M8V_M

ERPLDVIHRSLDKDVLVILKKGFEFRGRLIGYDIHLNVVLADAEMIQDGEVVKRYGKIVIRGDNVLAISPT

01111101101000000000000000000111110000000000000000000000000000000000000

>1MJI_A

LDVALKRKYYEEVRPELIRRFGYQNVWEVPRLEKVVINQGLGEAKEDARILEKAAQELALITGQKPAVTRAKKSISNFKLRKGMPIGLRVTLRRDRWIFLEKLLNVALPRIRDFRGLNPNSFDGRGNYNLGLREQLIFPEITYDMVDALRGDIAVVTTAETDEEARALLELLGFPFRK

0000000000000000000000000000000011011101001000000000000000000000000011011000000000000100101000000000000000000000000001011111101111111000000000000000011111110000000000000000000000

>1MZP_A

MLADKESLIEALKLALSTEYNVKRNFTQSVEIILTFKGIDKKGDLKLREIVPLPKQPSKAKRVLVVPSSEQLEYAKKASPKVVITREELQKLQGQKRPVKKLARQNEWFLINQESALAGRILGPALGPRGKFPTPLPNTADISEYINRFKRSVLVKTKDQPQVQVFIGTEDKPEDLAENAIAVLNAIENKAKVETNLRNIYVKTTGKAVKVKR

000000000000000000000011111111111111000000000000000000000010110000000000000000000000000000000001100110011010000000000000000110011100000000000000000000000001011111011100000000000000000000000000000001001011111100000

>1N78_B

MVVTRIAPSPTGDPHVGTAYIALFNYAWARRNGGRFIVRIEDTDRARYVPGAEERILAALKWLGLSYDEGPDVGGPHGPYRQSERLPLYQKYAEELLKRGWAYRAFETPEELEQIRKEKGGYDGRARNIPPEEAEERARRGEPHVIRLKVPRPGTTEVKDELRGVVVYDNQEIPDVVLLKSDGYPTYHLANVVDDHLMGVTDVIRAEEWLVSTPIHVLLYRAFGWEAPRFYHMPLLRNPDKTKISKRKSHTSLDWYKAEGFLPEALRNYLCLMGFSMPDGREIFTLEEFIQAFTWERVSLGGPVFDLEKLRWMNGKYIREVLSLEEVAERVKPFLREAGLSWESEAYLRRAVELMRPRFDTLKEFPEKARYLFTEDYPVSEKAQRKLEEGLPLLKELYPRLRAQEEWTEAALEALLRGFAAEKGVKLGQVAQPLRAALTGSLETPGLFEILALLGKERALRRLERALA

000000101000000000000000000000000000000010110010000000000000000000000000000000000100000000000000000000001111100100010001110000000000000000000001101000000000000001101111100101001011110011110000000000000000001111110000000000000000000001101001111000000000000000000000000010011111110101000000000000011111111111011101000100110000000000000000000000000000000000001110000000010000000000000000000000000000000000000000000010001000000000111011001000000111111110000000000000000000

>1QF6_A

PVITLPDGSQRHYDHAVSPMDVALDIGPGLAKACIAGRVNGELVDACDLIENDAQLSIITAKDEEGLEIIRHSCAHLLGHAIKQLWPHTKMAIGPVIDNGFYYDVDLDRTLTQEDVEALEKRMHELAEKNYDVIKKKVSWHEARETFANRGESYKVSILDENIAHDDKPGLYFHEEYVDMCRGPHVPNMRFCHHFKLMKTAGAYWRGDSNNKMLQRIYGTAWADKKALNAYLQRLEEAAKRDHRKIGKQLDLYHMQEEAPGMVFWHNDGWTIFRELEVFVRSKLKEYQYQEVKGPFMMDRVLWEKTGHWDNYKDAMFTTSSENREYCIKPMNCPGHVQIFNQGLKSYRDLPLRMAEFGSCHRNEPSGSLHGLMRVRGFTQDDAHIFCTEEQIRDEVNGCIRLVYDMYSTFGFEKIVVKLSTRPEKRIGSDEMWDRAEADLAVALEENNIPFEYQLGEGAFYGPKIEFTLYDCLDRAWQCGTVQLDFSLPSRLSASYVGEDNERKVPVMIHRAILGSMERFIGILTEEFAGFFPTWLAPVQVVIMNITDSQSEYVNELTQKLSNAGIRVKADLRNEKIGFKIREHTLRRVPYMLVCGDKEVESGKVAVRTRRGKDLGSMDVNEVIEKLQQEIRSRSLKQLEE

00000000000000000000000000000000000000000000000000000000000000000000000000000000000000000000000000000000000001100000000000000000000000000000000000000000000000000000000000000000000000000000000000000011111100111000111101000000000000000000000001011001000000111001100000000000000000000000000000000000000000100001000100110000000000000110100000000000000000000000000011111111111001010000000000000000000000000000000000000000000000000000000000000000000000000000000001111001010000001010010000100000000000000000000000000000000000100000000000000000000000001111100000000000000000000000011111101100000000000111111000000111000001000000000000000000000000000

>1QU2_A

MDYEKTLLMPKTDFPMRGGLPNKEPQIQEKWDAEDQYHKALEKNKGNETFILHDGPPYANGNLHMGHALNKILKDFIVRYKTMQGFYAPYVPGWDTHGLPIEQALTKKGVDRKKMSTAEFREKCKEFALEQIELQKKDFRRLGVRGDFNDPYITLKPEYEAAQIRIFGEMADKGLIYKGKKPVYWSPSSESSLAEAEIEYHDKRSASIYVAFNVKDDKGVVDADAKFIIWTTTPWTIPSNVAITVHPELKYGQYNVNGEKYIIAEALSDAVAEALDWDKASIKLEKEYTGKELEWVVAQHPFLDRESLVINGDHVTTDAGTGCVHTAPGHGEDDYIVGQQYELPVISPIDDKGVFTEEGGQFEGMFYDKANKAVTDLLTEKGALLKLDFITHSYPHDWRTKKPVIFRATPQWFASISKVRQDILDAIENTNFKVNWGKTRIYNMVRDRGEWVISRQRVWGVPLPVFYAENGEIIMTKETVNHVADLFAEHGSNIWFEREAKDLLPEGFTHPGSPNGTFTKETDIMDVWFDSGSSHRGVLETRPELSFPADMYLEGSDQYRGWFNSSITTSVATRGVSPYKFLLSHGFVMDGEGKKMSKSLGNVIVPDQVVKQKGADIARLWVSSTDYLADVRISDEILKQTSDDYRKIRNTLRFMLGNINDFNPDTDSIPESELLEVDRYLLNRLREFTASTINNYENFDYLNIYQEVQNFINVELSNFYLDYGKDILYIEQRDSHIRRSMQTVLYQILVDMTKLLAPILVHTAEEVWSHTPHVKEESVHLADMPKVVEVDQALLDKWRTFMNLRDDVNRALETARNEKVIGKSLEAKVTIASNDKFNASEFLTSFDALHQLFIVSQVKVVDKLDDQATAYEHGDIVIEHADGEKCERCWNYSEDLGAVDELTHLCPRCQQVVKSLV

01110010110000011000000000000000000000000000000000000000000000000000000000000000000000000000000000000000000000000000000000000000000000000000000000000000000000000000000000000000000000010000000000100000000000000000000000000000000000000000000000000000000000000000000000000000000000000000000000000000000000000000000001100000000000000001100100000000000000000000000000000000000000000000000000000000000000000000000000000000000000000000000000011011001100100000000000000000000000000000000000000000000000000000000000000000000000000000000000000000000000000000000000011101000000000000000000000000001010111110000000000000000000000000000111111111000110110011011001101100000000000000000000000000000000000000000000000110110011001101111011001000110000000000000000000000000000000000000000000000000000000000000000000000000011001101100110101111010000000000000000000000000000000000000000000000000000000000101111000000000000000000000000000

>1URN_C

AVPETRPNHTIYINNLNEKIKKDELKKSLHAIFSRFGQILDILVSRSLKMRGQAFVIFKEVSSATNALRSMQGFPFYDKPMRIQYAKTDSDIIAKM

000100010101011101101000000000000000000000101111111110101000000000000000000000100101111111110000

>2FMT_B

SESLRIIFAGTPDFAARHLDALLSSGHNVVGVFTQPDRPAGRGKKLMPSPVKVLAEEKGLPVFQPVSLRPQENQQLVAELQADVMVVVAYGLILPKAVLEMPRLGCINVHGSLLPRWRGAAPIQRSLWAGDAETGVTIMQMDVGLDTGDMLYKLSCPITAEDTSGTLYDKLAELGPQGLITTLKQLADGTAKPEVQDETLVTYAEKLSKEEARIDWSLSAAQLERCIRAFNPWPMSWLEIEGQPVKVWKASVIDTATNAAPGTILEANKQGIQVATGDGILNLLSLQPAGKKAMSAQDLLNSRREWFVPGNRLV

00000000011100000000000000000000001101111111100011000000000000000000000000000000000000001111000000000000000000000000001110010000000000000000000000000000000000000000000100000000000000000000000000000000000011111000000000000000000000001100000000010101000000000000000000000000000000000000001111110100111111110000000000

>1JJ2_S

SKQPDKQRKSQRRAPLHERHKQVRATLSADLREEYGQRNVRVNAGDTVEVLRGDFAGEEGEVINVDLDKAVIHVEDVTLEKTDGEEVPRPLDTSNVRVTDLDLEDEKREARLESEDDSA

11111111101110111101111111100001000111101111100010111111000000011111100000000011111100111111111110000001111100100011111

>1JJ2_U

TVLHVQEIRDMTPAEREAELDDLKTELLNARAVQAAGGAPENPGRIKELRKAIARIKTIQGEEGD

11011110100000000000000100000010010000001111111101101111111011000

>1JJ2_W

ERVVTIPLRDARAEPNHKRADKAMILIREHLAKHFSVDEDAVRLDPSINEAAWARGRANTPSKIRVRAARFEEEGEAIVEAE

0101111011111111111111011001110011110000011111101110111111110110000000000000000000

>1JJ2_Y

RTGRFGPRYGLKIRVRVADVEIKHKKKHKCPVCGFKKLKRAGTGIWMCGHCGYKIAGGCYQPETVAGKAVMKA

1111111111111111000011011010000111001011111010001110000000110001010000000

>1K8W_A

MDINGVLLLDKPQGMSSNDALQKVKRIYNANRAGHTGALDPLATGMLPICLGEATKFSQYLLDSDKRYRVIARLGQRTDTSDADGQIVEERPVTFSAEQLAAALDTFRGDIEQIPSMYSALKYQGKKLYEYARQGIEVPREARPITVYELLFIRHEGNELELEIHCSKGTYIRTIIDDLGEKLGCGAHVIYLRRLAVSKYPVERMVTLEHLRELVEQAEQQDIPAAELLDPLLMPMDSPASDYPVVNLPLTSSVYFKNGNPVRTSGAPLEGLVRVTEGENGKFIGMGEIDDEGRVAPRRLVVE

000000000000000111101100100000111111111110000000010000111110111001000000000000011101100000000000000000000000000000000111111111101100100000000010000000000000000000000001111010000000000000000000010000000000000000000000000000000000000000000000000000000000000000000000000000000000000000000000000000000010000

>1KQ2_M

NIQDKALENFKANQTEVTVFFLNGFQMKGVIEEYDKYVVSLNSQGKQHLIYKHAISTYTVE

1011001000000000000001000000000000111000000000000011110000000

>1M5O_F

PETRPNHTIYINNLNEKIKKDELKKSLHAIFSRFGQILDILVSRSLKMRGQAFVIFKEVSSATNALRSMQGFPFYDKPMRIQYAKTDSDIIAKMK

10100101010111011010000000000000000000001011111111101010000000000000000000001001011111111100000

>1M8Y_B

GRSRLLEDFRNNRYPNLQLREIAGHIMEFSQDQHGSRFIQLKLERATPAERQLVFNEILQAAYQLMVDVFGNYVIQKFFEFGSLEQKLALAERIRGHVLSLALQMYGCRVIQKALEFIPSDQQNEMVRELDGHVLKCVKDQNGNHVVQKCIECVQPQSLQFIIDAFKGQVFALSTHPYGCRVIQRILEHCLPDQTLPILEELHQHTEQLVQDQYGNYVIQHVLEHGRPEDKSKIVAEIRGNVLVLSQHKFASNVVEKCVTHASRTERAVLIDEVCTMNDGPHSALYTMMKDQYANYVVQKMIDVAEPGQRKIVMHKIRPHIATLRKYTYGKHILAKLEKYYM

000000000000000000000000000000011001100100000000000000000000000000011101100110000000000000000000000000001101100100000000000000000000000000001101110100000000000000000000000000001101100110000000000000000000000000011101110100000000000000000000000000011101100110000000000000000000000000000000001110110010000000000000000000000000000111011000000000

>1MMS_A

QIKLQLPAGKATPAPPVGPALGQHGVNIMEFCKRFNAETADKAGMILPVVITVYEDKSFTFIIKTPPASFLLKKAAGIEKGSSEPKRKIVGKVTRKQIEEIAKTKMPDLNANSLEAAMKIIEGTAKSMGIEVV

0011100000000000000000110000000000000000000000010000000000000001111111001000001111111110000000000000000010011111101111111111111110000

>1N35_A

SSMILTQFGPFIESISGITDQSNDVFEDAAKAFSMFTRSDVYKALDEIPFSDDAMLPIPPTIYTKPSHDSYYYIDALNRVRRKTYQGPDDVYVPNCSIVELLEPHETLTSYGRLSEAIENRAKDGDSQARIATTYGRIAESQARQIKAPLEKFVLALLVAEAGGSLYDPVLQKYDEIPDLSHNCPLWCFREICRHISGPLPDRAPYLYLSAGVFWLMSPRMTSAIPPLLSDLVNLAILQQTAGLDPSLVKLGVQICLHAAASSSYSWFILKTKSIFPQNTLHSMYESLEGGYCPNLEWLEPRSDYKFMYMGVMPLSAKYARSAPSNDKKARELGEKYGLSSVVGELRKRTKTYVKHDFASVRYIRDAMACTSGIFLVRTPTETVLQEYTQSPEIKVPIPQKDWTGPIGEIRILKDTTSSIARYLYRTWYLAAARMAAQPRTWDPLFQAIMRSQYVTARGGSGAALRESLYAINVSLPDFKGLPVKAATKIFQAAQLANLPFSHTSVAILADTSMGLRNQVQRRPRSIMPLNVPQQQVSAPHTLTADYINYHMNLSPTSGSAVIEKVIPLGVYASSPPNQSINIDISACDASITWDFFLSVIMAAIHEGVASSSIGKPFMGVPASIVNDESVVGVRAARPISGMQNMIQHLSKLYKRGFSYRVNDSFSPGNDFTHMTTTFPSGSTATSTEHTANNSTMMETFLTVWGPEHTDDPDVLRLMKSLTIQRNYVCQGDDGLMIIDGTTAGKVNSETIQNDLELISKYGEEFGWKYDIAYDGTAEYLKLYFIFGCRIPNLSRHPIVGKERANSSAEEPWPAILDQIMGVFFNGVHDGLQWQRWIRYSWALCCAFSRQRTMIGESVGYLQYPMWSFVYWGLPLVKAFGSDPWIFSWYMPTGDLGMYSWISLIRPLMTRWMVANGYVTDRCSTVFGNADYRRCFNELKLYQGYYMAQLPRNPKKSGRAASREVREQFTQALSDYLMQNPELKSRVLRGRSEWEKYGAGIIHNPPSLFDVPHKWYQGAQEAAIATREELAEMDETLMRARRHSYSSFSKLLEAYLLVKWRMCEAREPSVDLRLPLCAGIDPLNSDPFLKMVSVGPMLQSTRKYFAQTLFMAKTVSGLDVNAIDSALLRLRTLGADKKALTAQLLMVGLQESEADALAGKIMLQDVNTVQLARVVNLAVPDTWMSLDFDSMFKHHVKLLPKDGRHLNTDIPPRMGWLRAILRFLGAGMVMTATGVAVDIYLEDIHGGGRSLGQRFMTWMRQEGR

0000000000000000000000000000000000000000000000000000000000000000000000000000000000000000000000000000000000000000000000000000000000000000000000000000000000000000000000000000000000000000000000000000000000000000000000000000000000000000000000000000000000000000000000000000000000000000000000000000000000000000000000000000000000000000000000000000000000000000000000000000000000000000000000000000000000000000000000000000000000000000000000000000000000000000000011011111111000000000000000000000101010000000000000000000000011111000000010111100001000000000000000000000111110110000000000000000000000000000000000000000000000000000000000000000000000000000000000000000000000000010000000000000000011111110000000000000000000000000000000000000000000000000000000000000000000000000000000000000000000000000000000000000000000000101000000000000000000000000000000000000000000000000000000000000000000000000000000000000100000000000000000000000000000000000000000000000000000000000000000000000000000000000000000000000000000000000000000000000000000000001000000000000000000000000000000000000000000000000000000000000000000000000000011001000000010000000100000000000000000000000000000000000000000000000000010000111100100000000000000000000000000000000000000000000000000000000000000000000000000000000

>1NB7_B

SMSYTWTGALITPCAAEESKLPINPLSNSLLRHHNMVYATTSRSASLRQKKVTFDRLQVLDDHYRDVLKEMKAKASTVKAKLLSIEEACKLTPPHSAKSKFGYGAKDVRNLSSRAVNHIRSVWEDLLEDTETPIDTTIMAKSEVFCVQPEKGGRKPARLIVFPDLGVRVCEKMALYDVVSTLPQAVMGSSYGFQYSPKQRVEFLVNTWKSKKCPMGFSYDTRCFDSTVTESDIRVEESIYQCCDLAPEARQAIRSLTERLYIGGPLTNSKGQNCGYRRCRASGVLTTSCGNTLTCYLKATAACRAAKLQDCTMLVNGDDLVVICESAGTQEDAAALRAFTEAMTRYSAPPGDPPQPEYDLELITSCSSNVSVAHDASGKRVYYLTRDPTTPLARAAWETARHTPINSWLGNIIMYAPTLWARMILMTHFFSILLAQEQLEKALDCQIYGACYSIEPLDLPQIIERLHGLSAFTLHSYSPGEINRVASCLRKLGVPPLRTWRHRARSVRAKLLSQGGRAATCGRYLFNWAVRTKLKLTPIPAASQLDLSGWFVAGYSGGDIYHSLS

0000000000000100000000000000000000000000000000000000000000000000000000000000000000000000000011111100000000000000000000000000000000000000001010000000000000000111111000010000000000000000000000000000000000000000000000000000000010000000000000000000000000000000000000000000000000000001111100111011000000000000000000000000110000000000000000000000000000000000000000000000000000000000000000000000000000000000000110000000000000000000000000000000000000010101111000000000000000000000000000000000000000000000000000000000000000000000000000000000000000000000000000000011111110000

>1QTQ_A

TNFIRQIIDEDLASGKHTTVHTRFPPEPNGYLHIGHAKSICLNFGIAQDYKGQCNLRFDDTNPVKEDIEYVESIKNDVEWLGFHWSGNVRYSSDYFDQLHAYAIELINKGLAYVDELTPEQIREYRGTLTQPGKNSPYRDRSVEENLALFEKMRAGGFEEGKACLRAKIDMASPFIVMRDPVLYRIKFAEHHQTGNKWCIYPMYDFTHCISDALEGITHSLCTLEFQDNRRLYDWVLDNITIPVHPRQYEFSRLNLEYTVMSKRKLNLLVTDKHVEGWDDPRMPTISGLRRRGYTAASIREFCKRIGVTKQDNTIEMASLESCIREDLNENAPRAMAVIDPVKLVIENYQGEGEMVTMPNHPNKPEMGSRQVPFSGEIWIDRADFREEANKQYKRLVLGKEVRLRNAYVIKAERVEKDAEGNITTIFCTYDADTLGVIHWVSAAHALPVEIRLYDRLFSVPNPGAADDFLSVINPESLVIKQGFAEPSLKDAVAGKAFQFEREGYFCLDSRHSTAEKPVFNRTVGLRDT

1110010000000000000000001110000000000000000000000000000000111100110000000000000000000000000000000000000000000000001111100110011111000000000000000000000000000001111100000000011110000101111000000000000001111001000000000000000111111110000000000000000000010000100000100000000000000000000000000000000000000000111111111111011101001000100001000000000000000000000000001110000000000000000000000100000111100000001111110000000000000000000000000111110000000000000000000000000000000000000000000000000000000000001111101000000000000000000001111

>1SER_B

MVDLKRLRQEPEVFHRAIREKGVALDLEALLALDREVQELKKRLQEVQTERNQVAKRVPKAPPEEKEALIARGKALGEEAKRLEEALREKEARLEALLLQVPLPPWPGAPVGGEEANREIKRVGGPPEFSFPPLDHVALMEKNGWWEPRISQVSGSRSYALKGDLALYELALLRFAMDFMARRGFLPMTLPSYAREKAFLGTGHFPAYRDQVWAIAETDLYLTGTAEVVLNALHSGEILPYEALPLRYAGYAPAFRSEAGSFGKDVRGLMRVHQFHKVEQYVLTEASLEASDRAFQELLENAEEILRLLELPYRLVEVATGDMGPGKWRQVDIEVYLPSEGRYRETHSCSALLDWQARRANLRYRDPEGRVRYAYTLNNTALATPRILAMLLENHQLQDGRVRVPQALIPYMGKEVLEPCG

0000100100000000000000000000000000000100110110011011111111100000010011111100100010011001000000000000000000000000000000000000000000000000000000000000000000000000000000000000000000000000000000000010000000000000000000000010000000000000000001000000000000000000000000000000000000000000000000000000000000000000000000000000000000000000000000000000000000000000000000000010100011100000000000000000000000000000000000000000000000000

>2A8V_B

MNLTELKNTPVSELITLGENMGLENLARMRKQDIIFAILKQHAKSGEDIFGDGVLEILQDGFGFLRSADSSYLAGPDDIYVSPSQIRRFNLRTGDTISGKIRPPKEGERYFALLKVNE

0000000000000000000000000000000000000000000000000000000101110101010000000111010100100000000000000000010110111111000000
