# Supplementary material for: Predicting RNA-binding sites of proteins using support vector machines and evolutionary information
Source: BMC Bioinformatics. 2008 Dec 12;9(Suppl 12):S6. doi: 10.1186/1471-2105-9-S12-S6 (PMC2638146; doi:10.1186/1471-2105-9-S12-S6)
Supplement: Additional file 2 — The RBP109 data set. [file 1471-2105-9-S12-S6-S2.doc]

# The RBP109 dataset

>1A34_A

MGRGKVKPNRKSTGDNSNVVTMIRAGSYPKVNPTPTWVRAIPFEVSVQSGIAFKVPVGSLFSANFRTDSFTSVTVMSVRAWTQLTPPVNEYSFVRLKPLFKTGDSTEEFEGRASNINTRASVGYRIPTNLRQNTVAADNVCEVRSNCRQVALVISCCFN

000000000000110000000010000000000011011100000000000000000000000000000000000000100000000000000000000000000000000000000000000000000000000000000000000000000010000

>1A9N_A

MVKLTAELIEQAAQYTNAVRDRELDLRGYKIPVIENLGATLDQFDAIDFSDNEIRKLDGFPLLRRLKTLLVNNNRICRIGEGLDQALPDLTELILTNNSLVELGDLDPLASLKSLTYLCILRNPVTNKKHYRLYVIYKVPQVRVLDFQKVKLKERQEAEKMFKGKRGAQLAKDIAR

00000000000000000000000000000000000000000000000000000000000000000000000000000000000000000000000000000000000000000000000000000000000000000000000000001000100000000000000000000000

>1ASY_A

EDTAKDNYGKLPLIQSRDSDRTGQKRVKFVDLDEAKDSDKEVLFRARVHNTRQQGATLAFLTLRQQASLIQGLVKANKEGTISKNMVKWAGSLNLESIVLVRGIVKKVDEPIKSATVQNLEIHITKIYTISETPEALPILLEDASRSEAEAEAAGLPVVNLDTRLDYRVIDLRTVTNQAIFRIQAGVCELFREYLATKKFTEVHTPKLLGAPSEGGSSVFEVTYFKGKAYLAQSPQFNKQQLIVADFERVYEIGPVFRAENSNTHRHMTEFTGLDMEMAFEEHYHEVLDTLSELFVFIFSELPKRFAHEIELVRKQYPVEEFKLPKDGKMVRLTYKEGIEMLRAAGKEIGDFEDLSTENEKFLGKLVRDKYDTDFYILDKFPLEIRPFYTMPDPANPKYSNSYDFFMRGEEILSGAQRIHDHALLQERMKAHGLSPEDPGLKDYCDGFSYGCPPHAGGGIGLERVVMFYLDLKNIRRASLFPRDPKRLRP

0000000000000000000000000000000000000000000000000101111001010000000000101010000000000001000000000000000000000111110000001000000000000011011100100000000001011111011000000000000000000000000000000000000000000000000000000000000001000000000000000000000000000000010111110111001000000000000000000000000000000000000000000000000000000000000000000000000000000000000010000000000000000000000000000100000000000000000000000011110000000000000000000000000000000000000000000011100100000000001110000000011000

>1AV6_A

VVSLDKPFMYFEEIDNELDYEPESANEVAKKLPYQGQLKLLLGELFFLSKLQRHGILDGATVVYIGSAPGTHIRYLRDHFYNLGVIIKWMLIDGRHHDPILNGLRDVTLVTRFVDEEYLRSIKKQLHPSKIILISDVRSPSTADLLSNYALQNVMISILNPVASSLKWRCPFPDQWIKDFYIPHGNKMLQPFAPSYSAEMRLLSIYTGENMRLTRVTKSDAVNYEKKMYYLNKIVRNKVVVNFDYPNQEYDYFHMYFMLRTVYCNKTFPTTKAKVLFLQQSIFRFLNIP

0000000000000000000000000000010011111011000000000000000000000000000010000000000000000000000000000000000000000000000000000000000000000000010100000000000000000000000000100000000000000000000000000100001000000000000000000000000000000000000000000000000000000000000000000000000000000000000000000

>1B23_P

AKGEFIRTKPHVNVGTIGHVDHGKTTLTAALTYVAAAENPNVEVKDYGDIDKAPEERARGITINTAHVEYETAKRHYSHVDCPGHADYIKNMITGAAQMDGAILVVSAADGPMPQTREHILLARQVGVPYIVVFMNKVDMVDDPELLDLVEMEVRDLLNQYEFPGDEVPVIRGSALLALEEMHKNPKTKRGENEWVDKIWELLDAIDEYIPTPVRDVDKPFLMPVEDVFTITGRGTVATGRIERGKVKVGDEVEIVGLAPETRKTVVTGVEMHRKTLQEGIAGDNVGLLLRGVSREEVERGQVLAKPGSITPHTKFEASVYILKKEEGGRHTGFFTGYRPQFYFRTTDVTGVVRLPQGVEMVMPGDNVTFTVELIKPVALEEGLRFAIREGGRTVGAGVVTKILE

000000000000000000000000000000000000000000000000000101000000000000000000000000000000000001000000000000000000000000000000000000000000000000000000000000000000000000000000000000000000000000000000000000000000000000000000000000000000111101001010000000000000000000000000000011101100000000000011100000000000000000000000000000000000000000000000000010000000010000000000000000000000001100000000000001100000000000000

>1COA_A

MRTEYCGQLRLSHVGQQVTLCGWVNRRRDLGSLIFIDMRDREGIVQVFFDPDRADALKLASELRNEFCIQVTGTVRARDEKNINRDMATGEIEVLASSLTIINRADVLPLDSNHVNTEEARLKYRYLDLRRPEMAQRLKTRAKITSLVRRFMDDHGFLDIETPMLTKATPEGARDYLVPSRVHKGKFYALPQSPQLFKQLLMMSGFDRYYQIVKCFRDEDLRADRQPEFTQIDVETSFMTAPQVREVMEALVRHLWLEVKGVDLGDFPVMTFAEAERRYGSDKPDLRNPMELTDVADLLKSVEFAVFAGPANDPKGRVAALRVPGGASLTRKQIDEYGNFVKIYGAKGLAYIKVNERAKGLEGINSPVAKFLNAEIIEDILDRTAAQDGDMIFFGADNKKIVADAMGALRLKVGKDLGLTDESKWAPLWVIDFPMFEDDGEGGLTAMHHPFTSPKDMTAAELKAAPENAVANAYDMVINGYEVGGGSVRIHNGDMQQTVFGILGINEEEQREKFGFLLDALKYGTPPHAGLAFGLDRLTMLLTGTDNIRDVIAFPKTTAAACLMTEAPSFANPTALAELSIQVVK

000000000000000000000000000000000000000000000000000000000000000000000000000000000000000000000000000000000000000000000110000000000000000000000000000000000000000000000000111110000000000000000001101100000000000000000000101101101000000000000000000000000000000000000000000000000000000000000000000000000000000000000000000000000000000000000000000000110000000000000000000000000000000000000000000000000000000100000000000000000000000000000000000000000000000111100000000000000000000000000000000000000000000000000000000000000100000000000000000000000000000000001000000011000010000000000000000000000

>1DDL_A

MEQDKILAHQASLNTKPSLLPPPVGNPPPVISYPFQITLASLGTEDAADSVSIASNSVLATYTALYRHAQLKHLKATIHPTYMAPKYPTSVALVWVPANSTATSTQVLDTYGGLHFCIGGSVNSVKPIDVEANLTNLNPIIKASTTFTDTPKLLYYSKAQATAPTSPTCYLTIQGQIELSSPLLQASS

00000000000000000000000111000000000000000000000000000000000000000000000000000000000000000000111000000000001001111100000000000000011010010000000000000000000000000000000000000000000000000000

>1DFU_P

MFTINAEVRKEQGKGASRRLRAANKFPAIIYGGKEAPLAIELDHDKVMNMQAKAEFYSEVLTIVVDGKEIKVKAQDVQRHPYKPKLQHIDFVRA

0000000010011100111010000010101100001010000000000000000000000000000000000010010000000011010100

>1DI2_A

MPVGSLQELAVQKGWRLPEYTVAQESGPPHKREFTITCRVETFVETGSGTSKQVAKRVAAEKLLTKFKT

001100100000000000000000000011010100000000000000011110011000000000000

>1E6T_A

ASNFTQFVLVDNGGTGDVTVAPSNFANGVAEWISSNSRSQAYKVTCSVRQSSAQNRKYTIKVEVPKVATQTVGGVELPVAAWRSYLNMELTIPIFATNSDCELIVKAMQGLLKDGNPIPSAIAANSGIY

000000000000000000000000000010000000000000101110101000001010101000000000000000000010101000000000000000000000000000000000000000000

>1E7K_A

MTEADVNPKAYPLADAHLTKKLLDLVQQSCNYKQLRKGANEATKTLNRGISEFIVMAADAEPLEIILHLPLLCEDKNVPYVFVRSKQALGRACGVSRPVIACSVTIKEGSQLKQQIQSIQQSIERLLV

00000000000000000000000000000000000111111001000100000000000110001000000000000000000001000000001110110000000000000000000000000000

>1E8O_A

PQYQTWEEFSRAAEKLYLADPMKARVVLKYRHSDGNLCVKVTDDLVCLVYKTDQAQDVKKIEKFHSQLMRLMVAKEARNVTMETE

0000000000000000000000000000000000000000000000000000000000100010011001001000000000000

>1E8O_B

VLLESEQFLTELTRLFQKCRTSGSVYITLKKYDGRTKPIPKKGTVEGFEPADNKCLLRATDGKKKISTVVSSKEVNKFQMAYSNLLRANMDGLKKRDKKNKTKKTK

0000000000000000000000000000000000000000000000000000000000000000000000010011001000000010000000000000000000

>1EC6_A

MKELVEIAVPENLVGAILGKGGKTLVEYQELTGARIQISKKGEFLPGTRNRRVTITGSPAATQAAQYLISQRVTYEQGVRASNPQKV

000000000011011101111100110000000001110110000000101000000000000000000000000000010000100

>1EIY_A

MLEEALAAIQNARDLEELKALKARYLGKKGLLTQEMKGLSALPLEERRKRGQELNAIKAALEAALEAREKALEEAALKEALERERVDVSLPGASLFSGGLHPITLMERELVEIFRALGYQAVEGPEVESEFFNFDALNIPEHHPARDMWDTFWLTGEGFRLEGPLGEEVEGRLLLRTHTSPMQVRYMVAHTPPFRIVVPGRVFRFEQTDATHEAVFHQLEGLVVGEGIAMAHLKGAIYELAQALFGPDSKVRFQPVYFPFVEPGAQFAVWWPEGGKWLELGGAGMVHPKVFQAVDAYRERLGLPPAYRGVTGFAFGLGVERLAMLRYGIPDIRYFFGGRLKFLEQFKGVL

00000000000000000000000000000000000000000000000000000000000000000000000000000000000000000000000000000000000000000000000000000000000000000000000000111000000000000000000000000000010000000000000000000000000100000000000001010000000000000000000000000000000000000101000000000000000000001100000000000000000000000000000000010000000000000000000000000000000000

>1EIY_B

MRVPFSWLKAYVPELESPEVLEERLAGLGFETDRIERVFPIPRGVVFARVLEAHPIPGTRLKRLVLDAGRTVEVVSGAENARKGIGVALALPGTELPGLGQKVGERVIQGVRSFGMALSPRELGVGEYGGGLLEFPEDALPPGTPLSEAWPEEVVLDLEVTPNRPDALGLLGLARDLHALGYALVEPEAALKAEALPLPFALKVEDPEGAPHFTLGYAFGLRVAPSPLWMQRALFAAGMRPINNVVDVTNYVMLERAQPMHAFDLRFVGEGIAVRRAREGERLKTLDGVERTLHPEDLVIAGWRGEESFPLGLAGVMGGAESEVREDTEAIALEVACFDPVSIRKTARRHGLRTEASHRFERGVDPLGQVPAQRRALSLLQALAGARVAEALLEAGSPKPPEAIPFRPEYANRLLGTSYPEAEQIAILKRLGCRVEGEGPTYRVTPPSHRLDLRLEEDLVEEVARIQGYETIPLALPAFFPAPDNRGVEAPYRKEQRLREVLSGLGFQEVYTYSFMDPEDARRFRLDPPRLLLLNPLAPEKAALRTHLFPGLVRVLKENLDLDRPERALLFEVGRVFREREETHLAGLLFGEGVGLPWAKERLSGYFLLKGYLEALFARLGLAFRVEAQAFPFLHPGVSGRVLVEGEEVGFLGALHPEIAQELELPPVHLFELRLPLPDKPLAFQDPSRHPAAFRDLAVVVPAPTPYGEVEALVREAAGPYLESLALFDLYQGPPLPEGHKSLAFHLRFRHPKRTLRDEEVEEAVSRVAEALRARGFGLRGLDTP

11000000000000000000000000000000000000000000000000000000000000000000000000000000000000000000000000000000000000000000000000000000000000000000000000000000000000110000000000000000000000000000000000000000000000000000000000000000000000000000000000000000000000000000000000000000000000000000000000000000000000000000000000000000000000000000000000000000000000000000011001000000000000000000000000000000000000000000000000000000000000000000000000000000000000000000000000000000000000000000000000000000000000000000000000000000000000000000000000000000000000000000000000000000110100000000000000000000000000000000000000000000000000000000000000000000000000000000000000000000000000000000000000000000000000000000000000000000000000000000000000000000000000000000000000000000000000000000000000000000000000000

>1F7U_A

MASTANMISQLKKLSIAEPAVAKDSHPDVNIVDLMRNYISQELSKISGVDSSLIFPALEWTNTMERGDLLIPIPRLRIKGANPKDLAVQWAEKFPCGDFLEKVEANGPFIQFFFNPQFLAKLVIPDILTRKEDYGSCKLVENKKVIIEFSSPNIAKPFHAGHLRSTIIGGFLANLYEKLGWEVIRMNYLGDWGKQFGLLAVGFERYGNEEALVKDPIHHLFDVYVRINKDIEEEGDSIPLEQSTNGKAREYFKRMEDGDEEALKIWKRFREFSIEKYIDTYARLNIKYDVYSGESQVSKESMLKAIDLFKEKGLTHEDKGAVLIDLTKFNKKLGKAIVQKSDGTTLYLTRDVGAAMDRYEKYHFDKMIYVIASQQDLHAAQFFEILKQMGFEWAKDLQHVNFGMVQGMSTRKGTVVFLDNILEETKEKMHEVMKKNENKYAQIEHPEEVADLVGISAVMIQDMQGKRINNYEFKWERMLSFEGDTGPYLQYAHSRLRSVERNASGITQEKWINADFSLLKEPAAKLLIRLLGQYPDVLRNAIKTHEPTTVVTYLFKLTHQVSSCYDVLWVAGQTEELATARLALYGAARQVLYNGMRLLGLTPVERM

0000000000000000000000000000000000000000000000000000000000000000000000000000000000000000000000000000000000000000000000000000000000000000000000000000000010000000000000000000000000000000000000000110000000000000000000000000000000000000000000000000000000000000000000000000000000000000000000000000010000000000000000000000001010100000000000001001100001100100000000000000000000000110110000000000000000000000000101000000000000000000000000000000000000000000000000000000000000000000000000000000000000000000000000000000000000000000000000000000000000000000000000000000000000000000000000000000000000000000000000000000000

>1F8V_A

NRRNKARKVVSRSTALVPMAPASQRTGPAPRKPRKRNQALVRNPRLTDAGLAFLKCAFAAPDFSVDPGKGIPDNFHGRTLAIKDCNTTSVVFTPNTDTYIVVAPVPGFAYFRAEVAVGAQPTTFVGVPYPTYATNFGAGSQNGLPAVNNYSKFRYASMACGLYPTSNMMQFSGSVQVWRVDLNLSEAVNPAVTAITPAPGVFANFVDKRINGLRGIRPLAPRDNYSGNFIDGAYTFAFDKSTDFEWCDFVRSLEFSESNVLGAATAMKLLAPGGGTDTTLTGLGNVNTLVYKISTPTGAVNTAILRTWNCIELQPYTDSALFQFSGVSPPFDPLALECYHNLKMRFPVAVSSREN

0000101100000000000000000000000000100000000000000000000000000000000000000000000000000000000000000000000000000000000000000000000000000000000000000000000000000000000000000000000000000000000000000000000000000000000000000000000000000000000000000000000000000000000000000000000000000000000000000000000000000000000000000000000000000000000000000000000000000000000

>1FEU_A

MEYRLKAYYREGEKPSALRRAGKLPGLMYNRHLNRKVYVDLVEFDKVFRQASIHHVIVLELPDGQSLPTLVRQVNLDKRRRRPEHVDFFVLSDEPVEMYVPLRFVGTPAGVRAGGVLQEIHRDILVKVSPRNIPEFIEVDVSGLEIGDSLHASDLKLPPGVELAVSPEETIAAVVPPEDVEKLAEEAAAEVAEPEVIKKGKEEEEE

00000000010001110011000010101110010100000000000000000000000000000000000110100100000110101000000000000000000000000000000000000000000000000000000000000000000000000000000000000000000000000000000000000000000000

>1FFY_A

MDYEKTLLMPKTDFPMRGGLPNKEPQIQEKWDAEDQYHKALEKNKGNETFILHDGPPYANGNLHMGHALNKILKDFIVRYKTMQGFYAPYVPGWDTHGLPIEQALTKKGVDRKKMSTAEFREKCKEFALEQIELQKKDFRRLGVRGDFNDPYITLKPEYEAAQIRIFGEMADKGLIYKGKKPVYWSPSSESSLAEAEIEYHDKRSASIYVAFNVKDDKGVVDADAKFIIWTTTPWTIPSNVAITVHPELKYGQYNVNGEKYIIAEALSDAVAEALDWDKASIKLEKEYTGKELEWVVAQHPFLDRESLVINGDHVTTDAGTGCVHTAPGHGEDDYIVGQQYELPVISPIDDKGVFTEEGGQFEGMFYDKANKAVTDLLTEKGALLKLDFITHSYPHDWRTKKPVIFRATPQWFASISKVRQDILDAIENTNFKVNWGKTRIYNMVRDRGEWVISRQRVWGVPLPVFYAENGEIIMTKETVNHVADLFAEHGSNIWFEREAKDLLPEGFTHPGSPNGTFTKETDIMDVWFDSGSSHRGVLETRPELSFPADMYLEGSDQYRGWFNSSITTSVATRGVSPYKFLLSHGFVMDGEGKKMSKSLGNVIVPDQVVKQKGADIARLWVSSTDYLADVRISDEILKQTSDDYRKIRNTLRFMLGNINDFNPDTDSIPESELLEVDRYLLNRLREFTASTINNYENFDYLNIYQEVQNFINVELSNFYLDYGKDILYIEQRDSHIRRSMQTVLYQILVDMTKLLAPILVHTAEEVWSHTPHVKEESVHLADMPKVVEVDQALLDKWRTFMNLRDDVNRALETARNEKVIGKSLEAKVTIASNDKFNASEFLTSFDALHQLFIVSQVKVVDKLDDQATAYEHGDIVIEHADGEKCERCWNYSEDLGAVDELTHLCPRCQQVVKSLV

00110010100000011000000000000000000000000000000000000000000000000000000000000000000000000000000000000000000000000000000000000000000000000000000000000000000000000000000000000000000000000000000000000000000000000000000000000000000000000000000000000000000000000000000000000000000000000000000000000000000000000000000000100000000000000000100000000000000000000000000000000000000000000000000000000000000000000000000000000000000000000000000000010011001000000000000000000000000000000000000000000000000000000000000000000000000000000000000000000000000000000000000000011001000000000000000000000000001010001110000000000000000000000000000101001101000110110010001001101100000000000000000000000000000000000000000000000100010011001100110011001000110000000000000000000000000000000000000000000000000000000000000000000000000010001100100100100110000000000000000000000000000000000000000000000000000000000000100101000000000000000000000000000

>1FJG_B

MPVEITVKELLEAGVHFGHERKRWNPKFARYIYAERNGIHIIDLQKTMEELERTFRFIEDLAMRGGTILFVGTKKQAQDIVRMEAERAGMPYVNQRWLGGMLTNFKTISQRVHRLEELEALFASPEIEERPKKEQVRLKHELERLQKYLSGFRLLKRLPDAIFVVDPTKEAIAVREARKLFIPVIALADTDSDPDLVDYIIPGNDDAIRSIQLILSRAVDLIIQARGGVVEPSPSYALVQEAEATETPEGESEVEA

0000000000000000000001010110000000000000000000000000000000000000000000000000000000000000000000010111001100110010010000000000000000011000100000010011000000000000000000000001001100100000000000000000000000000000000000000000000000000000000000000000000000000000

>1FJG_C

MGNKIHPIGFRLGITRDWESRWYAGKKQYRHLLLEDQRIRGLLEKELYSAGLARVDIERAADNVAVTVHVAKPGVVIGRGGERIRVLREELAKLTGKNVALNVQEVQNPNLSAPLVAQRVAEQIERRFAVRRAIKQAVQRVMESGAKGAKVIVSGRIGGAEQARTEWAAQGRVPLHTLRANIDYGFALARTTYGVLGVKAYIFLGEVIGGQKPKARPELPKAEERPRRRRPAVRVKKEE

01111000010001000000000001100000000000000000000000000000000000000000000000000000000000000000000000000000000000000000000000000010000000000000000000000100011100111110001000111101111000000100011111111010000000000000000000000000000000000000000

>1FJG_D

MGRYIGPVCRLCRREGVKLYLKGERCYSPKCAMERRPYPPGQHGQKRARRPSDYAVRLREKQKLRRIYGISERQFRNLFEEASKKKGVTGSVFLGLLESRLDNVVYRLGFAVSRRQARQLVRHGHITVNGRRVDLPSYRVRPGDEIAVAEKSRNLELIRQNLEAMKGRKVGPWLSLDVEGMKGKFLRLPDREDLALPVQENLVIEFYSR

01101011110011000000010110000100001101011110110010010110011011001100000111010000000110000000000000010000000000011011011001101000000111111100100000000010001100000000000000000000000000000000000000000000000010011

>1FJG_E

MPETDFEEKMILIRRTARMQAGGRRFRFGALVVVGDRQGRVGLGFGKAPEVPLAVQKAGYYARRNMVEVPLQNGTIPHEIEVEFGASKIVLKPAAPGTGVIAGAVPRAILELAGVTDILTKELGSRNPINIAYATMEALRQLRTKADVERLRKGEAHAQAQG

000000000000010111111101101000000000000000001010100000001000100000000000000000000001110100010110010011100010000000000011111011101100100000000000000000000000000000

>1FJG_G

MARRRRAEVRQLQPDLVYGDVLVTAFINKIMRDGKKNLAARIFYDACKIIQEKTGQEPLKVFKQAVENVKPRMEVRSRRVGGANYQVPMEVSPRRQQSLALRWLVQAANQRPERRAAVRIAHELMDAAEGKGGAVKKKEDVERMAEANRAYAHYRW

011111100101000000000000000111111111110011000000000000000000000000000000000100101100000000000110010001000000100001010010000000000000000000000000000000000000

>1FJG_I

MEQYYGTGRRKEAVARVFLRPGNGKVTVNGQDFNEYFQGLVRAVAALEPLRAVDALGRFDAYITVRGGGKSGQIDAIKLGIARALVQYNPDYRAKLKPLGFLTRDARVVERKKYGKHKARRAPQYSKR

00101010111101010101000000000000000101110100000000000000000001000011111110000000001000000000100010000111011111111111110111111111

>1FJG_J

MPKIRIKLRGFDHKTLDASAQKIVEAARRSGAQVSGPIPLPTRVRRFTVIRGPFKHKDSREHFELRTHNRLVDIINPNRKTIEQLMTLDLPTGVEIEIKTVGGGR

000010101000110010000000000100000011111111111101011111111011010001010110100000000000000000000000000000000

>1FJG_K

MAKKPSKKKVKRQVASGRAYIHASYNNTIVTITDPDGNPITWSSGGVIGYKGSRKGTPYAAQLAALDAAKKAMAYGMQSVDVIVRGTGAGREQAIRALQASGLQVKSIVDDTPVPHNGCRPKKKFRKAS

000000000001000001010100011010100001111101010110001110100000000000000010000000000000100000000000000000000000000011111111111101000

>1FJG_L

MVALPTINQLVRKGREKVRKKSKVPALKGAPFRRGVCTVVRTVTPKKPNSALRKVAKVRLTSGYEVTAYIPGEGHNLQEHSVVLIRGGRVKDLPGVRYHIVRGVYDAAGVKDRKKSRSKYGTKKPKEAAKTAAKK

000011111101101111111011000111111100000010000110111111001000100000001001100000000001011011110100100010111000000011111110111100000000000

>1FJG_M

MARIAGVEIPRNKRVDVALTYIYGIGKARAKEALEKTGINPATRVKDLTEAEVVRLREYVENTWKLEGELRAEVAANIKRLMDIGCYRGLRHRRGLPVRGQRTRTNARTRKGPRKTVAGKKKAPRK

000000000000110010001111110110000000000000010000000000000000000000000110010011010000001100110101111111111111111001111001000111

>1FJG_N

MARKALIEKAKRTPKFKVRAYTRCVRCGRARSVYRFFGLCRICLRELAHKGQLPGVRKASW

0111110000100011011011100010101111100000111011000000000011011

>1FJG_P

MVKIRLARFGSKHNPHYRIVVTDARRKRDGKYIEKIGYYDPRKTTPDWLKVDVERARYWLSVGAQPTDTARRLLRQAGVFRQEAREGA

1110110111111101110000111111111110100100111110000100000000100110101110110010000111100000

>1FJG_Q

MPKKVLTGVVVSDKMQKTVTVLVERQFPHPLYGKVIKRSKKYLAHDPEEKYKLGDVVEIIESRPISKRKRFRVLRLVESGRMDLVEKYLIRRQNYQSLSKRGGKA

011101000001011111000000100000110100111111100000000000000000101111111111000000001000001001110110111111111

>1FJG_S

MPRSLKKGVFVDDHLLEKVLELNAKGEKRLIKTWSRRSTIVPEMVGHTIAVYNGKQHVPVYITENMVGHKLGEFAPTRTYRGHGKEAKATKKK

011111100100010001000000000000000101100000000000000111100000000000000101100011111010100000000

>1FJG_T

MAQKKPKRNLSALKRHRQSLKRRLRNKAKKSAIKTLSKKAVQLAQEGKAEEALKIMRKAESLIDKAAKGSTLHKNAAARRKSRLMRKVRQLLEAAGAPLIGGGLSA

0000000101010111111011111111111101110110000000000000000010011001100101001111011111101110100000000000011111

>1FJG_V

GKGDRRTRRGKIWRGTYGKYRPRKKK

11111101101111011111101100

>1FXL_A

SKTNLIVNYLPQNMTQEEFRSLFGSIGEIESCKLVRDKITGQSLGYGFVNYIDPKDAEKAINTLNGLRLQTKTIKVSYARPSSASIRDANLYVSGLPKTMTQKELEQLFSQYGRIITSRILVDQVTGVSRGVGFIRFDKRIEAEEAIKGLNGQKPSGATEPITVKFA

00010101100100000000000000000000101100000011110100000000000000000000000110100011110001100101000000000000000000000001101010000000010001010000000000000000000000000000101

>1G1X_A

MRRYEVNIVLNPNLDQSQLALEKEIIQRALENYGARVEKVEELGLRRLAYPIAKDPQGYFLWYQVEMPEDRVNDLARELRIRDNVRRVMVVKSQEPFL

01110000000000000000001000000000000101010100000001000000000000000000110110001001000000101111000000

>1G1X_B

PITKEEKQKVIQEFARFPGDTGSTEVQVALLTLRINRLSEHLKVHKKDHHSHRGLLMMVGQRRRLLRYLQREDPERYREIVEKLGLRG

1001001000100001101111100010010001001100100010110110110011001001101100100001000000000000

>1G1X_C

MSTKNAKPKKEAQRRPSRKAKVKATLGEFDLRDYRNVEVLKRFLSETGKILPRRRTGLSGKEQRILAKTIKRARILGLLPFTEKLVRK

0000000000000000000000000000000100000000000000000111100000001011001100110110000010000000

>1G1X_G

PITKEEKQKVIQEFARFPGDTGSTEVQVALLTLRINRLSEHLKVHKKDHHSHRGLLMMVGQRRRLLRYLQREDPERYREIVEKLGLRG

1000001000100001101111110010010001001100100010110110110001001001101100100001000000000000

>1GAX_A

MDLPKAYDPKSVEPKWAEKWAKNPFVANPKSGKPPFVIFMPPPNVTGSLHMGHALDNSLQDALIRYKRMRGFEAVWLPGTDHAGIATQVVVERLLLKEGKTRHDLGREKFLERVWQWKEESGGTILKQLKRLGASADWSREAFTMDEKRSRAVRYAFSRYYHEGLAYRAPRLVNWCPRCETTLSDLEVETEPTPGKLYTLRYEVEGGGFIEIATVRPETVFADQAIAVHPEDERYRHLLGKRARIPLTEVWIPILADPAVEKDFGTGALKVTPAHDPLDYEIGERHGLKPVSVINLEGRMEGERVPEALRGLDRFEARRKAVELFREAGHLVKEEDYTIALATCSRCGTPIEYAIFPQWWLRMRPLAEEVLKGLRRGDIAFVPERWKKVNMDWLENVKDWNISRQLWWGHQIPAWYCEDCQAVNVPRPERYLEDPTSCEACGSPRLKRDEDVFDTWFSSALWPLSTLGWPEETEDLKAFYPGDVLVTGYDILFLWVSRMEVSGYHFMGERPFKTVLLHGLVLDEKGQKMSKSKGNVIDPLEMVERYGADALRFALIYLATGGQDIRLDLRWLEMARNFANKLYNAARFVLLSREGFQAKEDTPTLADRFMRSRLSRGVEEITALYEALDLAQAAREVYELVWSEFCDWYLEAAKPALKAGNAHTLRTLEEVLAVLLKLLHPMMPFLTSELYQALTGKEELALEAWPEPGGRDEEAERAFEALKQAVTAVRALKAEAGLPPAQEVRVYLEGETAPVEENLEVFRFLSRADLLPERPAKALVKAMPRVTARMPLEGLLDVEEWRRRQEKRLKELLALAERSQRKLASPGFREKAPKEVVEAEEARLKENLEQAERIREALSQIG

0000110000000000000000000000000000000000000000000000000000000000000000000000000000000000000000000000000000000000000000000000000000000000000000000000000000000000000000000000000000000000000000000000000000000000000011100000000000000000000000000000000000000000000110010000100000000100100000000000000000000000000000000000000000000000000000001000000000000000000000000000000000000000000000001000000000000100000000000000000000000000000000000000000000000000000000000000000000000000000000000000000000001000000000000000000000000000000000000000100000000000000000000000000100100101011001011001100110110010000000000000000000000000000000000000000000100100010001000110010000000000000000000000000000000000000000000000000000000000000000000000001001100100000100000000000000000000000000100000000000001000000000000000000000010010000000100100010001110011100100000010001000000000000000

>1GTF_Q

MYTNSDFVVIKALEDGVNVIGLTRGADTRFHHSEKLDKGEVLIAQFTEHTSAIKVRGKAYIQTRHGVIESEGKK

00000000000000010000000000011101111010000000000000000101000000000000000000

>1H2C_A

ADDTIDHASHTPGSVSSAFILEAMVNVISGPKVLMKQIPIWLPLGVADQKTYSFDSTTAAIMLASYTITHFGKATNPLVRVNRLGPGIPDHPLRLLRIGNQAFLQEFVLPPVQLPQYFTFDLTALKLITQPLPAATWTDD

00000000000000000000000000000000000000000000000000000000000000000000111110000001000000000000000000000000000000000000100000000000000000000000

>1H3E_A

MAGTGHTPEEALALLKRGAEEIVPEEELLAKLKEGRPLTVKLGADPTRPDLHLGHAVVLRKMRQFQELGHKVVLIIGDFTGMIGDPSGRSKTRPPLTLEETRENAKTYVAQAGKILRQEPHLFELRYNSEWLEGLTFKEVVRLTSLMTVAQMLEREDFKKRYEAGIPISLHELLYPFAQAYDSVAIRADVEMGGTDQRFNLLVGREVQRAYGQSPQVCFLMPLLVGLDGREKMSKSLDNYIGLTEPPEAMFKKLMRVPDPLLPSYFRLLTDLEEEEIEALLKAGPVPAHRVLARLLTAAYALPQIPPRIDRAFYESLGYAWEAFGRDKEAGPEEVRRAEARYDEVAKGGIPEEIPEVTIPASELKEGRIWVARLFTLAGLTPSNAEARRLIQNRGLRLDGEVLTDPMLQVDLSRPRILQRGKDRFVRVRLSD

000000000000000000000000000000000000000000000000000000000000000000000000000000000000000000000000000000000000000000000000000000000000000000000000000000000000000000001110000000000000000000000000000000000000000000000000000000000000000000000000000000000000000000000000000000000000000000000000000000000000000000000000000000000000000000000000000000000000000000000000000000000000000000000000000000000000000000000000000000000000000000000000

>1HR0_W

AKEKDTIRTEGVVTEALPNATFRVKLDSGPEILAYISGKMRMHYIRILPGDRVVVEITPYDPTRGRIVYRK

01010000000000111111011000000000101101101101111000000000000000010100000

>1I6U_A

MSLMDPLANALNHISNCERVGKKVVYIKPASKLIGRVLKVQDNGYIGEFEFIEDGRAGIFKVELIGKINKCGAIKPRFPVKKFGYEKFEKRYLPARDFGILIVSTTQGVSHEEAKKRGLGGRLLAYVY

00000000000000000000000000000000000000000000000000000000000000000000000000000000000000000000000000000000000000000000011111000000

>1J1U_A

MDEFEMIKRNTSEIISEEELREVLKKDEKSAYIGFEPSGKIHLGHYLQIKKMIDLQNAGFDIIILLADLHAYLNQKGELDEIRKIGDYNKKVFEAMGLKAKYVYGSEFQLDKDYTLNVYRLALKTTLKRARRSMELIAREDENPKVAEVIYPIMQVNDIHYLGVDVAVGGMEQRKIHMLARELLPKKVVCIHNPVLTGLDGEGKMSSSKGNFIAVDDSPEEIRAKIKKAYCPAGVVEGNPIMEIAKYFLEYPLTIKRPEKFGGDLTVNSYEELESLFKNKELHPMDLKNAVAEELIKILEPIRKRL

000000000000000000000000000000000000000000000000000000000000000000000000000000000000000000000000000000000000000000000000000000000000000000000010100000000000000000000000000000000000000000000000000000000000000000000000000000000011111110000000000000000000000000001000000000000000000000111101000000000000000000

>1J2B_A

MSRGDKMLKFEIKARDGAGRIGKLEVNGKKIETPAIMPVVNPKQMVVEPKELEKMGFEIIITNSYIIYKDEELRRKALELGIHRMLDYNGIIEVDSGSFQLMKYGSIEVSNREIIEFQHRIGVDIGTFLDIPTPPDAPREQAVKELEITLSRAREAEEIKEIPMNATIQGSTYTDLRRYAARRLSSMNFEIHPIGGVVPLLESYRFRDVVDIVISSKMALRPDRPVHLFGAGHPIVFALAVAMGVDLFDSASYALYAKDDRYMTPEGTKRLDELDYFPCSCPVCSKYTPQELREMPKEERTRLLALHNLWVIKEEIKRVKQAIKEGELWRLVDERARSHPKLYSAYKRLLEHYTFLEEFEPITKKSALFKISNESLRWPVVRRAKERAKSINERFGELVEHPIFGRVSRYLSLTYPFAQSEAEDDFKIEKPTKEDAIKYVMAIAEYQFGEGASRAFDDAKVELSKTGMPRQVKVNGKRLATVRADDGLLTLGIEGAKRLHRVLPYPRMRVVVNKEAEPFARKGKDVFAKFVIFADPGIRPYDEVLVVNENDELLATGQALLSGREMIVFQYGRAVKVRKGVE

000000000000100000000010000000000000001110100000000000000000100011001000000000000000000000000010011100110010000000000000000000100101000101000000000000000000000000000010000110000000000000000000001001001101111100000000000000000000111110000000000000001111011100001000000110000000000000000000000000000000000000000000000000000001000000000000001000000000000000000000000000101000000000000000000000000000000000000000000000000110100110110100000000000000000000000000000001111101011010001100101110000100000000000000000000000010001000011010110000000000000000000000000000000000000000111001011000

>1JBR_A

ATWTCINQQLNPKTNKWEDKRLLYSQAKAESNSHHAPLSDGKTGSSYPHWFTNGYDGNGKLIKGRTPIKFGKADCDRPPKHSQNGMGKDDHYLLEFPTFPDGHDYKFDSKKPKEDPGPARVIYTYPNKVFCGIVAHQRGNQGDLRLCSH

00000000000000000000000000000000000000000101110001010000000000001000000000000000000000000000000000000000000001101000000000000000000000000000011000000

>1JID_A

MACAAARSPADQDRFICIYPAYLNNKKTIAEGRRIPISKAVENPTATEIQDVCSAVGLNVFLEKNKMYSREWNRDVQYRGRVRVQLKQEDGSLCLVQFPSRKSVMLYAAEMIPKLKTRTQLEHHHHHH

00000000000011111010010000011000110110100000000000000000000000000001110000000000001000100000000000001100000000000000000000000000

>1JJ2_1

GKKSKATKKRLAKLDNQNSRVPAWVMLKTDEVQRNHKRRHWRRNDTDE

111111011110101110111101101111000111111111101101

>1JJ2_2

MQMPRRFNTYCPHCNEHQEHEVEKVRSGRQTGMKWIDRQRERNSGIGNDGKFSKVPGGDKPTKKTDLKYRCGECGKAHLREGWRAGRLEFQE

11110011110110111000000010111111111101100110111111101100111111111101010000011111111101000010

>1JJ2_A

GRRIQGQRRGRGTSTFRAPSHRYKADLEHRKVEDGDVIAGTVVDIEHDPARSAPVAAVEFEDGDRRLILAPEGVGVGDELQVGVDAEIAPGNTLPLAEIPEGVPVCNVESSPGDGGKFARASGVNAQLLTHDRNVAVVKLPSGEMKRLDPQCRATIGVVGGGGRTDKPFVKAGNKHHKMKARGTKWPNVRGVAMNAVDHPFGGGGRQHPGKPKSISRNAPPGRKVGDIASKRTGRGGNE

11100111111111111101111111000010100000000000001011110000000000000000000000000000000000000000000000000000000001111100111111110000000000000001110101000000000000000111101111111011110111111111111111111110101111111111111111011111111111111111000

>1JJ2_B

PQPSRPRKGSLGFGPRKRSTSETPRFNSWPSDDGQPGVQGFAGYKAGMTHVVLVNDEPNSPREGMEETVPVTVIETPPMRAVALRAYEDTPYGQRPLTEVWTDEFHSELDRTLDVPEDHDPDAAEEQIRDAHEAGDLGDLRLITHTVPDAVPSVPKKKPDVMETRVGGGSVSDRLDHALDIVEDGGEHAMNDIFRAGEYADVAGVTKGKGTQGPVKRWGVQKRKGKHARQGWRRRIGNLGPWNPSRVRSTVPQQGQTGYHQRTELNKRLIDIGEGDEPTVDGGFVNYGEVDGPYTLVKGSVPGPDKRLVRFRPAVRPNDQPRLDPEVRYVSNESNQG

1111111111111111110101001011000000000000000010011100000000100000000001000000000000001010000111111110100000000011111111100000000000000000000010100000000001111100111000000000000000000000000000000000000000000111111110111001111111101111111111111111111111111111001011111110000000000000111111111000000001111111011001010011010000000000000001111

>1JJ2_C

MQATIYDLDGNTDGEVDLPDVFETPVRSDLIGKAVRAAQANRKQDYGSDEYAGLRTPAESFGSGRGQAHVPKLDGRARRVPQAVKGRSAHPPKTEKDRSLDLNDKERQLAVRSALAATADADLVADRGHEFDRDEVPVVVSDDFEDLVKTQEVVSLLEALDVHADIDRADETKIKAGQGSARGRKYRRPASILFVTSDEPSTAARNLAGADVATASEVNTEDLAPGGAPGRLTVFTESALAEVAER

000000000000000000000000001001001001011111111111100111111001001111111111110111111111101111111111110111111111100100000000000000100000000000000000000111100100000000000001010011111110110111111100000000010110111100001101111001001100000000000000000001

>1JJ2_D

SSESESGGDFHEMREPRIEKVVVHMGIGHGGRDLANAEDILGEITGQMPVRTKAKRTVGEFDIREGDPIGAKVTLRDEMAEEFLQTALPLAELATSQFDDTGNFSFGVEEHTEFPSQEYDPSIGIYGLDVTVNLVRPGYRVAKRDKASRSIPTKHRLNPADAVAFIESTYDVEVSE

00000000010111001001010101110000000000000000001111111111000101000000000111010000000000000000000111111010111000000000000000000000101010001111101111001111111100000000000000000000

>1JJ2_E

PRVELEIPEDVDAEQDHLDITVEGDNGSVTRRLWYPDIDVSVDGDTVVIESDEDNAKTMSTIGTFQSHIENMFHGVTEGWEYGMEVFYSHFPMQVNVEGDEVVIENFLGEKAPRRTTIHGDTDVEIDGEELTVSGPDIEAVGQTAADIEQLTRINDKDVRVFQDGVYITRKPNRGDA

000000000000000000000000000000010010000000000000000000111011101110110010000000000100000001100000000000000001111100000010000000000000000001100110010001001010110111000000001000000

>1JJ2_F

PVYVDFDVPADLEDDALEALEVARDTGAVKKGTNETTKSIERGSAELVFVAEDVQPEEIVMHIPELADEKGVPFIFVEQQDDLGHAAGLEVGSAAAAVTDAGAAATVLEEIADKVEELR

00000000000000000000000000000111111001000100000000000110001000000000000000000001000000001111110000000000000000000000000

>1JJ2_G

MSAESERKTETIPEWKQEEVDAIVEMIESYESVGVVNIAGIPSRQLQDMRRDLHGTAELRVSRNTLLERALDDVDDGLEDLNGYITGQVGLIGTDDNPFSLFQELEASKTPAPIGAGEVAPNDIVIPEGDTGVDPGPFVGELQSVGADARIQEGSIQVLSDSTVLDTGEEVSQELSNVLNELGIEPKEVGLDLRAVFADGVLFEPEELELDIDEYRSDIQAAAGRAFNLSVNADYPTATTAPTMLQSDRGNAKSLALQAAIEDPEVVPDLVSKADAQVRALASQIDDEEALPEELQGVEADVATEEPTDDQDDDTASEDDADADDAAEEADDDDDDDEDAGDALGAMF

000000000000100110010000000000000000000000000000000000000000001111001000000000000000000000000000000000000000000000000000000000000000000000000000000000000000000000000000000000000000000000000000000000000000000000000000000000000000000000000000000000000000000000000000000000000000000000000000000000000000000000000000000000000000000000000000000000000000

>1JJ2_H

KPGAMYRNSSKPAYTRREYISGIPGKKIAQFDMGNNGAGPTYPAQVELVVEKPVQIRHNALEAARVAANRYVQNSGAAANYKFRIRKFPFHVIRENKAAAAAAAAAAADGMRAPFGKPVGTAARVHGANHIFIAWVNPDPNVEEAWRRAKMKVTPTINIDSSPAGNA

11001010001111111110000001100001000110000000000000010000101100100000010000000000000101110100011100000000000001111111000000010000000000000000000000110111110100000000000

>1JJ2_I

MSVAEFDADVIVDARDCIMGRVASQVAEQALDGETVAVVNAERAVITGREEQIVEKYEKRVDIGNDNGYFYPKRPDGIFKRTIRGMLPHKKQRGREAFESVRVYLGNPYDEDGEVLDGTSLDRLSNIKFVTLGEISETLGANKTW

0000000000000000011110011011001000000000000000111001000100110011111111011100000111011111111110100010011100000000000000000000001100000000001100000

>1JJ2_J

MEALGADVTQGLEKGSLITCADNTGARELKVISVHGYSGTKNRHPKAGLGDKITVSVTKGTPEMRRQVLEAVVVRQRKPIRRPDGTRVKFEDNAAVIVDENEDPRGTELKGPIAREVAQRFGSVASAATMIV

111000001111110110000000000001011111111111111100000001010010000011010000000011001000001010001000000000000000000000000000000000000000

>1JJ2_K

TSKKKRQRGSRTHGGGSHKNRRGAGHRGGRGDAGRDKHEFHNHEPLGKSGFKRPQKVQEEAATIDVREIDENVTLLAADDVAEVEDGGFRVDVRDVVEEADDADYVKVLGAGQVRHELTLIADDFSEGAREKVEGAGGSVELTDLGEERQAEAEETEDADADEE

11111101111111111111111111111111111011111100011101111111100000101010011000000000000000000000000000000000001011111010000000000110000100000000000000001000000000000000

>1JJ2_L

ARSAYSYIREAWKRPKEGQIAELMWHRMQEWRNEPAVVRIERPTRLDRARSLGYKAKQGIIVVRVAIRKGSSRRTRFNKGRRSKRMMVNRITRKKNIQRIAEERANRKFPNLRVLNSYSVGEDGRHKWHEVILIDPDHPAIKSDDQLSWISRTRHRLRTFRGLTSAGRRCRGLRGQGKGSEKVRPSLRVNGAKA

11101000100011110000000010001001001100100100110101110011111010000001111011111111111111111101111110110001001111111001001001111010000000101111001011001001110111011110100111111101111011111111101111

>1JJ2_M

ATGPRYKVPMRRRREARTDYHQRLRLLKSGKPRLVARKSNKHVRAQLVTLGPNGDDTLASAHSSDLAEYGWEAPTGNMPSAYLTGLLAGLRAQEAGVEEAVLDIGLNSPTPGSKVFAIQEGAIDAGLDIPHNDDVLADWQRTRGAHIAEYDEQLEEPLYSGDFDAADLPEHFDELRETLLDGDIEL

111111111111110111111110110110101010111111010101000011111001000000000000000111000000000000100000000011010101111111010000000000000111001000001000011001001000011101000000000000000000000000

>1JJ2_O

TDLSAQKRLAADVLDVGKNRVWFNPERQGDIADAITREDVRELVDEGAIQAKDKKGNSRGRARERQKKRAKGHQKGAGSRKGKAGARQNSKEDWESRIRAQRTKLRELRDEGTLSSSQYRDLYDKAGGGEFDSVADLERYIDANHGDA

1111001110000000111101101001000000111000100000000001011111111110111111101111110111111111001001111111010001001000000110110011100101001100001000000000

>1JJ2_P

PSSNGPLEGTRGKLKNKPRDRGTSPPQRAVEEFDDGEKVHLKIDPSVPNGRFHPRFDGQTGTVEGKQGDAYKVDIVDGGKEKTIIVTAAHLRRQE

11111111101111111110111010110000000001010100110111001011011100000011110000001111011110100001011

>1JJ2_Q

GISYSVEADPDTTAKAMLRERQMSFKHSKAIAREIKGKTAGEAVDYLEAVIEGDQPVPFKQHNSGVGHKSKVDGWDAGRYPEKASKAFLDLLENAVGNADHQGFDGEAMTIKHVAAHKVGEQQGRKPRAMGRASAWNSPQVDVELILEEPEVED

1111100000000011101000011110100010010000000000000000000000010111111111100111001101100000000011000100110000000000101111100100111111111111111000000000000000

>1JJ2_R

SWDVIKHPHVTEKAMNDMDFQNKLQFAVDDRASKGEVADAVEEQYDVTVEQVNTQNTMDGEKKAVVRLSEDDDAQEVASRIGVF

110001001011100000001010000000001100000000000000000011110100011010100000000000000000

>1JJ2_S

SKQPDKQRKSQRRAPLHERHKQVRATLSADLREEYGQRNVRVNAGDTVEVLRGDFAGEEGEVINVDLDKAVIHVEDVTLEKTDGEEVPRPLDTSNVRVTDLDLEDEKREARLESEDDSA

11111111101110111001100111000001000001101011000000111100000000001010100000000011111000101101011010000000101100100001100

>1JJ2_T

PRTRECDYCGTDIEPGTGTMFVHKDGATTHFCSSKCENNADLGREARNLEWTDTARGEAGEAEDEA

000000000000000111000000000000000110110001010000011000010000000000

>1JJ2_U

TVLHVQEIRDMTPAEREAELDDLKTELLNARAVQAAGGAPENPGRIKELRKAIARIKTIQGEEGDLQENE

1001111010000000000000010000001001000000011101110110111011100100000000

>1JJ2_V

MHALVQLRGEVNMHTDIQDTLEMLNIHHVNHCTLVPETDAYRGMVAKVNDFVAFGEPSQETLETVLATRAEPLEGDADVDDEWVAEHTDYDDISGLAFALLSEETTLREQGLSPTLRLHPPRGGHDGVKHPVKEGGQLGKHDTEGIDDLLEAMR

0000000111111111101100101011111000000011111101101110000000000000000010000110000000000000000000000000000000010000111011111111101111101100000000000000000001

>1JJ2_W

SASDFEERVVTIPLRDARAEPNHKRADKAMILIREHLAKHFSVDEDAVRLDPSINEAAWARGRANTPSKIRVRAARFEEEGEAIVEAETAE

0000000100101011011111111011011001000011000000001101001100101110100110100000000000000000000

>1JJ2_X

ADNEEDVEAEEEYTELTDISGVGPSKAESLREAGFESVEDVRGADQSALADVSGIGNALAARIKADVGGLEVESETEAEVEEEGGEEAPDEDVETELQARGLTEKTPDLSDEDARLLTQRHRVGKPQFNRQDHHKKKRVSTSWRKPRGQLSKQRRGIKGKGDTVEAGFRSPTAVRGKHPSGFEEVRVHNVDDLEGVDGDTEAVRIASKVGARKRERIEEEAEDAGIRVLNPTYVEVEVSE

000000000000000000000000000000000000000000000000000000000000000000000000000000000000000000000000000000000000000000110111111011110111011111100111101111110111111110011111111100111110000001011000000000000001111111111111000000000010111010000000

>1JJ2_Y

RTGRFGPRYGLKIRVRVADVEIKHKKKHKCPVCGFKKLKRAGTGIWMCGHCGYKIAGGCYQPETVAGKAVMKA

1011011111111101000000001010000010001011011000000010000000010000000000000

>1JJ2_Z

TGAGTPSQGKKNTTTHTKCRRCGEKSYHTKKKVCSSCGFGKSAKRRDYEWQSKAGE

11111111111111111111111011111111001100101111110111111100

>1K8W_A

MGHHHHHHHHHHSSGHIEGRHMDINGVLLLDKPQGMSSNDALQKVKRIYNANRAGHTGALDPLATGMLPICLGEATKFSQYLLDSDKRYRVIARLGQRTDTSDADGQIVEERPVTFSAEQLAAALDTFRGDIEQIPSMYSALKYQGKKLYEYARQGIEVPREARPITVYELLFIRHEGNELELEIHCSKGTYIRTIIDDLGEKLGCGAHVIYLRRLAVSKYPVERMVTLEHLRELVEQAEQQDIPAAELLDPLLMPMDSPASDYPVVNLPLTSSVYFKNGNPVRTSGAPLEGLVRVTEGENGKFIGMGEIDDEGRVAPRRLVVEYPA

000000000000000000000000000000000000101001100100000011110000110000000000000110110010000000000000000010100000000000000000000000000000000000111010000101000100000000010000000000000000000000000001010000000000000000000000000000000000000000000000000000000000000000000000000000000000000000000000000000000000000000000000000000010000000

>1KNZ_A

LGSMESTQQMAVSIINSSFEAAVVAATSALENMGIEYDYQDIYSRVKNKFDFVMDDSGVKNNPIGKAITIDQALNNKFGSAIRNRNWLADTSRPAKLDEDVNKLRMMLSSKGIDQKMRVLNACFSVKRIPGKSSSIIKCTKLMRDKLERGEVEVDDSFVDEKME

00000000000000000000000000000000000000000000000000000000100011001101100000000001001100100000100110010001000000000000000000000000001111100000000000000000000000000000

>1KQ2_A

MIANENIQDKALENFKANQTEVTVFFLNGFQMKGVIEEYDKYVVSLNSQGKQHLIYKHAISTYTVETEGQASTESEE

00000001000000000000000000100000000000001100000000000000110000000000000000000

>1LAJ_A

MAQNGTGGGSRRPRRGRRNNNNNNSTARDKALLALTQQVNRLANIASSSAPSLQHPTFIASKKCRAGYTYTSLDVRPTRTEKDKSFGQRLIIPVPVSEYPKKKVSCVQVRLNPSPKFNSTIWVSLRRLDETTLLTSENVFKLFTDGLAVLIYQHVPTGIQPNNKITFDMSNVGAEIGDMGKYALIVYSKDDVLEADEMVIHIDIEHQRIPSASTLPV

0000000000000000000000000000000000000000000000000000000000000000011000000000000000000000000000000000000000000000000000000000000000000000000000000000000000000000000000000000000000000000000000000000000000000001000000000

>1LNG_A

MIIWPSYIDKKKSRREGRKVPEELAIEKPSLKDIEKALKKLGLEPKIYRDKRYPRQHWEICGCVEVDYKGNKLQLLKEICKIIKGKN

110100100000111001101100000000000000000000000000001111101000000000111011100010000000000

>1M8V_A

GAMAERPLDVIHRSLDKDVLVILKKGFEFRGRLIGYDIHLNVVLADAEMIQDGEVVKRYGKIVIRGDNVLAISPTEE

00010101100100100000000000000000001110100000000000000000000000000000000000000

>1MFQ_C

MRGSHHHHHHKHGQFTLRDMYEQFQNIMKMGPFSQILGMIPGFGTDFMSKGNEQESMARLKKLMTIMDSMNDQELDSTDGAKVFSKQPGRIQRVARGSGVSTRDVQELLTQYTKFAQMVKKMGGIKGLF

000000000000000000000000000000000000000000000000000000000000000010011110000000000000001011001000111000000000000000000000000000000

>1MMS_A

AKKVAAQIKLQLPAGKATPAPPVGPALGQHGVNIMEFCKRFNAETADKAGMILPVVITVYEDKSFTFIIKTPPASFLLKKAAGIEKGSSEPKRKIVGKVTRKQIEEIAKTKMPDLNANSLEAAMKIIEGTAKSMGIEVVD

00000000111000000000000000001000000000000000000000000000000000000000010011110010000001111111100000000000000000100111110001001100110111000000

>1MZP_A

MLADKESLIEALKLALSTEYNVKRNFTQSVEIILTFKGIDKKGDLKLREIVPLPKQPSKAKRVLVVPSSEQLEYAKKASPKVVITREELQKLQGQKRPVKKLARQNEWFLINQESALAGRILGPALGPRGKFPTPLPNTADISEYINRFKRSVLVKTKDQPQVQVFIGTEDKPEDLAENAIAVLNAIENKAKVETNLRNIYVKTTGKAVKVKR

000000000000000000000000000000000000000000000000000000000000000000000000000000000000000000000000000000000000000000000000000000000000000000000000000000000000000000000000000000000000000000000000000000000000011100000

>1N35_A

MSSMILTQFGPFIESISGITDQSNDVFEDAAKAFSMFTRSDVYKALDEIPFSDDAMLPIPPTIYTKPSHDSYYYIDALNRVRRKTYQGPDDVYVPNCSIVELLEPHETLTSYGRLSEAIENRAKDGDSQARIATTYGRIAESQARQIKAPLEKFVLALLVAEAGGSLYDPVLQKYDEIPDLSHNCPLWCFREICRHISGPLPDRAPYLYLSAGVFWLMSPRMTSAIPPLLSDLVNLAILQQTAGLDPSLVKLGVQICLHAAASSSYSWFILKTKSIFPQNTLHSMYESLEGGYCPNLEWLEPRSDYKFMYMGVMPLSAKYARSAPSNDKKARELGEKYGLSSVVGELRKRTKTYVKHDFASVRYIRDAMACTSGIFLVRTPTETVLQEYTQSPEIKVPIPQKDWTGPIGEIRILKDTTSSIARYLYRTWYLAAARMAAQPRTWDPLFQAIMRSQYVTARGGSGAALRESLYAINVSLPDFKGLPVKAATKIFQAAQLANLPFSHTSVAILADTSMGLRNQVQRRPRSIMPLNVPQQQVSAPHTLTADYINYHMNLSPTSGSAVIEKVIPLGVYASSPPNQSINIDISACDASITWDFFLSVIMAAIHEGVASSSIGKPFMGVPASIVNDESVVGVRAARPISGMQNMIQHLSKLYKRGFSYRVNDSFSPGNDFTHMTTTFPSGSTATSTEHTANNSTMMETFLTVWGPEHTDDPDVLRLMKSLTIQRNYVCQGDDGLMIIDGTTAGKVNSETIQNDLELISKYGEEFGWKYDIAYDGTAEYLKLYFIFGCRIPNLSRHPIVGKERANSSAEEPWPAILDQIMGVFFNGVHDGLQWQRWIRYSWALCCAFSRQRTMIGESVGYLQYPMWSFVYWGLPLVKAFGSDPWIFSWYMPTGDLGMYSWISLIRPLMTRWMVANGYVTDRCSTVFGNADYRRCFNELKLYQGYYMAQLPRNPKKSGRAASREVREQFTQALSDYLMQNPELKSRVLRGRSEWEKYGAGIIHNPPSLFDVPHKWYQGAQEAAIATREELAEMDETLMRARRHSYSSFSKLLEAYLLVKWRMCEAREPSVDLRLPLCAGIDPLNSDPFLKMVSVGPMLQSTRKYFAQTLFMAKTVSGLDVNAIDSALLRLRTLGADKKALTAQLLMVGLQESEADALAGKIMLQDVNTVQLARVVNLAVPDTWMSLDFDSMFKHHVKLLPKDGRHLNTDIPPRMGWLRAILRFLGAGMVMTATGVAVDIYLEDIHGGGRSLGQRFMTWMRQEGRSA

0000000000000000000000000000000000000000000000000000000000000000000000000000000000000000000000000000000000000000000000000000000000000000000000000001000000000000000000000000000000000000000000000000000000000000000000000000000000000000000000000000000000000000000000000000000000000000000000000000000000000000000000000000000000000000000000000000000000000000000000000000000000000000000000000000000000000000000000000000000000000000000000000000000000000000000001001011110100000000000000000000010001000000000000000000000001110100000000011100000000000000000000000000001111001100000000000000000000000000000000000000000000000000000000000000000000000000000000000000000000000100000000000000000001111000000000000000000000000000000000000000000000000000000000000000000000000000000000000000000000000010000000000000110000100010100100000011001000100000000000000000000000000000000000000000000000000000000000000000000000000000000000000000000000000000000000000000000000000000000000000000000000000000000000000000000000000000000000000000000000000000000000000000000000000000000000000000000000000000000000000000000000000000000000100000000001000000000000000000000000000000000000000000000000000000000001000010011010000000000000000000000000000000000000000000000000000000000000000000000000000000000

>1N78_A

MVVTRIAPSPTGDPHVGTAYIALFNYAWARRNGGRFIVRIEDTDRARYVPGAEERILAALKWLGLSYDEGPDVGGPHGPYRQSERLPLYQKYAEELLKRGWAYRAFETPEELEQIRKEKGGYDGRARNIPPEEAEERARRGEPHVIRLKVPRPGTTEVKDELRGVVVYDNQEIPDVVLLKSDGYPTYHLANVVDDHLMGVTDVIRAEEWLVSTPIHVLLYRAFGWEAPRFYHMPLLRNPDKTKISKRKSHTSLDWYKAEGFLPEALRNYLCLMGFSMPDGREIFTLEEFIQAFTWERVSLGGPVFDLEKLRWMNGKYIREVLSLEEVAERVKPFLREAGLSWESEAYLRRAVELMRPRFDTLKEFPEKARYLFTEDYPVSEKAQRKLEEGLPLLKELYPRLRAQEEWTEAALEALLRGFAAEKGVKLGQVAQPLRAALTGSLETPGLFEILALLGKERALRRLERALA

000000001000000000000000000000000000000000110010000000000000000000000000000000000000000000000000000000000010100100010000000000000000000000000000101000000000000000100101000100001001100001100000000000000000001111100000000000000000000000101000111000010000000000000000000000011101000001000000000000001011101100011001000100100000000000000000000000000000000000001100000000000000000000000000000000000000000000000000000000001000000000110011001000000111111100000000000000000000

>1NB7_A

SMSYTWTGALITPCAAEESKLPINPLSNSLLRHHNMVYATTSRSASLRQKKVTFDRLQVLDDHYRDVLKEMKAKASTVKAKLLSIEEACKLTPPHSAKSKFGYGAKDVRNLSSRAVNHIRSVWEDLLEDTETPIDTTIMAKSEVFCVQPEKGGRKPARLIVFPDLGVRVCEKMALYDVVSTLPQAVMGSSYGFQYSPKQRVEFLVNTWKSKKCPMGFSYDTRCFDSTVTESDIRVEESIYQCCDLAPEARQAIRSLTERLYIGGPLTNSKGQNCGYRRCRASGVLTTSCGNTLTCYLKATAACRAAKLQDCTMLVNGDDLVVICESAGTQEDAAALRAFTEAMTRYSAPPGDPPQPEYDLELITSCSSNVSVAHDASGKRVYYLTRDPTTPLARAAWETARHTPINSWLGNIIMYAPTLWARMILMTHFFSILLAQEQLEKALDCQIYGACYSIEPLDLPQIIERLHGLSAFTLHSYSPGEINRVASCLRKLGVPPLRTWRHRARSVRAKLLSQGGRAATCGRYLFNWAVRTKLKLTPIPAASQLDLSGWFVAGYSGGDIYHSLSRARPR

000000000000010000000000000000000000000000000000000000000000000000000000000000000000000000001011100000000000000000000000000000000000000000001000000000000000010101000001000000000000000000000000000000000000000000000000000000001000000000000000000000000000000000000000000000000000000001000011001000000000000000000000000001000000000000000000000000000000000000000000000000000000000000000000000000000000000000001000000000000000000000000000000000000000010010100000000000000000000000000000000000000000000000000000000000000000000000000000000000000000000000000000000110100000000000

>1OOA_A

GGPYLQILEQPKQRGFRFRYVCEGPSHGGLPGASSEKNKKSYPQVKICNYVGPAKVIVQLVTNGKNIHLHAHSLVGKHCEDGVCTVTAGPKDMVVGFANLGILHVTKKKVFETLEARMTEACIRGYNPGLLVHSDLAYLQAEGGGDRQLTDREKEIIRQAAVQQTKEMDLSVVRLMFTAFLPDSTGSFTRRLEPVVSDAIYDSKAPNASNLKIVRMDRTAGCVTGGEEIYLLCDKVQKDDIQIRFYEEEENGGVWEGFGDFSPTDVHRQFAIVFKTPKYKDVNITKPASVFVQLRRKSDLETSEPKPFLYYPEIKDKEEVQRKRQK

00000000000000001011011011110000000010010000000000000000000000000000000000000000000000000000000000000000011110000000000000000000000000000000000000000000000000000000000000000000000000000000000000000000000111000001000000000000000000000010010000000000000000000000000000010000000000000000000000000000000000000100000000000000000000

>1PGL_2

METNLFKLSLDDVETPKGSMLDLKISQSKIALPKNTVGGTILRSDLLANFLTEGNFRASVDLQRTHRIKGMIKMVATVGIPENTGIALACAMNSSIRGRASSDIYTICSQDCELWNPACTKAMTMSFNPNPCSDAWSLEFLKRTGFHCDIICVTGWTATPMQDVQVTIDWFISSQECVPRTYCVLNPQNPFVLNRWMGKLTFPQGTSRSVKRMPLSIGGGAGAKSAILMNMPNAVLSMWRYFVGDLVFEVSKMTSPYIKCTVSFFIAFGNLADDTINFEAFPHKLVQFGEIQEKVVLKFSQEEFLTAWSTQVRPATTLLADGCPYLYAMVHDSSVSTIPGDFVIGVKLTIIENMCAYGLNPGISGSRLLG

0000000000000000000000000000000000000000000000000000000000000000000000101000000000000000000000000000000000000000000000000001110000000000000000000000000000000000000000000000000000010000000001010000000000000000000000000000000000000000000000000000000000000000000000000000000000000000000000000000000000000000000000000000000000000000000000000000000000000100000000000000000000

>1Q2R_A

MVEATAQETDRPRFSFSIAAREGKARTGTIEMKRGVIRTPAFMPVGTAATVKALKPETVRATGADIILGNTYHLMLRPGAERIAKLGGLHSFMGWDRPILTDSGGYQVMSLSSLTKQSEEGVTFKSHLDGSRHMLSPERSIEIQHLLGSDIVMAFDECTPYPATPSRAASSMERSMRWAKRSRDAFDSRKEQAENAALFGIQQGSVFENLRQQSADALAEIGFDGYAVGGLAVGEGQDEMFRVLDFSVPMLPDDKPHYLMGVGKPDDIVGAVERGIDMFDCVLPTRSGRNGQAFTWDGPINIRNARFSEDLKPLDSECHCAVCQKWSRAYIHHLIRAGEILGAMLMTEHNIAFYQQLMQKIRDSISEGRFSQFAQDFRARYFARNS

00000000000000000000000000000000000000000000101000010000000000000000010010000000000000000000000000000000011001100100000000000010000000000000000000000000000000000000000000000000000000000000000000000000000000000000000000000000000000000000000000000000000000000000000000000000000000000100110011000000000000100000000000000000000000000000000000010000000000000000000000000000000000000000000000

>1QF6_A

MPVITLPDGSQRHYDHAVSPMDVALDIGPGLAKACIAGRVNGELVDACDLIENDAQLSIITAKDEEGLEIIRHSCAHLLGHAIKQLWPHTKMAIGPVIDNGFYYDVDLDRTLTQEDVEALEKRMHELAEKNYDVIKKKVSWHEARETFANRGESYKVSILDENIAHDDKPGLYFHEEYVDMCRGPHVPNMRFCHHFKLMKTAGAYWRGDSNNKMLQRIYGTAWADKKALNAYLQRLEEAAKRDHRKIGKQLDLYHMQEEAPGMVFWHNDGWTIFRELEVFVRSKLKEYQYQEVKGPFMMDRVLWEKTGHWDNYKDAMFTTSSENREYCIKPMNCPGHVQIFNQGLKSYRDLPLRMAEFGSCHRNEPSGSLHGLMRVRGFTQDDAHIFCTEEQIRDEVNGCIRLVYDMYSTFGFEKIVVKLSTRPEKRIGSDEMWDRAEADLAVALEENNIPFEYQLGEGAFYGPKIEFTLYDCLDRAWQCGTVQLDFSLPSRLSASYVGEDNERKVPVMIHRAILGSMERFIGILTEEFAGFFPTWLAPVQVVIMNITDSQSEYVNELTQKLSNAGIRVKADLRNEKIGFKIREHTLRRVPYMLVCGDKEVESGKVAVRTRRGKDLGSMDVNEVIEKLQQEIRSRSLKQLEE

000000000000000000000000000000000000000000000000000000000000000000000000000000000000000000000000000000000000000000000000000000000000000000000000000000000000000000000000000000000000000000000000000000010111100101000100101000000000000000000000000010000000000001001000000000000000000000000000000000000000000000001000100110000000000000110000000000000000000000000000001001001000001010000000000000000000000000000000000000000000000000000000000000000000000000000000000101001000000000010000000100000000000000000000000000000000000000000000000000000000000000000000000000000000000000000000000000000000000000000000000000000000000000000000000000000000000000

>1QTQ_A

SEAEARPTNFIRQIIDEDLASGKHTTVHTRFPPEPNGYLHIGHAKSICLNFGIAQDYKGQCNLRFDDTNPVKEDIEYVESIKNDVEWLGFHWSGNVRYSSDYFDQLHAYAIELINKGLAYVDELTPEQIREYRGTLTQPGKNSPYRDRSVEENLALFEKMRAGGFEEGKACLRAKIDMASPFIVMRDPVLYRIKFAEHHQTGNKWCIYPMYDFTHCISDALEGITHSLCTLEFQDNRRLYDWVLDNITIPVHPRQYEFSRLNLEYTVMSKRKLNLLVTDKHVEGWDDPRMPTISGLRRRGYTAASIREFCKRIGVTKQDNTIEMASLESCIREDLNENAPRAMAVIDPVKLVIENYQGEGEMVTMPNHPNKPEMGSRQVPFSGEIWIDRADFREEANKQYKRLVLGKEVRLRNAYVIKAERVEKDAEGNITTIFCTYDADTLSKDPADGRKVKGVIHWVSAAHALPVEIRLYDRLFSVPNPGAADDFLSVINPESLVIKQGFAEPSLKDAVAGKAFQFEREGYFCLDSRHSTAEKPVFNRTVGLRDTWAKVGE

0000000000001000000000000000000001000000000010000000000000000000000110010000000000000000000000000000000000000000000000000001110011001011100000000000000000000000000000010110100000001110000010010100000000000000011000000000000000000001111011000000000000000000000000000000000000000000000000000000000000000000000000011101111111101100100100010000100000000000000000000000000011000000000000000000000000000011110000000101100000000000000000000000000001000000000000100000000000000000000000000000000000000000000000000000000000001011000000000000000000000001100000000

>1R3E_A

MKHGILVAYKPKGPTSHDVVDEVRKKLKTRKVGHGGTLDPFACGVLIIGVNQGTRILEFYKDLKKVYWVKMRLGLITETFDITGEVVEERECNVTEEEIREAIFSFVGEYDQVPPAYSAKKYKGERLYKLAREGKIINLPPKRVKIFKIWDVNIEGRDVSFRVEVSPGTYIRSLCMDIGYKLGCGATAVELVRESVGPHTIEESLNVFEAAPEEIENRIIPLEKCLEWLPRVVVHQESTKMILNGSQIHLEMLKEWDGFKKGEVVRVFNEEGRLLALAEAERNSSFLETLRKHERNERVLTLRKVFNTR

000000000000001010010001000000111100101100000000000001101100000000000000000000101010000000000000000000000000000000001111100001010001000000000000000000000000000000000000010100000000000000000000000000000000000000000000000000000000000000000000000110100000000000000000000000000000000000000000000000000000010110100

>1RC7_A

MKMLEQLEKKLGYTFKDKSLLEKALTHVSYSKKEHYETLEFLGDALVNFFIVDLLVQYSPNKREGFLSPLKAYLISEEFFNLLAQKLELHKFIRIKRGKINETIIGDVFKALWAAVYIDSGRDANFTRELFYKLFKEDILSAIKEGRVKKDYKTILQEITQKRWKERPEYRLISVEGPHHKKKFIVEAKIKEYRTLGEGKSKKEAEQRAAEELIKLLEES

0000000000000000000000000000000000000000000000000000000000001010010000000000000000000000000000000000000000000000000000000000000000000000000000000000011011001100110010100000000000110001000000000000000111100000000000000000

>1RMV_A

SYNITNSNQYQYFAAVWAEPTPMLNQCVSALSQSYQTQAGRDTVRQQFANLLSTIVAPNQRFPDTGFRVYVNSAVIKPLYEALMKSFDTRNRIIETEEESRPSASEVANATQRVDDATVAIRSQIQLLLNELSNGHGYMNRAEFEAILPWTTAPAT

000000000000000000000000000000000000000000000000000000000000000000000000000000000000000000000000000000000000000010111011011100100000000000000000000000000000

>1RPU_A

MERAIQGNDTREQANGERWDGGSGGITSPFKLPDESPSWTEWRLYNDETNSNQDNPLGFKESWGFGKVVFKRYLRYDRTEASLHRVLGSWTGDSVNYAASRFLGANQVGCTYSIRFRGVSVTISGGSRTLQHLCEMAIRSKQELLQLTPVEVESNVSRGCPEGIETFKKESE

0000000000100000011000000000000000011111011000000000000000010000011010101000000000000000000000000000000000111010101000010101110000000000000000000000000000000000000000000000

>1S03_G

SMQDPIADMLTRIRNGQAANKAAVTMPSSKLKVAIANVLKEEGFIEDFKVEGDTKPELELTLKYFQGKAVVESIQRVSRPGLRIYKRKDELPKVMAGLGIAVVSTSKGVMTDRAARQAGLGGEIICYVA

000010010001000000000000001111110000000000000000000000000000000000000000000000000010111110000000000000011111000000000011111000000

>1SER_A

MVDLKRLRQEPEVFHRAIREKGVALDLEALLALDREVQELKKRLQEVQTERNQVAKRVPKAPPEEKEALIARGKALGEEAKRLEEALREKEARLEALLLQVPLPPWPGAPVGGEEANREIKRVGGPPEFSFPPLDHVALMEKNGWWEPRISQVSGSRSYALKGDLALYELALLRFAMDFMARRGFLPMTLPSYAREKAFLGTGHFPAYRDQVWAIAETDLYLTGTAEVVLNALHSGEILPYEALPLRYAGYAPAFRSEAGSFGKDVRGLMRVHQFHKVEQYVLTEASLEASDRAFQELLENAEEILRLLELPYRLVEVATGDMGPGKWRQVDIEVYLPSEGRYRETHSCSALLDWQARRANLRYRDPEGRVRYAYTLNNTALATPRILAMLLENHQLQDGRVRVPQALIPYMGKEVLEPCG

0000000000000000000000000000000000000000000000000000000000000000000000000000000000000000000000000000000000000000000000000000000000000000000000000000001000110000000000000000000000000000000000000000000000000000000000000000000000000000000000000000000000000000000000010010000000000000000000000000000000000000000000000000000000000000000000000000000000000000000000000000000000000000000000000000000000000000000000000000000000000

>1SI3_A

GSHAQPVIEFCEVLDIRNIDEQPKPLTDSQRVRFTKEIKGLKVEVTHCGQKRKYRVCNVTRRPASHQTFPLQLESGQTVECTVAQYFKQKYNLQLKYPHLPCLQVGQEQKHTYLPLEVCNIVAGQRCIKKLTDNQTSTIKATARS

0000000000000000000000000000000000000000000000000001011000000000000010100000000000000110011000000000000000000111110000000000110000000000000000000

>1UN6_B

MYVCHFENCGKAFKKHNQLKVHQFSHTQQLPYECPHEGCDKRFSLPSRLKRHEKVHAGYPCKKDDSCSFVGKTWTLYLKHVAECHQD

010000000000011110010000000000000000000010000011011101100010000000000000111001000000000

>1URN_A

AVPETRPNHTIYINNLNEKIKKDELKKSLHAIFSRFGQILDILVSRSLKMRGQAFVIFKEVSSATNALRSMQGFPFYDKPMRIQYAKTDSDIIAKMK

0001000001010110010010000000000000000000001001011111101000000000000000000000001000011111111000000

>1UVJ_A

PRRAPAFPLSDIKAQMLFANNIKAQQASKRSFKEGAIETYEGLLSVDPRFLSFKNELSRYLTDHFPANVDEYGRVYGNGVRTNFFGMRHMNGFPMIPATWPLASNLKKRADADLADGPVSERDNLLFRAAVRLMFSDLEPVPLKIRKGSSTCIPYFSNDMGTKIEIAERALEKAEEAGNLMLQGKFDDAYQLHQMGGAYYVVYRAQSTDAITLDPKTGKFVSKDRMVADFEYAVTGGEQGSLFAASKDASRLKEQYGIDVPDGFFCERRRTAMGGPFALNAPIMAVAQPVRNKIYSKYAYTFHHTTRLNKEEKVKEWSLCVATDVSDHDTFWPGWLRDLICDELLNMGYAPWWVKLFETSLKLPVYVGAPAPEQGHTLLGDPSNPDLEVGLSSGQGATDLMGTLLMSITYLVMQLDHTAPHLNSRIKDMPSACRFLDSYWQGHEEIRQISKSDDAMLGWTKGRALVGGHRLFEMLKEGKVNPSPYMKISYEHGGAFLGDILLYDSRREPGSAIFVGNINSMLNNQFSPEYGVQSGVRDRSKRKRPFPGLAWASMKDTYGACPIYSDVLEAIERCWWNAFGESYRAYREDMLKRDTLELSRYVASMARQAGLAELTPIDLEVLADPNKLQYKWTEADVSANIHEVLMHGVSVEKTERFLRSVMPR

0000000000000000000000100010010000000000000000000000000000000000000000000000000000000000000000000000000000000000000000000000000000000000000000000000110100010000000000000000000000000000000000000000000101010000000000000000000000000000000000000000000000000000000000000000000111100000000100000010001000000000000000000000000000000000000000000000000000000000000000000000000000000000000000000000000011110010000000000000000000000000000000000000000000000000001000000000000000000000000000000000000000000000000000000000000000000000000000000000000000000010000000000000000000000000000000000000000000000000000000000000000000000000000000000101110111000000000001000000000000000000

>2A8V_A

MNLTELKNTPVSELITLGENMGLENLARMRKQDIIFAILKQHAKSGEDIFGDGVLEILQDGFGFLRSADSSYLAGPDDIYVSPSQIRRFNLRTGDTISGKIRPPKEGERYFALLKVNE

0000000000000000000000000000000000000000000000000000000001000101010000000100010100000000000000000000000000011100000000

>2BBV_C

MVRNNNRRRQRTQRIVTTTTQTAPVPQQNVPKQPRRRRNRARRNRRQGRAMNMGALTRLSQPGLAFLKCAFAPPDFNTDPGKGIPDRFEGKVVTRKDVLNQSINFTANRDTFILIAPTPGVAYWVADVPAGTFPISTTTFNAVNFPGFNSMFGNAAASRSDQVSSFRYASMNVGIYPTSNLMQFAGSITVWKCPVKLSNVQFPVATTPATSALVHTLVGLDGVLAVGPDNFSESFIKGVFSQSVCNEPDFEFSDILEGIQTLPPANVTVATSGQPFNLAAGAEAVSGIVGWGNMDTIVIRVSAPTGAVNSAILKTWACLEYRPNPNAMLYQFGHDSPPCDEVALQEYRTVARSLPVAVIAAQN

000000000000000000000000000000000000000000000000000000110000100100010000000000000000000000000000000000000000000000000000000000000000000000000000000000000000000000000000000000000000000000000000000000000000000000000000000000000000000000000000000000000000000000000000000000000000000000000000000000000000000000000000000000000000000000000000000000000000000000000000000

>2BBV_F

ASMWERVKSIIKSSLAMASNVPGPIGIAASGLSGLSALFEGFGF

00000001000100000000000000000000000000000000

>2FMT_A

SESLRIIFAGTPDFAARHLDALLSSGHNVVGVFTQPDRPAGRGKKLMPSPVKVLAEEKGLPVFQPVSLRPQENQQLVAELQADVMVVVAYGLILPKAVLEMPRLGCINVHGSLLPRWRGAAPIQRSLWAGDAETGVTIMQMDVGLDTGDMLYKLSCPITAEDTSGTLYDKLAELGPQGLITTLKQLADGTAKPEVQDETLVTYAEKLSKEEARIDWSLSAAQLERCIRAFNPWPMSWLEIEGQPVKVWKASVIDTATNAAPGTILEANKQGIQVATGDGILNLLSLQPAGKKAMSAQDLLNSRREWFVPGNRLV

00000000001100000000000000000000001001111101000000000000000000000000000000000000000000001110000000000000000000000000000110000000000000000000000000000000000000000000000000000000000000000000000000000000000001101000000000000000000000001000000000010100000000000000000000000000000000000000001011110000000000000000000000
