# Supplementary material for: Predicting RNA-binding sites of proteins using support vector machines and evolutionary information
Source: BMC Bioinformatics. 2008 Dec 12;9(Suppl 12):S6. doi: 10.1186/1471-2105-9-S12-S6 (PMC2638146; doi:10.1186/1471-2105-9-S12-S6)
Supplement: Additional file 3 — The RBP107 data set. [file 1471-2105-9-S12-S6-S3.doc]

# The RBP107 dataset

>1A1V_A

PPAVPQSFQVAHLHAPTGSGKSTKVPAAYAAQGYKVLVLNPSVAATLGFGAYMSKAHGVDPNIRTGVRTITTGSPITYSTYGKFLADGGSGGAYDIIICDECHSTDATSILGIGTVLDQAETAGARLVVLATATPPGSVTVPHPNIEEVALSTTGEIPFYGKAIPLEVIKGGRHLIFCHSKKKCDELAAKLVALGINAVAYYRGLDVSVIPTSGDVVVVATDALFTGDFDSVIDCNTVTQTVDFSLDPTFTIETTTLPQDAVSRTQRRGRTGRGKPGIYRFVAPGERPSGMFDSSVLCECYDAGAWYELTPAETTVRLRAYMNTPGLPVCQDHLEFWEGVFTGLTHIDAHFLSQTKQSGENFPYLVAYQATVCARAQAPPPSWDQMWKCLIRLKPTLHGPTPLLYRLGAVQNEVTLTHPITKYIMTCMS

000000000000000000000000000000000000000010100000000000000000000001000000000000000000000000000000000000000001000000000000000000000000000000000000000000000000000000000000000000000011100000000000000000000010000000000000000010100000000000000001000000000000010100000000000000000000000000000000000000000000000001000000000000000000000000000000000000000000000000000000100000000000000000000000000000000000000000000000000000000000000000000

>1A34_A

TGDNSNVVTMIRAGSYPKVNPTPTWVRAIPFEVSVQSGIAFKVPVGSLFSANFRTDSFTSVTVMSVRAWTQLTPPVNEYSFVRLKPLFKTGDSTEEFEGRASNINTRASVGYRIPTNLRQNTVAADNVCEVRSNCRQVALVISCCFN

100000000000000000000011111100000000000000000000000000000000000100000000000000000000000000000000000000000000000000000000000000000000000000000010000

>1A9N_A

VKLTAELIEQAAQYTNAVRDRELDLRGYKIPVIENLGATLDQFDAIDFSDNEIRKLDGFPLLRRLKTLLVNNNRICRIGEGLDQALPDLTELILTNNSLVELGDLDPLASLKSLTYLCILRNPVTNKKHYRLYVIYKVPQVRVLDFQKVKLKERQEAEKMFK

000000000000000000000000000000000000000000000000000000000000000000000000000000000000000000000000000000000000000000000000000000000000000000000000000100000000000000

>1APG_A

IFPKQYPIINFTTAGATVQSYTNFIRAVRGRLTTGADVRHEIPVLPNRVGLPINQRFILVELSNHAELSVTLALDVTNAYVVGYRAGNSAYFFHPDNQEDAEAITHLFTDVQNRYTFAFGGNYDRLEQLAGNLRENIELGNGPLEEAISALYYYSTGGTQLPTLARSFIICIQMISEAARFQYIEGEMRTRIRYNRRSAPDPSVITLENSWGRLSTAIQESNQGAFASPIQLQRRNGSKFSVYDVSILIPIIALMVYRCAPPPSSQF

000000000000000000000000000000000000000000000000000000000000000000000000000000010000000000000000000000000000000000000000011100000000000000000000000000000000000000000000000100000000000000000000000000000000000010000000000000000000000000000000000000000000000000000000000

>1AQ3_A

ASNFTQFVLVDNGGTGDVTVAPSNFANGVAEWISSNSRSQAYKVTCSVRQSSAQNRKYSIKVEVPKVATQTVGGVELPVAAWRSYLNMELTIPIFATNSDCELIVKAMQGLLKDGNPIPSAIAANSGIY

000000000000000000000000000000000000000000100000100000000010001000000000000000000000000000000000000000000000000000000000000000000

>1ASY_A

EDTAKDNYGKLPLIQSRDSDRTGQKRVKFVDLDEAKDSDKEVLFRARVHNTRQQGATLAFLTLRQQASLIQGLVKANKEGTISKNMVKWAGSLNLESIVLVRGIVKKVDEPIKSATVQNLEIHITKIYTISETPEALPILLEDASRSEAEAEAAGLPVVNLDTRLDYRVIDLRTVTNQAIFRIQAGVCELFREYLATKKFTEVHTPKLLGAPSEGGSSVFEVTYFKGKAYLAQSPQFNKQQLIVADFERVYEIGPVFRAENSNTHRHMTEFTGLDMEMAFEEHYHEVLDTLSELFVFIFSELPKRFAHEIELVRKQYPVEEFKLPKDGKMVRLTYKEGIEMLRAAGKEIGDFEDLSTENEKFLGKLVRDKYDTDFYILDKFPLEIRPFYTMPDPANPKYSNSYDFFMRGEEILSGAQRIHDHALLQERMKAHGLSPEDPGLKDYCDGFSYGCPPHAGGGIGLERVVMFYLDLKNIRRASLFPRDPKRLRP

0000000000000000000000000000000000000000000000000101110001010000000000001010000000000001000000000000000000000100000000001000000000000010010000100000000001001000011000000000000000000000000000000000000000000000000000000000000000000000000000000000000000000000010111110011000000000000000000000000000000000000000000000000000000000000000000000000000000000000000010000000000000000000000000000000000000000000000000000010000000000000000000000000000000000000000000000000000100000000000000000000010000

>1BMV_2

METNLFKLSLDDVETPKGSMLDLKISQSKIALPKNTVGGTILRSDLLANFLTEGNFRASVDLQRTHRIKGMIKMVATVGIPENTGIALACAMNSSIRGRASSDIYTICSQDCELWNPACTKAMTMSFNPNPCSDAWSLEFLKRTGFHCDIICVTGWTATPMQDVQVTIDWFISSQECVPRTYCVLNPQNPFVLNRWMGKLTFPQGTSRSVKRMPLSIGGGAGAKSAILMNMPNAVLSMWRYFVGDLVFEVSKMTSPYIKCTVSFFIAFGNLADDTINFEAFPHKLVQFGEIQEKVVLKFSQEEFLTAWSTQVRPATTLLADGCPYLYAMVHDSSVSTIPGDFVIGVKLTIIENMCAYGLNPGISGSRLLLGGTTIIPPQQ

00000000000000000000000000000000000000000000000000000000000000000000000000000000000000000000000000000000000000000000000000010100000000000000000000000000000000000000000000000000000000000000010000000000000000000000000000000000000000000000000000000000000000000000000000000000000000000000000000000000000000000100000000000000000000000000000000000000000000000000000000000000000000000000

>1C0A_A

MRTEYCGQLRLSHVGQQVTLCGWVNRRRDLGSLIFIDMRDREGIVQVFFDPDRADALKLASELRNEFCIQVTGTVRARDEKNINRDMATGEIEVLASSLTIINRADVLPLDSNHVNTEEARLKYRYLDLRRPEMAQRLKTRAKITSLVRRFMDDHGFLDIETPMLTKATPEGARDYLVPSRVHKGKFYALPQSPQLFKQLLMMSGFDRYYQIVKCFRDEDLRADRQPEFTQIDVETSFMTAPQVREVMEALVRHLWLEVKGVDLGDFPVMTFAEAERRYGSDKPDLRNPMELTDVADLLKSVEFAVFAGPANDPKGRVAALRVPGGASLTRKQIDEYGNFVKIYGAKGLAYIKVNERAKGLEGINSPVAKFLNAEIIEDILDRTAAQDGDMIFFGADNKKIVADAMGALRLKVGKDLGLTDESKWAPLWVIDFPMFEDDGEGGLTAMHHPFTSPKDMTAAELKAAPENAVANAYDMVINGYEVGGGSVRIHNGDMQQTVFGILGINEEEQREKFGFLLDALKYGTPPHAGLAFGLDRLTMLLTGTDNIRDVIAFPKTTAAACLMTEAPSFANPTALAELSIQVVK

000000000000000000000000010111110010000000000100001000000000000100000000000001000100000000000000000000000001101011010011000000000000000000000000000000000000000000000000100110000000000000000000000000000000000000000000001101101000000000000000000000000000000000000000000000000000000000000000000000000000000000000000000000000000000000000000000000000000000000000000000000000000000000000000000000000000000000000000000000000000000000000000000000000000000100000000000000000000000000000000000000000000000000000000000000000000000000000000000000000000000000001000000011000000000000000000000000000

>1C9S_A

TNSDFVVIKALEDGVNVIGLTRGADTRFHHSEKLDKGEVLIAQFTEHTSAIKVRGKAYIQTRHGVIESEG

0000000000000101000000000101001110000000000000000001010000000000000000

>1CVJ_A

ASLYVGDLHPDVTEAMLYEKFSPAGPILSIRVCRDMITRRSLGYAYVNFQQPADAERALDTMNFDVIKGKPVRIMWSQRDPSLRKSGVGNIFIKNLDKSIDNKALYDTFSAFGNILSCKVVCDENGSKGYGFVHFETQEAAERAIEKMNGMLLNDRKVFVGRFKSRKER

0101000000000100000000000000001000110000010100000000000000000000000000001010001000010000010101100000000000000000000010000000000101010100000000000000000000000010001101001

>1CWP_A

KAIKAWTGYSVSKWTASCAAAEAKVTSAITISLPNELSSERNKQLKVGRVLLWLGLLPSVSGTVKSCVTETQTTAAASFQVALAVADNSKDVVAAMYPEAFKGITLEQLAADLTIYLYSSAALTEGDVIVHLEVEHVRPTFDDSFTPVY

00000010000000000000000000000000000000000000000000000000000000000000000000000000000000000000000000000000000000000000000000000000000000000000000000000

>1DDL_A

MEQDKILAHQASLNTKPSLLPPPVGNPPPVISYPFQITLASLGTEDAADSVSIASNSVLATYTALYRHAQLKHLKATIHPTYMAPKYPTSVALVWVPANSTATSTQVLDTYGGLHFCIGGSVNSVKPIDVEANLTNLNPIIKASTTFTDTPKLLYYSKAQATAPTSPTCYLTIQGQIELSSPLLQASSSS

0000000000000000000000001100000000000000000000000000000000000000000000000000000000000000000010000000000000000011000000000000000000101001000000000000000000000000000000000000000000000000000000

>1DFU_P

MFTINAEVRKEQGKGASRRLRAANKFPAIIYGGKEAPLAIELDHDKVMNMQAKAEFYSEVLTIVVDGKEIKVKAQDVQRHPYKPKLQHIDFVRA

0000000010011100111010000000001000001000000000000000000000000000000000000010010000000001000000

>1DI2_A

MMPVGSLQELAVQKGWRLPEYTVAQESGPPHKREFTITCRVETFVETGSSGTSKQVAKRVAAEKLLTKFKT

00000000000000000000000000000110001000000000000000001100000000000000000

>1E7K_A

ADVNPKAYPLADAHLTKKLLDLVQQSCNYKQLRKGANEATKTLNRGISEFIVMAADAEPLEIILHLPLLCEDKNVPYVFVRSKQALGRACGVSRPVIACSVTIKEGSQLKQQIQSIQQSIERLLV

00000000000000000000000000000000011111001000100000000000010000000000000000000000000000000000110110000000000000000000000000000

>1E8O_A

PQYQTWEEFSRAAEKLYLADPMKARVVLKYRHSDGNLCVKVTDDLVCLVYKTDQAQDVKKIEKFHSQLMRLMVA

00000000000000000000000000000000000000000000000000000000001000100000010000

>1E8O_B

VLLESEQFLTELTRLFQKCRTSGSVYITLKKYDNKCLLRATDGKKKISTVVSSKEVNKFQMAYSNLLRANMDGLK

000000000000000000000000000000000000000000000000000010001001000000000000000

>1EC6_A

MKELVEIAVPENLVGAILGKGGKTLVEYQELTGARIQISKKGEFLPGTRNRRVTITGSPAATQAAQYLISQRVTYEQGVRASNPQKV

000000000011011001101000000000000000110110000000001000000000000000000000000000010000100

>1F8V_A

SKFWEGVLRVLNQISGTLSVI

000000000000100000000

>1FFY_A

MDYEKTLLMPKTDFPMRGGLPNKEPQIQEKWDAEDQYHKALEKNKGNETFILHDGPPYANGNLHMGHALNKILKDFIVRYKTMQGFYAPYVPGWDTHGLPIEQALTKKGVDRKKMSTAEFREKCKEFALEQIELQKKDFRRLGVRGDFNDPYITLKPEYEAAQIRIFGEMADKGLIYKGKKPVYWSPSSESSLAEAEIEYHDKRSASIYVAFNVKDDKGVVDADAKFIIWTTTPWTIPSNVAITVHPELKYGQYNVNGEKYIIAEALSDAVAEALDWDKASIKLEKEYTGKELEWVVAQHPFLDRESLVINGDHVTTDAGTGCVHTAPGHGEDDYIVGQQYELPVISPIDDKGVFTEEGGQFEGMFYDKANKAVTDLLTEKGALLKLDFITHSYPHDWRTKKPVIFRATPQWFASISKVRQDILDAIENTNFKVNWGKTRIYNMVRDRGEWVISRQRVWGVPLPVFYAENGEIIMTKETVNHVADLFAEHGSNIWFEREAKDLLPEGFTHPGSPNGTFTKETDIMDVWFDSGSSHRGVLETRPELSFPADMYLEGSDQYRGWFNSSITTSVATRGVSPYKFLLSHGFVMDGEGKKMSKSLGNVIVPDQVVKQKGADIARLWVSSTDYLADVRISDEILKQTSDDYRKIRNTLRFMLGNINDFNPDTDSIPESELLEVDRYLLNRLREFTASTINNYENFDYLNIYQEVQNFINVELSNFYLDYGKDILYIEQRDSHIRRSMQTVLYQILVDMTKLLAPILVHTAEEVWSHTPHVKEESVHLADMPKVVEVDQALLDKWRTFMNLRDDVNRALETARNEKVIGKSLEAKVTIASNDKFNASEFLTSFDALHQLFIVSQVKVVDKLDDQATAYEHGDIVIEHADGEKCERCWNYSEDLGAVDELTHLCPRCQQVVKSLV

00010000000000011000000000000000000000000000000000000000000000000000000000000000000000000000000000000000000000000000000000000000000000000000000000000000000000000000000000000000000000000000000000000000000000000000000000000000000000000000000000000000000000000000000000000000000000000000000000000000000000000000000000100000000000000000100000000000000000000000000000000000000000000000000000000000000000000000000000000000000000000000000000010001001000000000000000000000000000000000000000000000000000000000000000000000000000000000000000000000000000000000000000010001000000000000000000000000000010001010000000000000000000000000000101000101000100010000001001001100000000000000000000000000000000000000000000000100010011001000110000001000100000000000000000000000000000000000000000000000000000000000000000000000000010000000100100100010000000000000000000000000000000000000000000000000000000000000001000000000000000000000000000000

>1FKA_O

PITKEEKQKVIQEFARFPGDTGSTEVQVALLTLRINRLSEHLKVHKKDHHSHRGLLMMVGQRRRLLRYLQREDPERYREIVEKLGLRG

1000000000000000000011000000000000000000000000111110000000000001000000000000000000000000

>1G59_A

MVVTRIAPSPTGDPHVGTAYIALFNYAWARRNGGRFIVRIEDTDRARYVPGAEERILAALKWLGLSYDEGPDVAAPTGPYRQSERLPLYQKYAEELLKRGWAYRAFETPEELEQIRKEKGGYDGRARNIPPEEAEERARRGEPHVIRLKVPRPGTTEVKDELRGVVVYDNQEIPDVVLLKSDGYPTYHLANVVDDHLMGVTDVIRAEEWLVSTPIHVLLYRAFGWEAPRFYHMPLLRNPDKTKISKRKSHTSLDWYKAEGFLPEALRNYLCLMGFSMPDGREIFTLEEFIQAFTWERVSLGGPVFDLEKLRWMNGKYIREVLSLEEVAERVKPFLREAGLSWESEAYLRRAVELMRPRFDTLKEFPEKARYLFTEDYPVSEKAQRKLEEGLPLLKELYPRLRAQEEWTEAALEALLRGFAAEKGVKLGQVAQPLRAALTGSLETPGLFEILALLGKERALRRLERALA

000000000000000000000000000000000000000000010110000000000000000000000000000000000000000000000000000000000010000000010000000000000000000000000000100000000000000000100100000100001001110000000000000000000000001000000000000000000000000000001000001000000000000000000000000000010101000001000000000000001010011100001001000000100000000000000000000000000000000000001100000000000000000000000000000000000000000000000000000000001000000000000001001000000011001000000000000000000000

>1GAX_A

MDLPKAYDPKSVEPKWAEKWAKNPFVANPKSGKPPFVIFMPPPNVTGSLHMGHALDNSLQDALIRYKRMRGFEAVWLPGTDHAGIATQVVVERLLLKEGKTRHDLGREKFLERVWQWKEESGGTILKQLKRLGASADWSREAFTMDEKRSRAVRYAFSRYYHEGLAYRAPRLVNWCPRCETTLSDLEVETEPTPGKLYTLRYEVEGGGFIEIATVRPETVFADQAIAVHPEDERYRHLLGKRARIPLTEVWIPILADPAVEKDFGTGALKVTPAHDPLDYEIGERHGLKPVSVINLEGRMEGERVPEALRGLDRFEARRKAVELFREAGHLVKEEDYTIALATCSRCGTPIEYAIFPQWWLRMRPLAEEVLKGLRRGDIAFVPERWKKVNMDWLENVKDWNISRQLWWGHQIPAWYCEDCQAVNVPRPERYLEDPTSCEACGSPRLKRDEDVFDTWFSSALWPLSTLGWPEETEDLKAFYPGDVLVTGYDILFLWVSRMEVSGYHFMGERPFKTVLLHGLVLDEKGQKMSKSKGNVIDPLEMVERYGADALRFALIYLATGGQDIRLDLRWLEMARNFANKLYNAARFVLLSREGFQAKEDTPTLADRFMRSRLSRGVEEITALYEALDLAQAAREVYELVWSEFCDWYLEAAKPALKAGNAHTLRTLEEVLAVLLKLLHPMMPFLTSELYQALTGKEELALEAWPEPGGRDEEAERAFEALKQAVTAVRALKAEAGLPPAQEVRVYLEGETAPVEENLEVFRFLSRADLLPERPAKALVKAMPRVTARMPLEGLLDVEEWRRRQEKRLKELLALAERSQRKLASPGFREKAPKEVVEAEEARLKENLEQAERIREALSQIG

0000010000000000000000000000000000000000000000000000000000000000000000000000000000000000000000000000000000000000000000000000000000000000000000000000000000000000000000000000000000000000000000000000000000000000000010000000000000000000000000000000000000000000000000010000000000000100000000000000000000000000000000000000000000000000000000001000000000000000000000000000000000000000000000001000000000000000000000000000000000000000000000000000000000000000000000000000000000000000000000000000000000000000000000000000000000000000000000000000000000000000000000000000000100100101011000011001100110000000000000000000000000000000000000000000000000100000010001000010010000000000000000000000000000000000000000000000000000000000000000000000001000000000000000000000000000000000000000000000000000000000000000000000000000000010000000000100000001000000100000000010000000000000000000

>1H38_A

NTINIAKNDFSDIELAAIPFNTLADHYGERLAREQLALEHESYEMGEARFRKMFERQLKAGEVADNAAAKPLITTLLPKMIARINDWFEEVKAKRGKRPTAFQFLQEIKPEAVAYITIKTTLACLTSADNTTVQAVASAIGRAIEDEARFGRIRDLEAKHFKKNVEEQLNKRVGHVYKKAFMQVVEADMLSKGLLGGEAWSSWHKEDSIHVGVRCIEMLIESTGMVSLHRQNAGVVGQDSETIELAPEYAEAIATRAGALAGISPMFQPCVVPPKPWTGITGGGYWANGRRPLALVRTHSKKALMRYEDVYMPEVYKAINIAQNTAWKINKKVLAVANVITKWKHCPVEDIPAIEALTAWKRAAAAVYRKDKARKSRRISLEFMLEQANKFANHKAIWFPYNMDWRGRVYAVSMFNPQGNDMTKGLLTLAKGKPIGKEGYYWLKIHGANCAGVDKVPFPERIKFIEENHENIMACAKSPLENTWWAEQDSPFCFLAFCFEYAGVQHHGLSYNCSLPLAFDGSCSGIQHFSAMLRDEVGGRAVNLLPSETVQDIYGIVAKKVNEILQADAINGTDNEVVTVTDENTGEISEKVKLGTKALAGQWLAYGVTRSVTKRSVMTLAYGSKEFGFRQQVLEDTIQPAIDSGKGLMFTQPNQAAGYMAKLIWESVSVTVVAAVEAMNWLKSAAKLLAAEVKDKKTGEILRKRCAVHWVTPDGFPVWQEYKKPIQTRLNLMFLGQFRDSEIDAHKQESGIAPNFVHSQDGSHLRKTVVWAHEKYGIESFALIHDSFGTIPADAANLFKAVRETMVDTYESCDVLADFYDQFADQLHESQLDKMPALPAKGNLNLRDILESDFAFA

00000000000000000000000000000000000000000000000000000000000000000000000000000000000000000000000000000000000000000000000000000000000000000000000000000000000000100000000000000000000000000000000000000000000000000000000000000000000000000000000000000000000000000000000000000000000000000000000000000000000000000000000000000000000000000000000000000000000000000000000000000000000000000000000000000000000000000000000000000000000000000000000000000000000000000000000000000000000000000000000000000000000000000000000000000000000000000000000000000000000000000000000000000000000000000000000000000000000000000000000000000000000000000000000000100000000000000000000000000000000000000000000000000000000000100000000000000000000000000000000000000000000000000000000011000000000000000000000000000000000000000000000000000000000000000000000000000000000000000000000000000000000000000

>1HC8_A

TFITKTPPAAVLLKKAAGIESGSGEPNRNKVATIKRDKVREIAELKMPDLNAASIEAAMRMIEGTARSMGIVVE

00000001000001000001111100110000000000000000010010100000100110011011100000

>1HQ1_A

GFDLNDFLEQLRQDDKVLVRMEAIINSMTMKERAKPEIIKGSRKRRIAAGSGMQVQDVNRLLKQFDDMQRMMKKMK

0000000000000000001100100111001010000000011001000101100000000000000000000000

>1I6U_A

SLMDPLANALNHISNCERVGKKVVYIKPASKLIGRVLKVQDNGYIGEFEFIEDGRAGIFKVELIGKINKCGAIKPRFPVKKFGYEKFEKRYLPARDFGILIVSTTQGVSHEEAKKRGLGGRLLAYVY

0000100000000000000000000000011000100000000000000000001000000000000000000001010110000000000000000000001111000000000000101000000

>1JBR_A

ATWTCINQQLNPKTNKWEDKRLLYSQAKAESNSHHAPLSDGKTGSSYPHWFTNGYDGNGKLIKGRTPIKFGKADCDRPPKHSQNGMGKDDHYLLEFPTFPDGHDYKFDSKKPKEDPGPARVIYTYPNKVFCGIVAHQRGNQGDLRLCSH

00000000000000000000000000000000000000000000000000010000000000001000000000000000000000000000000000000000000001101000000000000000000000000000001000000

>1JID_A

AARSPADQDRFICIYPAYLNNKKTIAEGRRIPISKAVENPTATEIQDVCSAVGLNVFLEKNKMYSREWNRDVQYRGRVRVQLKQEDGSLCLVQFPSRKSVMLYAAEMIPKLKTR

000000000100100001000001000010011000000000000000000000000000000101000000000000100000000000000000100000000000000000

>1JJ2_Y

RTGRFGPRYGLKIRVRVADVEIKHKKKHKCPVCGFKKLKRAGTGIWMCGHCGYKIAGGCYQPETVAGKAVMKA

1011011111111100000000001000000000001001001000000000000000000000000000000

>1JJ2_z

TGAGTPSQGKKNTTTHTKCRRCGEKSYHTKKKVCSSCGFGKSAKRRDYEWQSKAGE

11111111111111111101110011111111001000001011110111101100

>1JJ2_A

GRRIQGQRRGRGTSTFRAPSHRYKADLEHRKVEDGDVIAGTVVDIEHDPARSAPVAAVEFEDGDRRLILAPEGVGVGDELQVGVDAEIAPGNTLPLAEIPEGVPVCNVESSPGDGGKFARASGVNAQLLTHDRNVAVVKLPSGEMKRLDPQCRATIGVVGGGGRTDKPFVKAGNKHHKMKARGTKWPNVRGVAMNAVDHPFGGGGRQHPGKPKSISRNAPPGRKVGDIASKRTGRGG

111001111111111111011001000000100000000000000010001100000000000000000000000000000000000000000000000000000000011010001111100000000000000000001100010000000000000000111011111110101101011111111110101001100011011111111101110111111111111111110

>1JJ2_1

GKKSKATKKRLAKLDNQNSRVPAWVMLKTDERNHKRRHWRRNDTDE

1110110111001001101000011011110111001111011000

>1JJ2_B

PQPSRPRKGSLGFGPRKRSTSETPRFNSWPSDDGQPGVQGFAGYKAGMTHVVLVNDEPNSPREGMEETVPVTVIETPPMRAVALRAYEDTPYGQRPLTEVWTDEFHSELDRTLDVPEDHDPDAAEEQIRDAHEAGDLGDLRLITHTVPDAVPSVPKKKPDVMETRVGGGSVSDRLDHALDIVEDGGEHAMNDIFRAGEYADVAGVTKGKGTQGPVKRWGVQKRKGKHARQGWRRRIGNLGPWNPSRVRSTVPQQGQTGYHQRTELNKRLIDIGEGDEPTVDGGFVNYGEVDGPYTLVKGSVPGPDKRLVRFRPAVRPNDQPRLDPEVRYVSNESNQG

1101111111111101110101001011000000000000000010010000000000100000000001000000000000001010000101011110000000000011010110100000000000000000000010100000000000011100101000000000000000000000000000000000000000000111111110011000111111101111111011101111111111001101000011111110000000000000110011010000000000011110011000010001010000000000000001111

>1JJ2_2

MQMPRRFNTYCPHCNEHQEHEVEKVRSGRQTGMKWIDRQRERNSGIGNDGKFSKVPGGDKPTKKTDLKYRCGECGKAHLREGWRAGRLEFQE

10000010110000111000000000111110001001100110011101101100011101111001000000011011010101000010

>1JJ2_C

MQATIYDLDGNTDGEVDLPDVFETPVRSDLIGKAVRAAQANRKQDYGSDEYAGLRTPAESFGSGRGQAHVPKLDGRARRVPQAVKGRSAHPPKTEKDRSLDLNDKERQLAVRSALAATADADLVADRGHEFDRDEVPVVVSDDFEDLVKTQEVVSLLEALDVHADIDRADETKIKAGQGSARGRKYRRPASILFVTSDEPSTAARNLAGADVATASEVNTEDLAPGGAPGRLTVFTESALAEVAER

000000000000000000000000001001000001011011111111100110111001001101011110110111011111101111011111100010111110000000000000000000110000000000000000000111100000000000000001010011111110110101100000000000000100110000001001101001000100000000000000000001

>1JJ2_D

FHEMREPRIEKVVVHMGIGHANAEDILGEITGQMPVRTKAKRTVGEFDIREGDPIGAKVTLRDEMAEEFLQTALPLAELATSQFDDTGNFSFGLDVTVNLVRPGYRVAKRDKASRSIPTKHRLNPADAVAFIESTYDVEV

10110001001010100000000000000000000111110100010000000000010101000000000000000000011010100010000000100011111011100011011111000000000000000000

>1JJ2_E

PRVELEIPEDVDAEQDHLDITVEGDNGSVTRRLWYPDIDVSVDGDTVVIESDEDNAKTMSTIGTFQSHIENMFHGVTEGWEYGMEVFYSHFPMQVNVEGDEVVIENFLGEKAPRRTTIHGDTDVEIDGEELTVSGPDIEAVGQTAADIEQLTRINDKDVRVFQDGVYITRKP

0000000000000000000000000000000001000000000000000000001000010011001100100000000001000000010000000000000000000111000000000000000000000000001000100010010010001101010000000000

>1JJ2_I

AEFDADVIVDARDCIMGRVASQVAEQALDGETVAVVNAERAVITGREEQIVEKYEKRVDIGNDNGYFYPKRPDGIFKRTIRGMLPHKKQRGREAFESVRVYLGNPYDEDGEVLDGTSLDRLSNIKFVTLGEISETLGANKTW

0000000000000010110010010001000000000000000101000000100110010101111001100000111011101111010100010010000000000000000000000000100000000000100000

>1JJ2_J

MEALGADVTQGLEKGSLITCADNTGARELKVISVHGYSGTKNRHPKAGLGDKITVSVTKGTPEMRRQVLEAVVVRQRKPIRRPDGTRVKFEDNAAVIVDENEDPRGTELKGPIAREVAQRFGSVASAATMIV

101000001110100110000000000001011010111111111000000001010000000001000000000011001000001000001000000000000000000000000000000000000000

>1JJ2_K

TSKKKRQRGSRTHGGGSHKNRRGAGHRGGRGDAGRDKHEFHNHEPLGKSGFKRPQKVQEEAATIDVREIDENVTLLAADDVAEFRVDVRDVVEEADDADYVKVLGAGQVRHELTLIADDFSEGAREKVEGAGGSVELTDLGEERQA

10111101101111011111111111111111011011101000011101111011100000101000011000000000000000000000000000000101010100000000000011000010000000000000000001

>1JJ2_L

RSAYSYIREAWKRPKEGQIAELMWHRMQEWRNEPAVVRIERPTRLDRARSLGYKAKQGIIVVRVAIRKGSSRRTRFNKGRRSKRMMVNRITRKKNIQRIAEERANRKFPNLRVLNSYSVGEDGRHKWHEVILIDPDHPAIKSDDQLSWISRTRHRLRTFRGLTSAGRRCRGLRGQGKGSEKVRPSLRVNGAKA

0101000100010110000000010000001001000100100110101110011110000000001111011111111111111011101111100110001001111100001000001011010000000001110000001001000110111011000100100100101111011011111101010

>1JJ2_M

ATGPRYKVPMRRRREARTDYHQRLRLLKSGKPRLVARKSNKHVRAQLVTLGPNGDDTLASAHSSDLAEYGWEAPTGNMPSAYLTGLLAGLRAQEAGVEEAVLDIGLNSPTPGSKVFAIQEGAIDAGLDIPHNDDVLADWQRTRGAHIAEYDEQLEEPLYSGDFDAADLPEHFDELRETLLDGDIEL

101111010011110011010010100110001000111111010000000011111001000000000000000111000000000000000000000010000001111001000000000000000101001000001000011001000000001101000000000000000000000000

>1JJ2_N

SKTNPRLSSLIADLKSAARSSGGAVWGDVAERLEKPRRTHAEVNLGRIERYAQEDETVVVPGKVLGSGVLQKDVTVAAVDFSGTAETKIDQVGEAVSLEQAIENNPEGSHVRVIR

1011000000010011001000011001000100011100010101100100000000000010110100000000000001110000000000000000000000011101101

>1JJ2_O

TDLSAQKRLAADVLDVGKNRVWFNPERQGDIADAITREDVRELVDEGAIQAKDKKGNSRGRARERQKKRAKGHQKGAGSRKGKAGARQNSKEDWESRIRAQRTKLRELRDEGTLSSSQYRDLYDKAGGGEFDSVADLERYIDA

11100011000000001011101000010000000010001000000000010111111010101101101011111101111011110010011100110100000010000001101100111001010010000000000

>1JJ2_P

PSSNGPLEGTRGKLKNKPRDRGTSPPQRAVEEFDDGEKVHLKIDPSVPNGRFHPRFDGQTGTVEGKQGDAYKVDIVDGGKEKTIIVTAAHLRRQE

11111111001011100010111010000000000001010100110110000010011100000011000000001011011010000000011

>1JJ2_Q

GISYSVEADPDTTAKAMLRERQMSFKHSKAIAREIKGKTAGEAVDYLEAVIEGDQPVPFKQHNSGVGHKSKVDGWDAGRYPEKASKAFLDLLENAVGNADHQGFDGEAMTIKHVAAHKVGEQQGRKPRAMGRASAWNSPQVDVELILEEP

111110000000000010100101110010001001000000000000000000000001010110011110011100110110000000000100010011000000000010101010010001111111010110100000000000

>1JJ2_R

SWDVIKHPHVTEKAMNDMDFQNKLQFAVDDRASKGEVADAVEEQYDVTVEQVNTQNTMDGEKKAVVRLSEDDDAQEVASRI

100000001011100000001010000000001100000000000000000011110100011000100000000000000

>1JJ2_S

SKQPDKQRKSQRRAPLHERHKQVRATLSADLREEYGQRNVRVNAGDTVEVLRGDFAGEEGEVINVDLDKAVIHVEDVTLEKTDGEEVPRPLDTSNVRVTDLDLEDEKREARLESEDDSA

11001111101110111001100001000001000001101011000000011100000000000010100000000010010000001101011010000000101000100001000

>1JJ2_T

RECDYCGTDIEPGTGTMFVHKDGATTHFCSSKCENNADLGREARNLEWTDTAR

00000000000001000000000000000011011000101000001100001

>1JJ2_U

TVLHVQEIRDMTPAEREAELDDLKTELLNARAVQAAGGAPENPGRIKELRKAIARIKTIQGEEGD

10010000100000000000000000000000010000000001000001100110110000000

>1JJ2_V

MHALVQLRGEVNMHTDIQDTLEMLNIHHVNHCTLVPETDAYRGMVAKVNDFVAFGEPSQETLETVLATRAEPLEGDADVDDEWVAEHTDYDDISGLAFALLSEETTLREQGLSPTLRLHPPRGGHDGVKHPVKEGGQLGKHDTEGIDDLLEAMR

0000000110110110001100100011101000000011101100100100000000000000000010000110000000000000000000000000000000010000100011111111001011101100000000000000000001

>1JJ2_W

ERVVTIPLRDARAEPNHKRADKAMILIREHLAKHFSVDEDAVRLDPSINEAAWARGRANTPSKIRVRAARFEEEGEAIVEAETE

010000001101101110001101100100001100000000110000110010101010011000000000000000000000

>1JJ2_X

LQARGLTEKTPDLSDEDARLLTQRHRVGKPQFNRQDHHKKKRVSTSWRKPRGQLSKQRRGIKGKGDTVEAGFRSPTAVRGKHPSGFEEVRVHNVDDLEGVDGDTEAVRIASKVGARKRERIEEEAEDAGIRVLNPTYVEV

00000000000000000010001111001111011101111100011110111111011111110001111111010011011000000101000000000000000101111111101000000000000011001000

>1JJ2_G

IPEWKQEEVDAIVEMIESRNTLLERALDD

01001000100000000011100010000

>1K8W_A

MDINGVLLLDKPQGMSSNDALQKVKRIYNANRAGHTGALDPLATGMLPIICLGEATKFSQYLLDSDKRYRVIARLGQRTDTSDADGQIVEERPVTFSAEQLAAALDTFRGDIEQIPSMYSALKYQGKKLYEYARQGIEVPREARPITVYELLFIRHEGNNELELEIHCSKGTYIRTIIDDLGEKLGCGAHVIYLRRLAVSKYPVERMVVTLEHLRELVEQAEQQQDIPAAEELLDPLLMPMDSPASDYPVVNLPLTSSVYFKNGNPVRTSGAPLEGLVRVTEGENGKFIGMGEIDDEGRRVAPRRLVVEY

0000000000000001010010000000000010101001100000000000000100000010000000000000000010000000000000000000000000000000000000000110110101000100000000010000000000000000000000000000100000000000000000000000000000000000000000000000000000000000000000000000000000000000000000000000000000000000000000000000000000000000000000

>1KNZ_A

TQQMAVSIINSSFEAAVVAATSALENMGIEYDYQDIYSRVKNKFDFVMDDSGVKNNPIGKAITIDQALNNKFGSAIRNRNWLADTSRPAKLDEDVNKLRMMLGIDQKMRVLNACFSVKRIPGKSSSIIKCTKLMRDKLERGEVEVDDSFVDEKM

0000000000000000000000000000000000000000000000000000001000000000000000000100010010000000000001000000000000000000000000000111110000000000000000000000000000

>1KOG_A

RDHRKIGKQLDLYHMQEEAPGMVFWHNDGWTIFRELEVFVRSKLKEYQYQEVKGPFMMDRVLWEKTGHWDNYKDAMFTTSSENREYCIKPMNCPGHVQIFNQGLKSYRDLPLRMAEFGSCHRNEPSGSLHGLMRVRGFTQDDAHIFCTEEQIRDEVNGCIRLVYDMYSTFGFEKIVVKLSTRPEKRIGSDEMWDRAEADLAVALEENNIPFEYQLGEGAFYGPKIEFTLYDCLDRAWQCGTVQLDFSLPSRLSASYVGEDNERKVPVMIHRAILGSMERFIGILTEEFAGFFPTWLAPVQVVIMNITDSQSEYVNELTQKLSNAGIRVKADLRNEKIGFKIREHTLRRVPYMLVCGDKEVESGKVAVRTRRGKDLGSMDVNEVIEKLQQEIRSRSLKQLEE

00000001000000000000000000000000000000000000000000000000000000000000000000000000000000000000000000000011011100000000000000000000000000000000000000000000000000000000000000000000000000000000000000000000000000000000000000000000000000000000000000000000000000001010100000000000000000000000000000000000000000000110000000000000000000000000010110101100000000000000010000000001000000000000000000000000000000000

>1L9A_A

MIIWPSYIDKKKSRREGRKVPEELAIEKPSLKDIEKALKKLGLEPKIYRDKRYPRQHWEIAGRVEVDYKGNKLCLLKEIAKIIKGKN

100100100000111001101100000000000000000000000000001111101000001000000011100010000000000

>1M8Y_A

GRSRLLEDFRNNRYPNLQLREIAGHIMEFSQDQHGSRFIQLKLERATPAERQLVFNEILQAAYQLMVDVFGNYVIQKFFEFGSLEQKLALAERIRGHVLSLALQMYGCRVIQKALEFIPSDQQNEMVRELDGHVLKCVKDQNGNHVVQKCIECVQPQSLQFIIDAFKGQVFALSTHPYGCRVIQRILEHCLPDQTLPILEELHQHTEQLVQDQYGNYVIQHVLEHGRPEDKSKIVAEIRGNVLVLSQHKFASNVVEKCVTHASRTERAVLIDEVCTMNDGPHSALYTMMKDQYANYVVQKMIDVAEPGQRKIVMHKIRPHIATLRKYTYGKHILAKLEKYY

00000000000000000000000000000000100000000000000000000000000000000000000110000000000000000000000000000000000110000000000000000000000000000000000010010000000000000000000000000000000000010000000000000000000000000000000110010000000000000000000000000000000110010000000000000000000000000000000000000011000000000000000000000000000000000000000000000

>1MFQ_C

KHGQFTLRDMYEQFQNIMKMGPFSQILGMIPGFNEQESMARLKKLMTIMDSMNDQELDSTDGAKVFSKQPGRIQRVARGSGVSTRDVQELLTQYTKFAQMVKKMGGIK

000000000000000000000000000000000000000000000010011110000000000000000000001000111000000000000000000000000000

>1MZP_A

MLADKESLIEALKLALSTEYNVKRNFTQSVEIILTFKGIDKKGDLKLREIVPLPKQPSKAKRVLVVPSSEQLEYAKKASPKVVITREELQKLQGQKRPVKKLARQNEWFLINQESALAGRILGPALGPRGKFPTPLPNTADISEYINRFKRSVLVKTKDQPQVQVFIGTEDKPEDLAENAIAVLNAIENKAKVETNLRNIYVKTTGKAVKVKR

000000000000000000000001110010101010000000000000000000000010000000000000000000000000000000000001100110010000000000000000000000011000000000000000000000000001000101010000000000000000000000000000000001000010110000000

>1OOA_A

GGPYLQILEQPKQRGFRFRYVCEGPSHGGLPGASSEKNKKSYPQVKICNYVGPAKVIVQLVTNGKNIHLHAHSLVGKHCEDGVCTVTAGPKDMVVGFANLGILHVTKKKVFETLEARMTEACIRGYNPGLLVHSDLAYLQAEGGGDRQLTDREKEIIRQAAVQQTKEMDLSVVRLMFTAFLPDSTGSFTRRLEPVVSDAIYDSKAPNASNLKIVRMDRTAGCVTGGEEIYLLCDKVQKDDIQIRFYEEEENGGVWEGFGDFSPTDVHRQFAIVFKTPKYKDVNITKPASVFVQLRRKSDLETSEPKPFLYYPE

0000000000000000101101101110000000000001000000000000000000000000000000000000000000000000000000000000000001110000000000000000000000000000000000000000000000000000000000000000000000000000000000000000000000011000000100000000000000000000000000000000000000000000000000000000000000000000000000000000000000000000000000000

>1P6V_A

SDKIIPIAENKEAKAKYDILETYEAGIVLKGSEVKSLREKGTVSFKDSFVRIENGEAWLYNLYIAPYKHANHDPLRKRKLLLHKREIMRLYGKVQEKGYTIIPLKLYWKNNKVKVLIALAKGKKL

00000000000000000000000100111000011000000000000000000001000000000000100000000000111111011011101100000000000001111100000000000

>1Q2R_A

RPRFSFSIAAREGKARTGTIEMKRGVIRTPAFMPVGTAATVKALKPETVRATGADIILGNTYHLMLRPGAERIAKLGGLHSFMGWDRPILTDSGGYQVMSLSSLTKQSEEGVTFKSHLDGSRHMLSPERSIEIQHLLGSDIVMAFDECTPYPATPSRAASSMERSMRWAKRSRDAFDSRKEQAENAALFGIQQGSVFENLRQQSADALAEIGFDGYAVGGLAVGEGQDEMFRVLDFSVPMLPDDKPHYLMGVGKPDDIVGAVERGIDMFDCVLPTRSGRNGQAFTWDGPINIRNARFSEDLKPLDSECHCAVCQKWSRAYIHHLIRAGEILGAMLMTEHNIAFYQQLMQKIRDSISEGRFSQFAQDFRARYFARNS

0000000000000000000000000000000000001000000000000000000000000010000000000000000000000000000000001001000000000000000000000000000000000000000000000000000000000000000000000000000000000000000000000000000000000000000000000000010000000000000000000000000000000100000000000000000000110011010000000010111000000000000000000000000000000100000000000000000000000000000000000000000000000000

>1QF6_A

PVITLPDGSQRHYDHAVSPMDVALDIGPGLAKACIAGRVNGELVDACDLIENDAQLSIITAKDEEGLEIIRHSCAHLLGHAIKQLWPHTKMAIGPVIDNGFYYDVDLDRTLTQEDVEALEKRMHELAEKNYDVIKKKVSWHEARETFANRGESYKVSILDENIAHDDKPGLYFHEEYVDMCRGPHVPNMRFCHHFKLMKTAGAYWRGDSNNKMLQRIYGTAWADKKALNAYLQRLEEAAKRDHRKIGKQLDLYHMQEEAPGMVFWHNDGWTIFRELEVFVRSKLKEYQYQEVKGPFMMDRVLWEKTGHWDNYKDAMFTTSSENREYCIKPMNCPGHVQIFNQGLKSYRDLPLRMAEFGSCHRNEPSGSLHGLMRVRGFTQDDAHIFCTEEQIRDEVNGCIRLVYDMYSTFGFEKIVVKLSTRPEKRIGSDEMWDRAEADLAVALEENNIPFEYQLGEGAFYGPKIEFTLYDCLDRAWQCGTVQLDFSLPSRLSASYVGEDNERKVPVMIHRAILGSMERFIGILTEEFAGFFPTWLAPVQVVIMNITDSQSEYVNELTQKLSNAGIRVKADLRNEKIGFKIREHTLRRVPYMLVCGDKEVESGKVAVRTRRGKDLGSMDVNEVIEKLQQEIRSRSLKQLEE

00000000000000000000000000000000000000000000000000000000000000000000000000000000000000000000000000000000000001000000000000000000000000000000000000000000000000000000000000000000000000000000000000000010110100100000100001000000000000000000000000010000000000001000000000000000000000000000000000000000000000000001000000000000000000000000000000000000000000000000000000000101000001000000000000000000000000000000000000000000000000000000000000000000000000000000000000001000000000000010000000100000000000000000000000000000000000000000000000000000000000000110000000000000000000000000010100000100000000000001000000000100000000000000000000000000000000000

>1RMV_A

SYNITNSNQYQYFAAVWAEPTPMLNQCVSALSQSYQTQAGRDTVRQQFANLLSTIVAPNQRFPDTGFRVYVNSAVIKPLYEALMKSFDTRNRIIETEEESRPSASEVANATQRVDDATVAIRSQIQLLLNELSNGHGYMNRAEFEAILPWTTAPAT

000000000000000000000000000000000000000000000000000000000000000000000000000000000000000000000000000000000000000000110010000000000000000000000000000000000000

>1RPU_A

DTREQANGERWDGGSGGITSPFKLPDESPSWTEWRLYNDENPLGFKESWGFGKVVFKRYLRYDRTEASLHRVLGSWTGDSVNYAASRFLGANQVGCTYSIRFRGVSVTISGGSRTLQHLCEMAIRSKQELLQLTPVEV

001000000100000000000000000101110110000000000101000110101010000000000000000000000000000000001100101010000100010100000000000000000000000000

>1S72_H

KPASMYRDIDKPAYTRREYITGIPGSKIAQHKMGRKQKDADDYPVQISLIVEETVQLRHGSLEASRLSANRHLIKELGEEGDYKMTLRKFPHQVLRENKDGMRAAFGKIVGTAARVQAGEQLFTAYCNVEDAEHVKEAFRRAYNKITPSCRIDSSPAGNA

1010101000111111011000000000000100010000000000000000100001001001000000100000000000000001110100011000011111100000000000000000000000000000000110111010100000000000

>1SER_B

MVDLKRLRQEPEVFHRAIREKGVALDLEALLALDREVQELKKRLQEVQTERNQVAKRVPKAPPEEKEALIARGKALGEEAKRLEEALREKEARLEALLLQVPLPPWPGAPVGGEEANREIKRVGGPPEFSFPPLDHVALMEKNGWWEPRISQVSGSRSYALKGDLALYELALLRFAMDFMARRGFLPMTLPSYAREKAFLGTGHFPAYRDQVWAIAETDLYLTGTAEVVLNALHSGEILPYEALPLRYAGYAPAFRSEAGSFGKDVRGLMRVHQFHKVEQYVLTEASLEASDRAFQELLENAEEILRLLELPYRLVEVATGDMGPGKWRQVDIEVYLPSEGRYRETHSCSALLDWQARRANLRYRDPEGRVRYAYTLNNTALATPRILAMLLENHQLQDGRVRVPQALIPYMGKEVLEPCG

0000000000000000000000000000000000000000010000000011001001000000000000000000000010000000000000000000000000000000000000000000000000000000000000000000000000000000000000000000000000000000000000000010000000000000000000000000000000000000000000000000000000000000000000000000000000000000000000000000000000000000000000000000000000000000000000000000000000000000000000000000100000000000000000000000000000000000000000000000000000000

>1SI2_A

MAQPVIEFMCEVLDIRNIDEQPKPLTDSQRVRFTKEIKGLKVEVTHCGQMKRKYRVCNVTRRPASHQTFPLQVECTVAQYFKQKYNLQLKYPHLPCLQVGQEQKHTYLPLEVCNIVAGQR

000000000000000000000000000000000000000010000100000100100000000000001000000000000000000000000000000010011000000000000010

>1TTT_A

AKGEFIRTKPHVNVGTIGHVDHGKTTLTAALTYVAAAENPNVEVKDYGDIDKAPEERARGITINTAHVEYETAKRHYSHVDCPGHADYIKNMITGAAQMDGAILVVSAADGPMPQTREHILLARQVGVPYIVVFMNKVDMVDDPELLDLVEMEVRDLLNQYEFPGDEVPVIRGSALLALEEMHKNPKTKRGENEWVDKIWELLDAIDEYIPTPVRDVDKPFLMPVEDVFTITGRGTVATGRIERGKVKVGDEVEIVGLAPETRKTVVTGVEMHRKTLQEGIAGDNVGLLLRGVSREEVERGQVLAKPGSITPHTKFEASVYILKKEEGGRHTGFFTGYRPQFYFRTTDVTGVVRLPQGVEMVMPGDNVTFTVELIKPVALEEGLRFAIREGGRTVGAGVVTKILE

000000000000000000000000000000000000000000000000000110000000001100100000000000000000000101100000000000000000000000000000000000000000000000000000000000000000000000000000000000000000000000000000000000000000000000000000000000000100000000000000000000000000000000000000000010010100000000001010000000000001000000000000000000000000000001000010010010000000010000000000000000000000000100000000000001110000000000001

>1U0B_B

MLKIFNTLTRQKEEFKPIHAGEVGMYVCGITVYDLCHIGHGRTFVAFDVVARYLRFLGYKLKYVRNITDIDDKIIKRANENGESFVAMVDRMIAEMHKDFDALNILRPDMEPRATHHIAEIIELTEQLIAKGHAYVADNGDVMFDVPTDPTYGVLSRQDLDQLQAGARVDVVDDKRNPMDFVLWKMSKEGEPSWPSPWGAGRPGWHIECSAMNCKQLGNHFDIHGGGSDLMFPHHENEIAQSTCAHDGQYVNYWMHSGMVMVDREKMSKSLGNFFTVRDVLKYYDAETVRYFLMSGHYRSQLNYSEENLKQARAALERLYTALRGTDKTVAPAGGEAFEARFIEAMDDDFNTPEAYSVLFDMAREVNRLKAEDMAAANAMASHLRKLSAVLGLLEQEPEAFLQSGAQADDSEVAEIEALIQQRLDARKAKDWAAADAARDRLNEMGIVLEDGPQGTTWRRK

00000000010000000000000000000000000000100000000000000000000000000000000000000000000000000000000000000000000000000000000000000000000000000000000000000000000001000000011000000000000000000000000000000000000000000000000000000000001000010000000000000000000000000001100000100000000000000000000000000000100010000000001000100100000000000000000000000000000000101100100100010010000000000000000000000000000000000000000000000000000000000010000000000010000000000110000000100

>1URN_A

AVPETRPNHTIYINNLNEKIKKDELKKSLHAIFSRFGQILDILVSRSLKMRGQAFVIFKEVSSATNALRSMQGFPFYDKPMRIQYAKTDSDIIAKM

000000000001011001001000000000000000000000000001101110100000000000000000000000100001000101100000

>1UVJ_A

PRRAPAFPLSDIKAQMLFANNIKAQQASKRSFKEGAIETYEGLLSVDPRFLSFKNELSRYLTDHFPANVDEYGRVYGNGVRTNFFGMRHMNGFPMIPATWPLASNLKKRADADLADGPVSERDNLLFRAAVRLMFSDLEPVPLKIRKGSSTCIPYFSNDMGTKIEIAERALEKAEEAGNLMLQGKFDDAYQLHQMGGAYYVVYRAQSTDAITLDPKTGKFVSKDRMVADFEYAVTGGEQGSLFAASKDASRLKEQYGIDVPDGFFCERRRTAMGGPFALNAPIMAVAQPVRNKIYSKYAYTFHHTTRLNKEEKVKEWSLCVATDVSDHDTFWPGWLRDLICDELLNMGYAPWWVKLFETSLKLPVYVGAPAPEQGHTLLGDPSNPDLEVGLSSGQGATDLMGTLLMSITYLVMQLDHTAPHLNSRIKDMPSACRFLDSYWQGHEEIRQISKSDDAMLGWTKGRALVGGHRLFEMLKEGKVNPSPYMKISYEHGGAFLGDILLYDSRREPGSAIFVGNINSMLNNQFSPEYGVQSGVRDRSKRKRPFPGLAWASMKDTYGACPIYSDVLEAIERCWWNAFGESYRAYREDMLKRDTLELSRYVASMARQAGLAELTPIDLEVLADPNKLQYKWTEADVSANIHEVLMHGVSVEKTERFLRSVMPR

0000000000000000000000000000010000000000000000000000000000000000000000000000000000000000000000000000000000000000000000000000000000000000000000000000110100000000000000000000000000000000000000000000000000010000000000000000000000000000000000000000000000000000000000000000000000000000000100000000000000000000000000000000000000000000000000000000000000000000000000000000000000000000000000000000000010100010000000000000000000000000000000000000000000000000000000000000000000000000000000000000000000000000000000000000000000000000000000000000000000000010000000000000000000000000000000000000000000000000000000000000000000000000000000000101100000000000000000000000000000000000

>1WMQ_A

TLHKERRIGRLSVLLLLNSTQVEELERDGWKVCLGKVGSMDAHKVIAAIETASKKSGVIQSEGYRESHALYHATMEALHGVTRGEMLLGSLLRTVGLRFAVLRGNPYESEAEGDWIAVSLYGTIGAPIKGLEHETFGVGINHI

00000000000000000000000000000000000101000000000100000000000000000000000000000000000000000000010100000000000000000000000000100111000100000000000

>1WNE_A

GLIVDTRDVEERVHVMRKTKLAPTVAHGVFNPEFGPAALSNKDPRLNEGVVLDEVIFSKHKGDTKMSAEDKALFRRCAADYASRLHSVLGTANAPLSIYEAIKGVDGLDAMEPDTAPGLPWALQGKRRGALIDFENGTVGPEVEAALKLMEKREYKFACQTFLKDEIRPMEKVRAGKTRIVDVLPVEHILYTRMMIGRFCAQMHSNNGPQIGSAVGCNPDVDWQRFGTHFAQYRNVWDVDYSAFDANHCSDAMNIMFEEVFRTEFGFHPNAEWILKTLVNTEHAYENKRITVEGGMPSGCSATSIINTILNNIYVLYALRRHYEGVELDTYTMISYGDDIVVASDYDLDFEALKPHFKSLGQTITPADKSDKGFVLGHSITDVTFLKRHFHMDYGTGFYKPVMASKTLEAILSFARRGTIQEKLISVAGLAVHSGPDEYRRLFEPFQGLFEIPSYRSLYLRWVNAVCGDAAALEHH

00000000000000001000000000000000000000000000000000000000000000000000000000000000000000000000000000000000001010000010000000000001000000000000000000000000000000000100000000000000000010000000100010000000000100000000000111100000000000000000000000000000000000000000000000000000000000000000000000000000001000000000000000000000000000000000000110000000000000000000000000000000000000000000000001100000000000000000000000000001000000100010000000000000000000000000000000000000000000000000

>1XMQ_B

VKELLEAGVHFGHERKRWNPKFARYIYAERNGIHIIDLQKTMEELERTFRFIEDLAMRGGTILFVGTKKQAQDIVRMEAERAGMPYVNQRWLGGMLTNFKTISQRVHRLEELEALFASPEIEERPKKEQVRLKHELERLQKYLSGFRLLKRLPDAIFVVDPTKEAIAVREARKLFIPVIALADTDSDPDLVDYIIPGNDDAIRSIQLILSRAVDLIIQARGGVVEPSPSYALVQ

000000000000000101010000000000000000000000000000000000000000000000000000000000000000000000001100110000001000000000000000000000100000000001000000000000000000000000000100000010000000000000000000000000000000000000000000000000000000000000

>1XMQ_C

GNKIHPIGFRLGITRDWESRWYAGKKQYRHLLLEDQRIRGLLEKELYSAGLARVDIERAADNVAVTVHVAKPGVVIGRGGERIRVLREELAKLTGKNVALNVQEVQNPNLSAPLVAQRVAEQIERRFAVRRAIKQAVQRVMESGAKGAKVIVSGRIGGAEQARTEWAAQGRVPLHTLRANIDYGFALARTTYGVLGVKAYIFLGEV

11110000000000000000000001000000000000000000000000000000000000000000000000000000000000000000000000000000000000000000000000000000000000000000000000000000101000010100000000110111100000000000101111000100000000

>1XMQ_D

GRYIGPVCRLCRREGVKLYLKGERCYSPKCAMERRPYPPGQHGQKRARRPSDYAVRLREKQKLRRIYGISERQFRNLFEEASKKKGVTGSVFLGLLESRLDNVVYRLGFAVSRRQARQLVRHGHITVNGRRVDLPSYRVRPGDEIAVAEKSRNLELIRQNLEAMKGRKVGPWLSLDVEGMKGKFLRLPDREDLALPVNEQLVIEFYSR

1000011010011000000010110000101000100001110010000010000101011000100000111011000000000000000000000000000000000001011011001101000000110111000100000000000001100000000000000000000000000000000000000000000000010001

>1XMQ_E

DFEEKMILIRRTARMQAGGRRFRFGALVVVGDRQGRVGLGFGKAPEVPLAVQKAGYYARRNMVEVPLQNGTIPHEIEVEFGASKIVLKPAAPGTGVIAGAVPRAILELAGVTDILTKELGSRNPINIAYATMEALRQLRTKADVERLRKG

000000000101111111011010000000000000000000100000000010001000000000000000000000000000000101000000110000000000000000011100111001001000000000000000000000

>1XMQ_F

MRRYEVNIVLNPNLDQSQLALEKEIIQRALENYGARVEKVEELGLRRLAYPIAKDPQGYFLWYQVEMPEDRVNDLARELRIRDNVRRVMVVKSQEPFLANA

01010000000000000000000000000000000000000000000001000100000000000000000010000000000000100111000000000

>1XMQ_G

ARRRRAEVRQLQPDLVYGDVLVTAFINKIMRDGKKNLAARIFYDACKIIQEKTGQEPLKVFKQAVENVKPRMEVRSRRVGGANYQVPMEVSPRRQQSLALRWLVQAANQRPERRAAVRIAHELMDAAEGKGGAVKKKEDVERMAEANRAYAHYRW

11101000101000000000000000111001111100011000000000000000000000000000000000100000000000000000110010001000000100001010000000000000000000000000000000000000000

>1XMQ_H

MLTDPIADMLTRIRNATRVYKESTDVPASRFKEEILRILAREGFIKGYERVDVDGKPYLRVYLKYGPRRQGPDPRPEQVIHHIRRISKPGRRVYVGVKEIPRVRRGLGIAILSTSKGVLTDREARKLGVGGELICEVW

101000010011011000101000000011000000000000000000000000010000000000000000001000000000000111110111000000000000000011100000000000000101000000

>1XMQ_I

EQYYGTGRRKEAVARVFLRPGNGKVTVNGQDFNEYFQGLVRAVAALEPLRAVDALGRFDAYITVRGGGKSGQIDAIKLGIARALVQYNPDYRAKLKPLGFLTRDARVVERKKYGKHKARRAPQYSKR

1101000111101010001000000000000000101010000000000000000000001000111111110000000001000000000100010000111001011111101110110011111

>1XMQ_J

KIRIKLRGFDHKTLDASAQKIVEAARRSGAQVSGPIPLPTRVRRFTVIRGPFKHKDSREHFELRTHNRLVDIINPNRKTIEQLMTLDLPTGVEIEIKT

00100010001000100000000000000000101111111100010011111110110100000100001000000000000000000000001000

>1XMQ_K

KRQVASGRAYIHASYNNTIVTITDPDGNPITWSSGGVIGYKGSRKGTPYAAQLAALDAAKKAMAYGMQSVDVIVRGTGAGREQAIRALQASGLQVKSIVDDTPVPHNGCRPKKKFRKAS

00000000010000011010000000111001010000001110100000000000000000000000000000100000000000000000000000000001111111001100000

>1XMQ_L

PTINQLVRKGREKVRKKSKVPALKGAPFRRGVCTVVRTVTPKKPNSALRKVAKVRLTSGYEVTAYIPGEGHNLQEHSVVLIRGGRVKDLPGVRYHIVRGVYDAAGVKDRKKSRSKYGTKKPKEAA

11011001001110111010000111111000000000000110111010001000100000001001100000000000010000110110100000000000000011111110101100000

>1XMQ_M

RIAGVEIPRNKRVDVALTYIYGIGKARAKEALEKTGINPATRVKDLTEAEVVRLREYVENTWKLEGELRAEVAANIKRLMDIGCYRGLRHRRGLPVRGQRTRTNARTRKGPRKTVAGKKKAPRK

0000000000110000000111111110000000000000010000000000000000000000000000000010000000001100110001101011111101101001111000011011

>1XMQ_N

ARKALIEKAKRTPKFKVRAYTRCVRCGRARSVYRFFGLCRICLRELAHKGQLPGVRKASW

111100100000011101001000000100100100000110011000000000001011

>1XMQ_P

MVKIRLARFGSKHNPHYRIVVTDARRKRDGKYIEKIGYYDPRKTTPDWLKVDVERARYWLSVGAQPTDTARRLLRQAGVFRQE

10101101110111001100001110110010001000001111000000000000000011100011100100100000110

>1XMQ_Q

PKKVLTGVVVSDKMQKTVTVLVERQFPHPLYGKVIKRSKKYLAHDPEEKYKLGDVVEIIESRPISKRKRFRVLRLVESGRMDLVEKYLIRRQNYQSLSKRGGKA

11000000001000101000000110000100100010111000000000000000000101111111101000000000000000000100110011011011

>1XMQ_R

PSRKAKVKATLGEFDLRDYRNVEVLKRFLSETGKILPRRRTGLSGKEQRILAKTIKRARILGLLPFTEKLVRK

0000000000000000000000000000000001100100000001011001100110110000010000000

>1XMQ_S

PRSLKKGVFVDDHLLEKVLELNAKGEKRLIKTWSRRSTIVPEMVGHTIAVYNGKQHVPVYITENMVGHKLGEFAPTRTYR

11111000100000001000000000000000101100000000000000111100000000000000100000011110

>1XMQ_T

RNLSALKRHRQSLKRRLRNKAKKSAIKTLSKKAIQLAQEGKAEEALKIMRKAESLIDKAAKGSTLHKNAAARRKSRLMRKVRQLLEAAGAPLIGGGLSA

001000111111001100110101001100110000000000000000010011001100101000110011011101110100000000000111010

>1XMQ_V

GKGDRRTRRGKIWRGTYGKYRPRK

111011011010110111110010

>1XOK_C

GKPTKRSQNYAALRK

100001100100000

>1XOK_D

GKAGKPTKRSQNYAALRK

000100001110100000

>1YTU_A

EYKIVENGLTYRIGNGASVPISNTGELIKGLRNYGPYEVPSLKYNQIALIHNNQFSSLINQLKSQISSKIDEVWHIHNINISEFIYDSPHFDSIKSQVDNAIDTGVDGILVLPEYNTPLYYKLKSYLINSIPSQFRYDILSNRNLTFYVDNLLVQFVSKLGGKPWILNVDPEKGSDIIIGTGATRIDNVNLFCFAVFKKDGTLWNEISPIVTSSEYLTYLKSTIKKVVYGFKKSNPDWDVEKLTLHVSGKRPKKDGETKILKETVEELKKQEVSRDVKYAILHLNETHPFWVGDPYEGTKVKLSSKRYLLTLLQPYPIKPLSVEIVSDNWTSEEYYHNVHEILDEIYYLSKNWRGFRSRNLPVTVNYPKLVAGIIANVNRYGGYPINPEGNRSLQTNPWFL

00000000000000000000000000000000000000000000000000000000000000000000000000000000000000000000000000000000000000000010000000000000000000010000000000110000001000100000000000000000000000000000000000000000000000000000000000000000000000000000000000000000000000000000000000000000000000000000000000000000000000000000000000000000000000000000000000000000000000010100101000000000000000000000000000000000000000001

>1YVP_A

MDQTQPLNEKQVPNSEGCYVWQVSDMNRLRRFLCFGSEGGTYYIEEKKLGQENAEALLRLIEDGKGCEVVQEIKTFSQEGRAAKQEPTLFALAVCSQCSDIKTKQAAFRAVPEVCRIPTHLFTFIQFKKDLKEGMKCGMWGRALRKAVSDWYNTKDALNLAMAVTKYKQRNGWSHKDLLRLSHIKPANEGLTMVAKYVSKGWKEVQEAYKEKELSPETEKVLKYLEATERVKRTKDELEIIHLIDEYRLVREHLLTIHLKSKEIWKSLLQDMPLTALLRNLGKMTADSVLAPASSEVSSVCERLTNEKLLKKARIHPFHILVALETYKKGHGLRWIPDTSIVEALDNAFYKSFKLVEPTGKRFLLAIDVSASMNQRVLGSILNASVVAAAMCMLVARTEKDSHMVAFSDEMLPCPITVNMLLHEVVEKMSDITMGSTDCALPMLWAQKTNTAADIFIVFTDCETNVEDVHPATALKQYREKMGIPAKLIVCAMTSNGFSIADPDDRGMLDICGFDSGALDVIRNFTLDL

0000000000000000000000000000000000000000000000000000000000000000000000000000000000000000000000000000000110000000000000000000000010010000010101001000000000000000000000000001000010011010000000000000000000000000000000000000000000000000000000000000000000000000000000000000000000000000000000000000000000000000000000000000000000000000000000000000000000000000000000000000000000000000000000000000000000000000000000000000000000000000000000000000000000000000000000000000000000000000000000000000000000000000000000000000000000000000000000000

>1ZE2_A

MKHGILVAYKPKGPTSHDVVDEVRKKLKTRKVGHGGTLDPFACGVLIIGVNQGTRILEFYKDLKKVYWVKMRLGLITETFDITGEVVEERECNVTEEEIREAIFSFVGEYDQVPPAYSAKKYKGERLYKLAREGKIINLPPKRVKIFKIWDVNIEGRDVSFRVEVSPGTYIRSLCMDIGYKLGCGATAVELVRESVGPHTIEESLNVFEAAPEEIENRIIPLEKCLEWLPRVVVHQESTKMILNGSQIHLEMLKEWDGFKKGEVVRVFNEEGRLLALAEAERNSSFRQERVLTLRKVFQT

000000000000001010000000000000110100101100000000000101100100000000000000000000100000000000000000000000000000000000000001101001010001000000000000000000000000000000000000010000000000000000000000000000000000000000000000000000000000000000000000000111000000000000000000000000000000000000000000000000011000

>1ZE2_B

MKHGILVAYKPKGPTSHDVVDEVRKKLKTRKVGHGGTLDPFACGVLIIGVNQGTRILEFYKDLKKVYGEYDQVPPAYSAKKYKGERLYKLAREGKIINLPPKRVKIFKIRVEVSPGTYIRVRESVGPHTIEESLNVFEAAPEEIENRIIPLEKCLEWLPRVVVHQESTKMILNGSQIHLEMLKEWDGFKKGEVVRVFNEEGRLLALAEAERNRKVFQT

00000000000000101000000100000101100010011000000000010110100010000000000000001001100001010001000000000000000000000000010000000000000000000000000000000000000000000000000000001000000000000000000000000000000000000000111000

>1ZJW_A

TNFIRQIIDEDLASGKHTTVHTRFPPEPNGYLHIGHAKSICLNFGIAQDYKGQCNLRFDDTNPVKEDIEYVESIKNDVEWLGFHWSGNVRYSSDYFDQLHAYAIELINKGLAYVDELTPEQIREYRGTLTQPGKNSPYRDRSVEENLALFEKMRAGGFEEGKACLRAKIDMASPFIVMRDPVLYRIKFAEHHQTGNKWCIYPMYDFTHCISDALEGITHSLCTLEFQDNRRLYDWVLDNITIPVHPRQYEFSRLNLEYTVMSKRKLNLLVTDKHVEGWDDPRMPTISGLRRRGYTAASIREFCKRIGVTKQDNTIEMASLESCIREDLNENAPRAMAVIDPVKLVIENYQGEGEMVTMPNHPNKPEMGSRQVPFSGEIWIDRADFREEANKQYKRLVLGKEVRLRNAYVIKAERVEKDAEGNITTIFCTYDADTLGVIHWVSAAHALPVEIRLYDRLFSVPNPGAADDFLSVINPESLVIKQGFAEPSLKDAVAGKAFQFEREGYFCLDSRHSTAEKPVFNRTVGLRDT

0000010000000000000000000010000000000000000000000000000000000100000000000000000000000000000000000000000000000000000010000010010010000000000000000000000000000000100000000000011100000000101000000000000000010000000000000000000000110010000000000000000000000000000000000000000000000000000000000000000000000000010000111101001000000000100001000000000000000000000000000010000000000000000000000000000100000000000011000000000000000000000000000000000000000000000000000000000000000000000000000000000000000000001001000000000000000000000000101

>2A8V_A

MNLTELKNTPVSELITLGENMGLENLARMRKQDIIFAILKQHAKSGEDIFGDGVLEILQDGFGFLRSADSSYLAGPDDIYVSPSQIRRFNLRTGDTISGKIRPPKEGERYFALLKVNE

0000000000000000000000000000000000000000000000000000000000000100000000000000000100000000000000000000000000000100000000

>2BBV_F

ASMWERVKSIIKSSLA

0000000100000000

>2BH2_A

QIITVSVNDLDSFGQGVARHNGKTLFIPGLLPQENAEVTVTEDKKQYARAKVVRRLSDSPERETPRCPHFGVCGGCQQQHASVDLQQRSKSAALARLMKHDVSEVIADVPWGYRRRARLSLNYLPKTQQLQMGFRKAGSSDIVDVKQCPILAPQLEALLPKVRACLGSLQAMRHLGHVELVQATSGTLMILRHTAPLSSADREKLERFSHSEGLDLYLAPDSEILETVSGEMPWYDSNGLRLTFSPRDFIQVNAGVNQKMVARALEWLDVQPEDRVLDLFCGMGNFTLPLATQAASVVGVEGVPALVEKGQQNARLNGLQNVTFYHENLEEDVTKQPWAKNGFDKVLLDPARAGAAGVMQQIIKLEPIRIVYVSCNPATLARDSEALLKAGYTIARLAMLDMFPHTGHLESMVLFSRV

0000000010101010001000010100000000000000000111100000000000000000000000000110000000000000000000010000000000000000010001001100000000000110010000000000000000000000000000000000001110000000000000010000000000000000000000000000000000000000000000000000000000000000000000000000000000000000000000000000000000000000000000000000000000000001000000000000000000000001000000000000000000000001010001000000000000000000000000010000000000
