# Supplementary material for: Predicting RNA-binding sites of proteins using support vector machines and evolutionary information
Source: BMC Bioinformatics. 2008 Dec 12;9(Suppl 12):S6. doi: 10.1186/1471-2105-9-S12-S6 (PMC2638146; doi:10.1186/1471-2105-9-S12-S6)
Supplement: Additional file 4 — Detailed experimental results on the RBP86 data set. [file 1471-2105-9-S12-S6-S4.doc]

# Experiment results of the RBP86

Table A 1. The detail performance of RBP86 with (A) different sliding window size under five-fold cross-validation (w1 = 3.39, w-1 = 1, other parameters: default value) and (B) different sliding window size under three-way data split (w1 = 3.39, w-1 = 1, other parameters: default value).

1. Five-fold cross-validation

| **Window Size** | **Spec.** | **Sens.** | **MCC** | **Acc** |
| --- | --- | --- | --- | --- |
| **3** | 73.38% | 66.16% | 0.35 | 71.74% |
| **5** | 75.60% | 66.94% | 0.38 | 73.63% |
| **7** | 76.81% | 67.62% | 0.40 | 74.72% |
| **9** | 77.30% | 69.33% | 0.41 | 75.49% |
| **11** | 77.92% | 68.96% | 0.42 | 75.88% |
| **13** | 78.06% | 69.00% | 0.42 | 76.00% |
| **15** | 78.71% | 69.33% | 0.43 | 76.58% |
| **17** | 78.77% | 69.48% | 0.43 | 76.65% |
| **19** | 78.87% | 69.40% | 0.43 | 76.71% |
| **21** | 78.75% | 69.46% | 0.43 | 76.64% |
| **23** | 78.93% | 69.94% | 0.44 | 76.88% |
| **25** | 79.22% | 69.75% | 0.44 | 77.06% |
| **27** | 79.24% | 69.88% | 0.44 | 77.11% |
| **29** | 79.38% | 69.66% | 0.44 | 77.17% |
| **31** | 79.26% | 69.70% | 0.44 | 77.08% |
| **33** | 79.36% | 69.77% | 0.44 | 77.18% |
| **35** | 79.41% | 69.86% | 0.44 | 77.24% |
| **37** | 79.77% | 69.55% | 0.45 | 77.45% |
| **39** | 79.50% | 69.81% | 0.44 | 77.30% |
| **41** | 79.66% | 70.05% | 0.45 | 77.47% |

1. Three-way data split

| **Window Size** | **Spec.** | **Sens.** | **MCC** | **Acc** |
| --- | --- | --- | --- | --- |
| **3** | 73.59% | 66.20% | 0.35 | 71.91% |
| **5** | 75.59% | 66.24% | 0.37 | 73.46% |
| **7** | 76.77% | 66.81% | 0.39 | 74.50% |
| **9** | 76.93% | 68.08% | 0.40 | 74.91% |
| **11** | 77.70% | 68.13% | 0.41 | 75.52% |
| **13** | 78.17% | 68.35% | 0.42 | 75.93% |
| **15** | 78.44% | 68.43% | 0.42 | 76.16% |
| **17** | 78.60% | 68.59% | 0.42 | 76.32% |
| **19** | 78.77% | 68.65% | 0.43 | 76.46% |
| **21** | 78.85% | 69.24% | 0.43 | 76.66% |
| **23** | 79.20% | 68.78% | 0.43 | 76.83% |
| **25** | 79.38% | 69.35% | 0.44 | 77.10% |
| **27** | 79.47% | 69.40% | 0.44 | 77.18% |
| **29** | 79.45% | 69.26% | 0.44 | 77.13% |
| **31** | 79.41% | 69.70% | 0.44 | 77.20% |
| **33** | 79.40% | 69.33% | 0.44 | 77.11% |
| **35** | 79.58% | 69.13% | 0.44 | 77.21% |
| **37** | 79.82% | 69.00% | 0.44 | 77.36% |
| **39** | 79.67% | 69.33% | 0.44 | 77.32% |
| **41** | 79.78% | 69.37% | 0.44 | 77.42% |

1. Five-fold cross-validation.


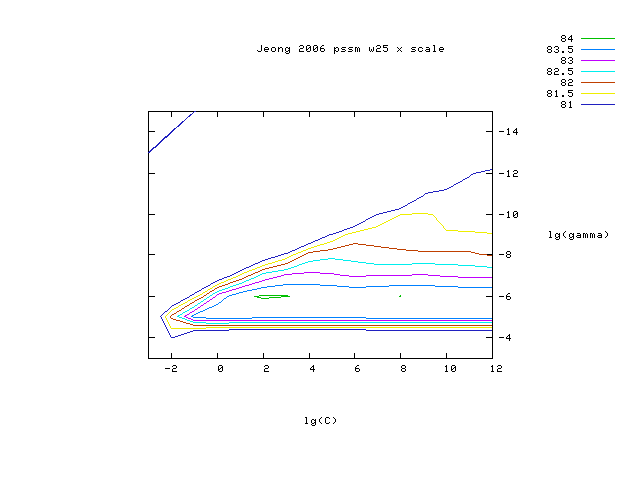


1. Three-way data split.


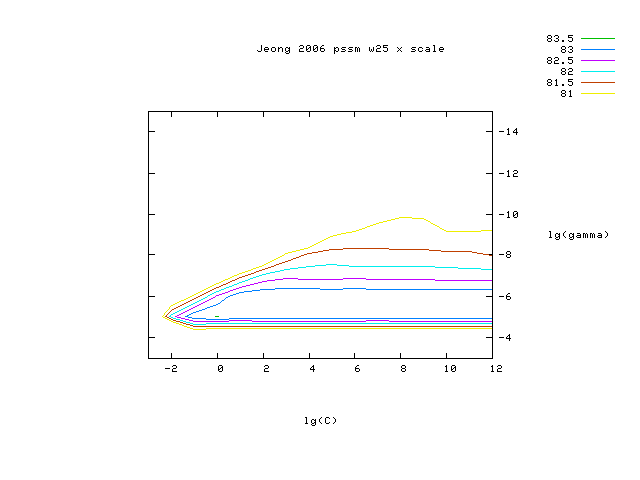


Figure A 1. The performance with different combination of C and γ in the RBP86 data set under (A) five-fold cross-validation and (B) three-way data split.

Table A 2. The detail performance of the RBP86 with (A) different smoothing window size under five-fold cross-validation (w = 25, log C = 2, log γ = -6, w1 = 3.39, w-1 = 1, other parameters: default value) and (B) different smoothing window size under three-way data split (w = 25, log C = 0, log γ = -5, w1 = 3.39, w-1 = 1, other parameters: default value).

1. Five-fold cross-validation.

| **Smoothing Window Size** | **Spec.** | **Sens.** | **MCC** | **Acc** |
| --- | --- | --- | --- | --- |
| **1** | 94.37% | 48.18% | 0.50 | 83.86% |
| **3** | 92.95% | 69.64% | 0.64 | 87.64% |
| **5** | 91.67% | 76.58% | 0.67 | 88.23% |
| **7** | 90.12% | 80.36% | 0.67 | 87.90% |
| **9** | 89.28% | 82.82% | 0.68 | 87.81% |
| **11** | 88.34% | 84.37% | 0.68 | 87.44% |

1. Three-way data split.

| **Smoothing Window Size** | **Spec.** | **Sens.** | **MCC** | **Acc** |
| --- | --- | --- | --- | --- |
| **1** | 96.40% | 38.68% | 0.46 | 83.26% |
| **3** | 92.14% | 66.53% | 0.60 | 86.31% |
| **5** | 90.72% | 76.95% | 0.66 | 87.58% |
| **7** | 89.65% | 80.34% | 0.67 | 87.53% |
| **9** | 88.62% | 82.84% | 0.67 | 87.31% |
| **11** | 88.04% | 83.87% | 0.67 | 87.09% |

Table A 3. The detail performance of the RBP86 with (A) different weight parameter w1 under five-fold cross-validation (w = 25, log C = 2, log γ = -6, ws = 7, w-1 = 1, other parameters: default value) and (B) different weight parameter w1 under three-way data split (w = 25, log C = 0, log γ = -5, ws = 7, w-1 = 1, other parameters: default value).

1. Five-fold cross-validation.

| **W1** | **Spec.** | **Sens.** | **MCC** | **Acc** |
| --- | --- | --- | --- | --- |
| **1** | 96.01% | 62.26% | 0.65 | 88.33% |
| **2** | 92.33% | 75.28% | 0.67 | 88.45% |
| **3** | 90.60% | 79.73% | 0.68 | 88.13% |
| **4** | 89.80% | 81.37% | 0.68 | 87.88% |
| **5** | 89.42% | 81.74% | 0.67 | 87.67% |
| **6** | 89.23% | 82.05% | 0.67 | 87.60% |
| **7** | 89.12% | 82.20% | 0.67 | 87.55% |
| **8** | 89.04% | 82.18% | 0.67 | 87.48% |

1. Three-way data split.

| **W1** | **Spec.** | **Sens.** | **MCC** | **Acc** |
| --- | --- | --- | --- | --- |
| **1** | 96.79% | 56.41% | 0.62 | 87.60% |
| **2** | 92.17% | 73.53% | 0.66 | 87.93% |
| **3** | 90.18% | 78.94% | 0.66 | 87.62% |
| **4** | 89.07% | 81.68% | 0.67 | 87.38% |
| **5** | 88.56% | 82.44% | 0.67 | 87.17% |
| **6** | 88.22% | 82.73% | 0.66 | 86.97% |
| **7** | 88.08% | 82.86% | 0.66 | 86.89% |
| **8** | 88.02% | 82.97% | 0.66 | 86.87% |

Table A 4. The RBP86 data set experiment results with –b option in SVM for (A) smoothed PSSM by five-fold cross-validation, (B) standard PSSM by five-fold cross-validation, (C) smoothed PSSM by three-way data split, and (D) standard PSSM by three-way data split.

1. The experiment result of smoothed PSSM with –b option in SVM by five-fold cross-validation.

| **Threshold** | **Spec.** | **Sens.** | **MCC** | **Threshold** | **Spec.** | **Sens.** | **MCC** |
| --- | --- | --- | --- | --- | --- | --- | --- |
| **0** | 0.00% | 100.00% | 0.00 | **0.51** | 93.83% | 71.21% | 0.67 |
| **0.01** | 13.08% | 99.54% | 0.17 | **0.52** | 93.96% | 70.71% | 0.67 |
| **0.02** | 27.49% | 99.01% | 0.27 | **0.53** | 94.18% | 70.07% | 0.67 |
| **0.03** | 39.48% | 98.16% | 0.34 | **0.54** | 94.43% | 69.29% | 0.67 |
| **0.04** | 48.89% | 97.31% | 0.40 | **0.55** | 94.61% | 68.78% | 0.67 |
| **0.05** | 56.51% | 96.67% | 0.45 | **0.56** | 94.74% | 68.13% | 0.66 |
| **0.06** | 62.70% | 96.06% | 0.49 | **0.57** | 94.90% | 67.21% | 0.66 |
| **0.07** | 67.71% | 95.10% | 0.53 | **0.58** | 95.08% | 66.35% | 0.66 |
| **0.08** | 70.92% | 94.53% | 0.55 | **0.59** | 95.23% | 65.41% | 0.65 |
| **0.09** | 73.43% | 93.76% | 0.57 | **0.6** | 95.35% | 64.89% | 0.65 |
| **0.1** | 75.29% | 93.15% | 0.59 | **0.61** | 95.58% | 64.14% | 0.65 |
| **0.11** | 77.03% | 92.64% | 0.60 | **0.62** | 95.79% | 63.35% | 0.65 |
| **0.12** | 78.46% | 92.05% | 0.61 | **0.63** | 95.94% | 62.70% | 0.65 |
| **0.13** | 79.69% | 91.46% | 0.62 | **0.64** | 96.10% | 61.93% | 0.65 |
| **0.14** | 80.71% | 90.98% | 0.63 | **0.65** | 96.32% | 60.68% | 0.64 |
| **0.15** | 81.67% | 90.56% | 0.64 | **0.66** | 96.52% | 59.63% | 0.64 |
| **0.16** | 82.48% | 90.06% | 0.64 | **0.67** | 96.72% | 58.67% | 0.64 |
| **0.17** | 83.23% | 89.45% | 0.65 | **0.68** | 96.88% | 57.42% | 0.63 |
| **0.18** | 83.76% | 88.88% | 0.65 | **0.69** | 97.06% | 56.44% | 0.63 |
| **0.19** | 84.33% | 88.53% | 0.65 | **0.7** | 97.17% | 55.36% | 0.62 |
| **0.2** | 84.87% | 88.03% | 0.66 | **0.71** | 97.37% | 54.07% | 0.62 |
| **0.21** | 85.42% | 87.52% | 0.66 | **0.72** | 97.53% | 52.74% | 0.61 |
| **0.22** | 85.89% | 87.00% | 0.66 | **0.73** | 97.67% | 51.29% | 0.60 |
| **0.23** | 86.34% | 86.67% | 0.67 | **0.74** | 97.79% | 50.00% | 0.59 |
| **0.24** | 86.73% | 86.25% | 0.67 | **0.75** | 97.96% | 48.71% | 0.59 |
| **0.25** | 87.14% | 85.73% | 0.67 | **0.76** | 98.06% | 46.61% | 0.57 |
| **0.26** | 87.48% | 85.22% | 0.67 | **0.77** | 98.20% | 44.51% | 0.56 |
| **0.27** | 87.86% | 84.63% | 0.67 | **0.78** | 98.37% | 42.78% | 0.55 |
| **0.28** | 88.20% | 84.15% | 0.67 | **0.79** | 98.52% | 41.20% | 0.54 |
| **0.29** | 88.50% | 83.65% | 0.67 | **0.8** | 98.61% | 39.01% | 0.53 |
| **0.3** | 88.76% | 83.17% | 0.67 | **0.81** | 98.75% | 37.41% | 0.52 |
| **0.31** | 89.06% | 82.71% | 0.68 | **0.82** | 98.87% | 35.49% | 0.50 |
| **0.32** | 89.37% | 82.14% | 0.68 | **0.83** | 98.95% | 33.69% | 0.49 |
| **0.33** | 89.54% | 81.87% | 0.68 | **0.84** | 99.03% | 31.94% | 0.48 |
| **0.34** | 89.77% | 81.33% | 0.68 | **0.85** | 99.18% | 29.62% | 0.46 |
| **0.35** | 90.06% | 80.71% | 0.68 | **0.86** | 99.34% | 27.50% | 0.45 |
| **0.36** | 90.36% | 79.95% | 0.68 | **0.87** | 99.44% | 25.15% | 0.43 |
| **0.37** | 90.66% | 79.18% | 0.67 | **0.88** | 99.55% | 23.05% | 0.41 |
| **0.38** | 90.94% | 78.57% | 0.67 | **0.89** | 99.63% | 21.04% | 0.39 |
| **0.39** | 91.20% | 77.87% | 0.67 | **0.9** | 99.71% | 18.76% | 0.37 |
| **0.4** | 91.47% | 77.32% | 0.67 | **0.91** | 99.77% | 16.62% | 0.35 |
| **0.41** | 91.78% | 76.86% | 0.68 | **0.92** | 99.81% | 14.45% | 0.33 |
| **0.42** | 91.99% | 76.29% | 0.67 | **0.93** | 99.86% | 12.52% | 0.31 |
| **0.43** | 92.22% | 75.83% | 0.68 | **0.94** | 99.90% | 10.66% | 0.28 |
| **0.44** | 92.40% | 75.15% | 0.67 | **0.95** | 99.94% | 8.91% | 0.26 |
| **0.45** | 92.67% | 74.54% | 0.67 | **0.96** | 99.95% | 7.25% | 0.23 |
| **0.46** | 92.85% | 73.99% | 0.67 | **0.97** | 99.97% | 5.58% | 0.21 |
| **0.47** | 92.99% | 73.42% | 0.67 | **0.98** | 99.98% | 3.96% | 0.17 |
| **0.48** | 93.21% | 72.77% | 0.67 | **0.99** | 99.99% | 1.88% | 0.12 |
| **0.49** | 93.38% | 72.22% | 0.67 | **1** | 100.00% | 0.00% | 0.00 |
| **0.5** | 93.70% | 71.41% | 0.67 |  |  |  |  |

1. The experiment result of standard PSSM with –b option in SVM by five-fold cross-validation.

| **Threshold** | **Spec.** | **Sens.** | **MCC** | **Threshold** | **Spec.** | **Sens.** | **MCC** |
| --- | --- | --- | --- | --- | --- | --- | --- |
| **0** | 0.00% | 100.00% | 0.00 | **0.51** | 95.84% | 43.91% | 0.49 |
| **0.01** | 0.73% | 100.00% | 0.04 | **0.52** | 96.08% | 43.06% | 0.49 |
| **0.02** | 4.15% | 99.65% | 0.09 | **0.53** | 96.37% | 42.16% | 0.49 |
| **0.03** | 8.98% | 99.08% | 0.13 | **0.54** | 96.54% | 40.94% | 0.48 |
| **0.04** | 14.75% | 98.29% | 0.17 | **0.55** | 96.74% | 40.11% | 0.48 |
| **0.05** | 20.42% | 97.31% | 0.20 | **0.56** | 96.90% | 39.43% | 0.48 |
| **0.06** | 26.66% | 95.93% | 0.23 | **0.57** | 97.07% | 38.59% | 0.48 |
| **0.07** | 33.99% | 94.48% | 0.27 | **0.58** | 97.19% | 37.63% | 0.47 |
| **0.08** | 39.66% | 92.93% | 0.29 | **0.59** | 97.36% | 36.91% | 0.47 |
| **0.09** | 44.46% | 91.51% | 0.31 | **0.6** | 97.48% | 36.21% | 0.47 |
| **0.1** | 49.17% | 90.30% | 0.34 | **0.61** | 97.64% | 35.25% | 0.46 |
| **0.11** | 53.23% | 88.68% | 0.35 | **0.62** | 97.83% | 34.30% | 0.46 |
| **0.12** | 56.49% | 87.22% | 0.37 | **0.63** | 97.94% | 33.60% | 0.46 |
| **0.13** | 59.52% | 86.08% | 0.38 | **0.64** | 98.08% | 32.82% | 0.45 |
| **0.14** | 62.39% | 84.41% | 0.39 | **0.65** | 98.26% | 31.59% | 0.45 |
| **0.15** | 64.93% | 83.10% | 0.40 | **0.66** | 98.34% | 30.91% | 0.44 |
| **0.16** | 67.26% | 81.66% | 0.41 | **0.67** | 98.42% | 30.06% | 0.44 |
| **0.17** | 69.34% | 80.43% | 0.42 | **0.68** | 98.51% | 29.07% | 0.43 |
| **0.18** | 71.17% | 78.59% | 0.43 | **0.69** | 98.58% | 28.04% | 0.42 |
| **0.19** | 73.13% | 77.30% | 0.43 | **0.7** | 98.65% | 27.04% | 0.42 |
| **0.2** | 75.02% | 76.03% | 0.44 | **0.71** | 98.71% | 26.36% | 0.41 |
| **0.21** | 76.55% | 74.58% | 0.45 | **0.72** | 98.79% | 25.48% | 0.41 |
| **0.22** | 77.71% | 73.47% | 0.45 | **0.73** | 98.86% | 24.54% | 0.40 |
| **0.23** | 79.02% | 72.29% | 0.46 | **0.74** | 98.94% | 23.82% | 0.39 |
| **0.24** | 80.35% | 71.28% | 0.47 | **0.75** | 99.05% | 23.14% | 0.39 |
| **0.25** | 81.46% | 70.16% | 0.47 | **0.76** | 99.09% | 22.53% | 0.39 |
| **0.26** | 82.46% | 69.11% | 0.48 | **0.77** | 99.13% | 21.63% | 0.38 |
| **0.27** | 83.42% | 67.78% | 0.48 | **0.78** | 99.19% | 20.86% | 0.37 |
| **0.28** | 84.42% | 66.97% | 0.48 | **0.79** | 99.23% | 20.14% | 0.37 |
| **0.29** | 85.33% | 65.78% | 0.49 | **0.8** | 99.27% | 19.40% | 0.36 |
| **0.3** | 86.04% | 64.65% | 0.49 | **0.81** | 99.32% | 18.43% | 0.35 |
| **0.31** | 86.89% | 63.42% | 0.49 | **0.82** | 99.37% | 17.64% | 0.34 |
| **0.32** | 87.61% | 62.26% | 0.49 | **0.83** | 99.40% | 16.97% | 0.34 |
| **0.33** | 88.05% | 61.62% | 0.49 | **0.84** | 99.48% | 16.00% | 0.33 |
| **0.34** | 88.76% | 60.55% | 0.50 | **0.85** | 99.55% | 15.11% | 0.32 |
| **0.35** | 89.44% | 59.52% | 0.50 | **0.86** | 99.60% | 14.19% | 0.31 |
| **0.36** | 90.03% | 58.38% | 0.50 | **0.87** | 99.64% | 13.05% | 0.30 |
| **0.37** | 90.55% | 57.27% | 0.50 | **0.88** | 99.65% | 12.13% | 0.29 |
| **0.38** | 91.10% | 56.30% | 0.50 | **0.89** | 99.70% | 10.75% | 0.27 |
| **0.39** | 91.66% | 55.34% | 0.50 | **0.9** | 99.74% | 9.52% | 0.26 |
| **0.4** | 92.10% | 54.33% | 0.50 | **0.91** | 99.79% | 8.12% | 0.24 |
| **0.41** | 92.59% | 53.06% | 0.50 | **0.92** | 99.84% | 7.05% | 0.22 |
| **0.42** | 92.92% | 52.21% | 0.50 | **0.93** | 99.86% | 6.22% | 0.21 |
| **0.43** | 93.27% | 51.34% | 0.50 | **0.94** | 99.94% | 5.30% | 0.20 |
| **0.44** | 93.65% | 50.35% | 0.50 | **0.95** | 99.95% | 4.29% | 0.18 |
| **0.45** | 94.11% | 49.43% | 0.50 | **0.96** | 99.97% | 3.46% | 0.16 |
| **0.46** | 94.53% | 48.27% | 0.50 | **0.97** | 99.99% | 2.50% | 0.14 |
| **0.47** | 94.83% | 47.48% | 0.50 | **0.98** | 100.00% | 1.44% | 0.11 |
| **0.48** | 95.08% | 46.56% | 0.50 | **0.99** | 100.00% | 0.46% | 0.06 |
| **0.49** | 95.35% | 45.84% | 0.50 | **1** | 100.00% | 0.00% | 0.00 |
| **0.5** | 95.70% | 44.44% | 0.49 |  |  |  |  |

1. The experiment result of smoothed PSSM with –b option in SVM by three-way data split

| **Threshold** | **Spec.** | **Sens.** | **MCC** | **Threshold** | **Spec.** | **Sens.** | **MCC** |
| --- | --- | --- | --- | --- | --- | --- | --- |
| **0** | 0.00% | 100.00% | 0.00 | **0.51** | 93.44% | 70.88% | 0.66 |
| **0.01** | 6.91% | 99.87% | 0.13 | **0.52** | 93.58% | 70.14% | 0.66 |
| **0.02** | 19.24% | 99.39% | 0.22 | **0.53** | 93.72% | 69.59% | 0.66 |
| **0.03** | 32.53% | 98.69% | 0.30 | **0.54** | 93.88% | 68.87% | 0.65 |
| **0.04** | 45.75% | 97.75% | 0.38 | **0.55** | 94.07% | 68.30% | 0.65 |
| **0.05** | 55.62% | 96.80% | 0.44 | **0.56** | 94.28% | 67.40% | 0.65 |
| **0.06** | 63.25% | 96.19% | 0.50 | **0.57** | 94.45% | 66.86% | 0.65 |
| **0.07** | 69.07% | 95.29% | 0.54 | **0.58** | 94.64% | 66.07% | 0.65 |
| **0.08** | 72.24% | 94.61% | 0.57 | **0.59** | 94.81% | 65.46% | 0.65 |
| **0.09** | 74.63% | 93.98% | 0.58 | **0.6** | 94.99% | 64.62% | 0.64 |
| **0.1** | 76.52% | 93.30% | 0.60 | **0.61** | 95.17% | 63.97% | 0.64 |
| **0.11** | 77.96% | 92.51% | 0.61 | **0.62** | 95.39% | 63.09% | 0.64 |
| **0.12** | 79.00% | 92.05% | 0.62 | **0.63** | 95.60% | 62.19% | 0.64 |
| **0.13** | 79.93% | 91.40% | 0.62 | **0.64** | 95.77% | 61.49% | 0.64 |
| **0.14** | 80.77% | 90.67% | 0.63 | **0.65** | 95.99% | 60.49% | 0.63 |
| **0.15** | 81.58% | 89.91% | 0.63 | **0.66** | 96.19% | 59.39% | 0.63 |
| **0.16** | 82.31% | 89.14% | 0.63 | **0.67** | 96.35% | 58.63% | 0.63 |
| **0.17** | 83.00% | 88.64% | 0.64 | **0.68** | 96.52% | 57.33% | 0.62 |
| **0.18** | 83.62% | 88.11% | 0.64 | **0.69** | 96.69% | 56.35% | 0.62 |
| **0.19** | 84.15% | 87.57% | 0.64 | **0.7** | 96.90% | 54.97% | 0.61 |
| **0.2** | 84.65% | 86.82% | 0.64 | **0.71** | 97.08% | 53.48% | 0.60 |
| **0.21** | 85.10% | 86.32% | 0.65 | **0.72** | 97.26% | 52.08% | 0.60 |
| **0.22** | 85.56% | 85.95% | 0.65 | **0.73** | 97.44% | 50.88% | 0.59 |
| **0.23** | 85.92% | 85.35% | 0.65 | **0.74** | 97.65% | 49.17% | 0.58 |
| **0.24** | 86.36% | 84.92% | 0.65 | **0.75** | 97.83% | 47.59% | 0.58 |
| **0.25** | 86.80% | 84.46% | 0.66 | **0.76** | 98.02% | 45.86% | 0.57 |
| **0.26** | 87.18% | 84.06% | 0.66 | **0.77** | 98.18% | 43.72% | 0.55 |
| **0.27** | 87.45% | 83.54% | 0.66 | **0.78** | 98.32% | 41.81% | 0.54 |
| **0.28** | 87.81% | 83.25% | 0.66 | **0.79** | 98.49% | 39.62% | 0.53 |
| **0.29** | 88.09% | 82.82% | 0.66 | **0.8** | 98.65% | 37.24% | 0.51 |
| **0.3** | 88.36% | 82.51% | 0.66 | **0.81** | 98.79% | 35.09% | 0.50 |
| **0.31** | 88.69% | 82.07% | 0.67 | **0.82** | 98.96% | 32.47% | 0.48 |
| **0.32** | 88.92% | 81.63% | 0.67 | **0.83** | 99.06% | 29.95% | 0.46 |
| **0.33** | 89.14% | 81.41% | 0.67 | **0.84** | 99.19% | 26.99% | 0.44 |
| **0.34** | 89.44% | 80.93% | 0.67 | **0.85** | 99.37% | 23.86% | 0.41 |
| **0.35** | 89.75% | 80.36% | 0.67 | **0.86** | 99.46% | 20.84% | 0.38 |
| **0.36** | 90.01% | 79.64% | 0.67 | **0.87** | 99.60% | 18.19% | 0.36 |
| **0.37** | 90.29% | 79.36% | 0.67 | **0.88** | 99.70% | 15.63% | 0.34 |
| **0.38** | 90.58% | 78.72% | 0.67 | **0.89** | 99.76% | 13.20% | 0.31 |
| **0.39** | 90.80% | 78.04% | 0.67 | **0.9** | 99.85% | 11.12% | 0.29 |
| **0.4** | 91.05% | 77.47% | 0.67 | **0.91** | 99.88% | 9.33% | 0.26 |
| **0.41** | 91.30% | 77.06% | 0.67 | **0.92** | 99.92% | 7.53% | 0.24 |
| **0.42** | 91.58% | 76.38% | 0.67 | **0.93** | 99.95% | 6.35% | 0.22 |
| **0.43** | 91.73% | 75.79% | 0.67 | **0.94** | 99.96% | 5.34% | 0.20 |
| **0.44** | 91.92% | 75.39% | 0.67 | **0.95** | 99.98% | 4.23% | 0.18 |
| **0.45** | 92.10% | 74.80% | 0.67 | **0.96** | 99.99% | 3.26% | 0.16 |
| **0.46** | 92.38% | 74.10% | 0.66 | **0.97** | 99.99% | 2.21% | 0.13 |
| **0.47** | 92.60% | 73.53% | 0.66 | **0.98** | 99.99% | 1.27% | 0.10 |
| **0.48** | 92.76% | 72.70% | 0.66 | **0.99** | 100.00% | 0.42% | 0.06 |
| **0.49** | 92.95% | 72.09% | 0.66 | **1** | 100.00% | 0.00% | 0.00 |
| **0.5** | 93.30% | 71.32% | 0.66 |  |  |  |  |

1. The experiment result of standard PSSM with –b option in SVM by three-way data split

| **Threshold** | **Spec.** | **Sens.** | **MCC** | **Threshold** | **Spec.** | **Sens.** | **MCC** |
| --- | --- | --- | --- | --- | --- | --- | --- |
| **0** | 0.00% | 100.00% | 0.00 | **0.51** | 95.58% | 42.27% | 0.47 |
| **0.01** | 1.92% | 99.87% | 0.06 | **0.52** | 95.84% | 41.53% | 0.47 |
| **0.02** | 5.82% | 99.50% | 0.11 | **0.53** | 96.05% | 40.89% | 0.47 |
| **0.03** | 10.48% | 99.01% | 0.14 | **0.54** | 96.21% | 40.15% | 0.47 |
| **0.04** | 15.75% | 98.29% | 0.18 | **0.55** | 96.42% | 39.14% | 0.46 |
| **0.05** | 21.92% | 97.09% | 0.21 | **0.56** | 96.59% | 38.46% | 0.46 |
| **0.06** | 28.18% | 95.91% | 0.24 | **0.57** | 96.74% | 37.48% | 0.46 |
| **0.07** | 34.69% | 94.81% | 0.28 | **0.58** | 96.90% | 36.67% | 0.45 |
| **0.08** | 39.32% | 93.41% | 0.29 | **0.59** | 97.09% | 36.01% | 0.45 |
| **0.09** | 43.60% | 92.40% | 0.32 | **0.6** | 97.28% | 35.49% | 0.45 |
| **0.1** | 47.60% | 91.24% | 0.33 | **0.61** | 97.43% | 34.92% | 0.45 |
| **0.11** | 51.42% | 89.75% | 0.35 | **0.62** | 97.56% | 34.24% | 0.45 |
| **0.12** | 54.90% | 88.35% | 0.36 | **0.63** | 97.66% | 33.49% | 0.45 |
| **0.13** | 58.21% | 86.87% | 0.38 | **0.64** | 97.74% | 32.92% | 0.44 |
| **0.14** | 61.21% | 85.31% | 0.39 | **0.65** | 97.86% | 31.79% | 0.44 |
| **0.15** | 63.92% | 84.04% | 0.40 | **0.66** | 97.93% | 31.20% | 0.43 |
| **0.16** | 66.37% | 82.53% | 0.41 | **0.67** | 98.02% | 30.67% | 0.43 |
| **0.17** | 68.89% | 81.22% | 0.42 | **0.68** | 98.10% | 29.71% | 0.42 |
| **0.18** | 70.99% | 79.84% | 0.43 | **0.69** | 98.17% | 28.98% | 0.42 |
| **0.19** | 73.11% | 78.31% | 0.44 | **0.7** | 98.30% | 28.31% | 0.42 |
| **0.2** | 74.95% | 76.66% | 0.45 | **0.71** | 98.44% | 27.36% | 0.41 |
| **0.21** | 76.51% | 74.98% | 0.45 | **0.72** | 98.46% | 26.69% | 0.41 |
| **0.22** | 78.14% | 73.66% | 0.46 | **0.73** | 98.55% | 26.03% | 0.40 |
| **0.23** | 79.54% | 72.37% | 0.47 | **0.74** | 98.65% | 25.44% | 0.40 |
| **0.24** | 80.78% | 71.06% | 0.47 | **0.75** | 98.74% | 24.72% | 0.40 |
| **0.25** | 82.00% | 69.68% | 0.47 | **0.76** | 98.82% | 24.10% | 0.39 |
| **0.26** | 83.18% | 68.83% | 0.48 | **0.77** | 98.90% | 23.38% | 0.39 |
| **0.27** | 84.20% | 67.38% | 0.48 | **0.78** | 98.94% | 22.57% | 0.38 |
| **0.28** | 85.22% | 66.22% | 0.49 | **0.79** | 99.01% | 21.89% | 0.38 |
| **0.29** | 86.13% | 64.75% | 0.49 | **0.8** | 99.06% | 21.23% | 0.37 |
| **0.3** | 86.92% | 63.46% | 0.49 | **0.81** | 99.12% | 20.56% | 0.37 |
| **0.31** | 87.67% | 62.30% | 0.49 | **0.82** | 99.17% | 19.81% | 0.36 |
| **0.32** | 88.23% | 61.01% | 0.49 | **0.83** | 99.21% | 19.18% | 0.35 |
| **0.33** | 88.62% | 60.29% | 0.49 | **0.84** | 99.24% | 18.45% | 0.35 |
| **0.34** | 89.26% | 58.80% | 0.49 | **0.85** | 99.27% | 17.86% | 0.34 |
| **0.35** | 89.85% | 57.51% | 0.49 | **0.86** | 99.34% | 17.14% | 0.34 |
| **0.36** | 90.43% | 56.48% | 0.49 | **0.87** | 99.36% | 16.42% | 0.33 |
| **0.37** | 90.97% | 55.54% | 0.49 | **0.88** | 99.41% | 15.59% | 0.32 |
| **0.38** | 91.52% | 54.38% | 0.49 | **0.89** | 99.45% | 14.71% | 0.31 |
| **0.39** | 92.07% | 53.74% | 0.50 | **0.9** | 99.52% | 14.05% | 0.31 |
| **0.4** | 92.47% | 52.50% | 0.49 | **0.91** | 99.56% | 13.31% | 0.30 |
| **0.41** | 92.79% | 51.44% | 0.49 | **0.92** | 99.61% | 12.52% | 0.29 |
| **0.42** | 93.12% | 50.46% | 0.49 | **0.93** | 99.67% | 11.49% | 0.28 |
| **0.43** | 93.46% | 49.47% | 0.49 | **0.94** | 99.69% | 10.25% | 0.26 |
| **0.44** | 93.81% | 48.42% | 0.49 | **0.95** | 99.76% | 8.65% | 0.24 |
| **0.45** | 94.09% | 47.22% | 0.48 | **0.96** | 99.84% | 6.81% | 0.22 |
| **0.46** | 94.39% | 46.50% | 0.48 | **0.97** | 99.93% | 4.84% | 0.19 |
| **0.47** | 94.64% | 45.73% | 0.48 | **0.98** | 99.98% | 3.02% | 0.15 |
| **0.48** | 94.92% | 44.86% | 0.48 | **0.99** | 99.99% | 1.34% | 0.10 |
| **0.49** | 95.19% | 43.94% | 0.48 | **1** | 100.00% | 0.00% | 0.00 |
| **0.5** | 95.50% | 42.54% | 0.47 |  |  |  |  |
