# Supplementary material for: Predicting RNA-binding sites of proteins using support vector machines and evolutionary information
Source: BMC Bioinformatics. 2008 Dec 12;9(Suppl 12):S6. doi: 10.1186/1471-2105-9-S12-S6 (PMC2638146; doi:10.1186/1471-2105-9-S12-S6)
Supplement: Additional file 5 — Detailed experimental results on the RBP109 data set. [file 1471-2105-9-S12-S6-S5.doc]

# Experiment results of the RBP109

Table B 1. The detail performance of the RBP109 with different sliding window size under (A) five-fold cross-validation (w1 = 6.01, w-1 = 1, other parameters: default value) and (B) three-way data split (w1 = 6.01, w-1 = 1, other parameters: default value).

1. Five-fold cross validation.

| **Window Size** | **Spec.** | **Sens.** | **MCC** | **Acc** |
| --- | --- | --- | --- | --- |
| **3** | 74.99% | 67.89% | 0.32 | 73.98% |
| **5** | 76.93% | 68.22% | 0.35 | 75.69% |
| **7** | 77.72% | 68.89% | 0.36 | 76.46% |
| **9** | 78.14% | 69.73% | 0.37 | 76.94% |
| **11** | 78.77% | 69.98% | 0.38 | 77.52% |
| **13** | 79.14% | 69.42% | 0.38 | 77.75% |
| **15** | 79.62% | 70.09% | 0.39 | 78.27% |
| **17** | 79.67% | 70.15% | 0.39 | 78.31% |
| **19** | 79.73% | 69.79% | 0.39 | 78.31% |
| **21** | 79.86% | 69.95% | 0.39 | 78.44% |
| **23** | 80.21% | 69.65% | 0.39 | 78.71% |
| **25** | 80.24% | 69.51% | 0.39 | 78.71% |
| **27** | 80.08% | 69.42% | 0.39 | 78.56% |
| **29** | 80.18% | 69.20% | 0.39 | 78.61% |
| **31** | 80.53% | 69.17% | 0.39 | 78.91% |
| **33** | 80.67% | 68.92% | 0.39 | 79.00% |
| **35** | 80.53% | 69.45% | 0.40 | 78.95% |
| **37** | 80.42% | 69.56% | 0.40 | 78.87% |
| **39** | 80.47% | 68.75% | 0.39 | 78.80% |
| **41** | 80.45% | 68.84% | 0.39 | 78.79% |

1. Three-way data split.

| **Window Size** | **Spec.** | **Sens.** | **MCC** | **Acc** |
| --- | --- | --- | --- | --- |
| **3** | 75.22% | 67.89% | 0.33 | 74.17% |
| **5** | 76.72% | 68.44% | 0.35 | 75.54% |
| **7** | 77.82% | 68.53% | 0.36 | 76.50% |
| **9** | 78.24% | 69.14% | 0.37 | 76.94% |
| **11** | 78.73% | 69.31% | 0.37 | 77.38% |
| **13** | 79.16% | 69.39% | 0.38 | 77.76% |
| **15** | 79.38% | 69.39% | 0.38 | 77.95% |
| **17** | 79.55% | 69.09% | 0.38 | 78.05% |
| **19** | 79.54% | 69.28% | 0.38 | 78.07% |
| **21** | 79.67% | 68.75% | 0.38 | 78.11% |
| **23** | 80.09% | 68.75% | 0.39 | 78.47% |
| **25** | 80.05% | 69.06% | 0.39 | 78.48% |
| **27** | 79.87% | 68.81% | 0.38 | 78.29% |
| **29** | 80.09% | 68.33% | 0.38 | 78.42% |
| **31** | 80.12% | 68.50% | 0.38 | 78.46% |
| **33** | 80.26% | 68.36% | 0.38 | 78.56% |
| **35** | 80.13% | 68.70% | 0.39 | 78.50% |
| **37** | 80.25% | 69.48% | 0.39 | 78.71% |
| **39** | 80.34% | 68.75% | 0.39 | 78.69% |
| **41** | 80.43% | 68.47% | 0.39 | 78.72% |

1. Five-fold cross-validation.


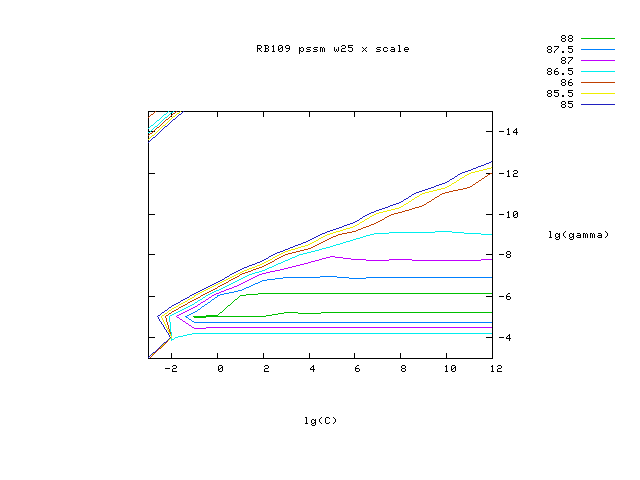


1. Three-way data split.


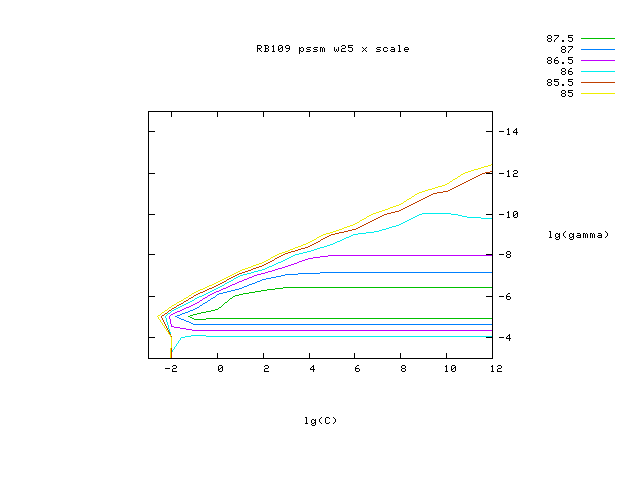


Figure B 1. The performance with different combination of C and γ in the RBP109 data set under (A) five-fold cross-validation and (B) three-way data split.

Table B 2. The detail performance of the RBP109 with (A) different smoothing window size under five-fold cross-validation (w = 25, log C = 2, log γ = -6, w1 = 6.01, w-1 = 1, other parameters: default value) and (B) different smoothing window size under three-way data split (w = 25, log C = 3, log γ = -6, w1 = 6.01, w-1 = 1, other parameters: default value).

1. Five-fold cross-validation.

| **Smoothing Window Size** | **Spec.** | **Sens.** | **MCC** | **Acc** |
| --- | --- | --- | --- | --- |
| **1** | 97.32% | 32.62% | 0.41 | 88.09% |
| **3** | 94.71% | 54.82% | 0.53 | 89.02% |
| **5** | 93.31% | 65.04% | 0.57 | 89.28% |
| **7** | 91.94% | 70.37% | 0.58 | 88.86% |
| **9** | 90.62% | 73.67% | 0.58 | 88.20% |
| **11** | 88.99% | 75.96% | 0.56 | 87.13% |

1. Three-way data split.

| **Smoothing Window Size** | **Spec.** | **Sens.** | **MCC** | **Acc** |
| --- | --- | --- | --- | --- |
| **1** | 97.30% | 30.80% | 0.39 | 87.81% |
| **3** | 95.53% | 46.47% | 0.48 | 88.53% |
| **5** | 94.47% | 57.27% | 0.54 | 89.16% |
| **7** | 93.56% | 62.47% | 0.56 | 89.13% |
| **9** | 92.66% | 65.88% | 0.56 | 88.84% |
| **11** | 91.53% | 68.28% | 0.56 | 88.21% |

Table B 3. The detail performance of the RBP109 with (A) different weight parameter w1 under five-fold cross-validation (w = 25, log C = 2, log γ = -6, ws = 7, w-1 = 1, other parameters: default value) and (B) different weight parameter w1 under three-way data split (w = 25, log C = 3, log γ = -6, ws = 7, w-1 = 1, other parameters: default value).

1. Five-fold cross-validation.

| **W1** | **Spec.** | **Sens.** | **MCC** | **Acc** |
| --- | --- | --- | --- | --- |
| **1** | 97.63% | 41.58% | 0.51 | 89.63% |
| **2** | 94.96% | 58.84% | 0.56 | 89.81% |
| **3** | 93.45% | 66.07% | 0.58 | 89.54% |
| **4** | 92.57% | 68.72% | 0.58 | 89.17% |
| **5** | 92.19% | 69.84% | 0.58 | 89.00% |
| **6** | 91.94% | 70.37% | 0.58 | 88.86% |
| **7** | 91.71% | 70.76% | 0.58 | 88.72% |
| **8** | 91.59% | 70.96% | 0.58 | 88.64% |

1. Three-way data split.

| **W1** | **Spec.** | **Sens.** | **MCC** | **Acc** |
| --- | --- | --- | --- | --- |
| **1** | 96.46% | 48.53% | 0.53 | 89.62% |
| **2** | 94.66% | 59.09% | 0.56 | 89.59% |
| **3** | 93.98% | 61.55% | 0.56 | 89.36% |
| **4** | 93.73% | 62.22% | 0.56 | 89.24% |
| **5** | 93.63% | 62.36% | 0.56 | 89.17% |
| **6** | 93.56% | 62.47% | 0.56 | 89.13% |
| **7** | 93.57% | 62.58% | 0.56 | 89.15% |
| **8** | 93.57% | 62.58% | 0.56 | 89.15% |

Table B 4. The RBP109 data set experiment results with –b option in SVM for (A) smoothed PSSM by five-fold cross-validation, (B) standard PSSM by five-fold cross-validation, (C) smoothed PSSM by three-way data split, and (D) standard PSSM by three-way data split.

1. The experiment result of smoothed PSSM with –b option in SVM by five-fold cross-validation.

| **Threshold** | **Spec.** | **Sens.** | **MCC** | **Threshold** | **Spec.** | **Sens.** | **MCC** |
| --- | --- | --- | --- | --- | --- | --- | --- |
| **0** | 0.00% | 100.00% | 0.00 | **0.51** | 96.58% | 51.05% | 0.55 |
| **0.01** | 13.58% | 99.16% | 0.14 | **0.52** | 96.70% | 50.43% | 0.55 |
| **0.02** | 29.55% | 97.88% | 0.22 | **0.53** | 96.79% | 49.34% | 0.54 |
| **0.03** | 42.20% | 96.51% | 0.28 | **0.54** | 96.91% | 48.59% | 0.54 |
| **0.04** | 52.11% | 95.36% | 0.33 | **0.55** | 97.07% | 47.39% | 0.54 |
| **0.05** | 59.37% | 93.61% | 0.37 | **0.56** | 97.20% | 46.66% | 0.53 |
| **0.06** | 65.68% | 92.24% | 0.41 | **0.57** | 97.36% | 45.71% | 0.53 |
| **0.07** | 70.75% | 90.65% | 0.44 | **0.58** | 97.47% | 44.85% | 0.53 |
| **0.08** | 74.08% | 89.39% | 0.47 | **0.59** | 97.57% | 43.95% | 0.52 |
| **0.09** | 76.63% | 87.82% | 0.48 | **0.6** | 97.68% | 43.00% | 0.52 |
| **0.1** | 78.88% | 86.65% | 0.50 | **0.61** | 97.75% | 41.80% | 0.51 |
| **0.11** | 80.84% | 85.42% | 0.51 | **0.62** | 97.82% | 40.83% | 0.51 |
| **0.12** | 82.20% | 84.25% | 0.52 | **0.63** | 97.91% | 39.74% | 0.50 |
| **0.13** | 83.51% | 83.30% | 0.53 | **0.64** | 98.01% | 38.87% | 0.50 |
| **0.14** | 84.55% | 82.27% | 0.54 | **0.65** | 98.14% | 37.11% | 0.49 |
| **0.15** | 85.63% | 81.35% | 0.55 | **0.66** | 98.26% | 36.41% | 0.49 |
| **0.16** | 86.47% | 80.68% | 0.56 | **0.67** | 98.34% | 35.27% | 0.48 |
| **0.17** | 87.14% | 79.73% | 0.56 | **0.68** | 98.41% | 34.07% | 0.47 |
| **0.18** | 87.73% | 78.75% | 0.56 | **0.69** | 98.53% | 32.95% | 0.47 |
| **0.19** | 88.33% | 77.72% | 0.57 | **0.7** | 98.66% | 31.36% | 0.46 |
| **0.2** | 88.79% | 76.63% | 0.57 | **0.71** | 98.74% | 30.35% | 0.45 |
| **0.21** | 89.31% | 75.82% | 0.57 | **0.72** | 98.83% | 28.79% | 0.44 |
| **0.22** | 89.82% | 75.03% | 0.57 | **0.73** | 98.95% | 27.67% | 0.43 |
| **0.23** | 90.31% | 74.00% | 0.57 | **0.74** | 98.99% | 26.42% | 0.42 |
| **0.24** | 90.76% | 73.05% | 0.58 | **0.75** | 99.08% | 24.91% | 0.41 |
| **0.25** | 91.14% | 72.30% | 0.58 | **0.76** | 99.15% | 23.01% | 0.39 |
| **0.26** | 91.52% | 71.49% | 0.58 | **0.77** | 99.20% | 21.50% | 0.38 |
| **0.27** | 91.86% | 70.57% | 0.58 | **0.78** | 99.25% | 19.99% | 0.37 |
| **0.28** | 92.14% | 69.84% | 0.58 | **0.79** | 99.29% | 18.82% | 0.36 |
| **0.29** | 92.43% | 69.09% | 0.58 | **0.8** | 99.35% | 17.56% | 0.34 |
| **0.3** | 92.69% | 68.39% | 0.58 | **0.81** | 99.41% | 16.03% | 0.33 |
| **0.31** | 92.93% | 67.75% | 0.58 | **0.82** | 99.47% | 14.94% | 0.32 |
| **0.32** | 93.23% | 67.02% | 0.58 | **0.83** | 99.52% | 13.35% | 0.30 |
| **0.33** | 93.36% | 66.43% | 0.58 | **0.84** | 99.58% | 12.20% | 0.29 |
| **0.34** | 93.67% | 65.43% | 0.58 | **0.85** | 99.64% | 10.89% | 0.27 |
| **0.35** | 93.88% | 64.62% | 0.58 | **0.86** | 99.69% | 9.86% | 0.26 |
| **0.36** | 94.08% | 63.75% | 0.58 | **0.87** | 99.74% | 8.99% | 0.25 |
| **0.37** | 94.34% | 63.03% | 0.58 | **0.88** | 99.80% | 7.82% | 0.24 |
| **0.38** | 94.56% | 62.27% | 0.58 | **0.89** | 99.83% | 6.98% | 0.22 |
| **0.39** | 94.75% | 61.63% | 0.58 | **0.9** | 99.85% | 5.81% | 0.20 |
| **0.4** | 94.94% | 60.96% | 0.58 | **0.91** | 99.87% | 4.86% | 0.19 |
| **0.41** | 95.13% | 60.09% | 0.58 | **0.92** | 99.90% | 4.08% | 0.17 |
| **0.42** | 95.31% | 59.23% | 0.58 | **0.93** | 99.91% | 3.55% | 0.16 |
| **0.43** | 95.50% | 58.59% | 0.58 | **0.94** | 99.94% | 2.60% | 0.14 |
| **0.44** | 95.65% | 57.61% | 0.57 | **0.95** | 99.95% | 1.84% | 0.11 |
| **0.45** | 95.82% | 56.44% | 0.57 | **0.96** | 99.98% | 1.37% | 0.10 |
| **0.46** | 95.96% | 55.52% | 0.57 | **0.97** | 99.99% | 0.84% | 0.08 |
| **0.47** | 96.08% | 54.59% | 0.56 | **0.98** | 100.00% | 0.34% | 0.05 |
| **0.48** | 96.20% | 53.62% | 0.56 | **0.99** | 100.00% | 0.11% | 0.03 |
| **0.49** | 96.34% | 52.83% | 0.56 | **1** | 100.00% | 0.00% | 0.00 |
| **0.5** | 96.52% | 51.49% | 0.55 |  |  |  |  |

1. The experiment result of standard PSSM with –b option in SVM by five-fold cross-validation.

| **Threshold** | **Spec.** | **Sens.** | **MCC** | **Threshold** | **Spec.** | **Sens.** | **MCC** |
| --- | --- | --- | --- | --- | --- | --- | --- |
| **0** | 0.00% | 100.00% | 0.00 | **0.51** | 98.00% | 28.20% | 0.39 |
| **0.01** | 2.66% | 99.80% | 0.06 | **0.52** | 98.08% | 27.42% | 0.39 |
| **0.02** | 10.49% | 98.63% | 0.11 | **0.53** | 98.20% | 26.84% | 0.39 |
| **0.03** | 19.59% | 96.65% | 0.15 | **0.54** | 98.31% | 26.19% | 0.39 |
| **0.04** | 28.65% | 94.69% | 0.19 | **0.55** | 98.41% | 25.55% | 0.38 |
| **0.05** | 36.92% | 92.52% | 0.22 | **0.56** | 98.49% | 24.57% | 0.38 |
| **0.06** | 44.75% | 90.51% | 0.25 | **0.57** | 98.56% | 23.93% | 0.37 |
| **0.07** | 52.95% | 87.88% | 0.29 | **0.58** | 98.61% | 23.37% | 0.37 |
| **0.08** | 58.54% | 85.70% | 0.31 | **0.59** | 98.69% | 22.95% | 0.37 |
| **0.09** | 62.91% | 83.55% | 0.33 | **0.6** | 98.74% | 22.54% | 0.37 |
| **0.1** | 66.68% | 81.32% | 0.34 | **0.61** | 98.84% | 22.06% | 0.37 |
| **0.11** | 69.92% | 79.14% | 0.36 | **0.62** | 98.89% | 21.28% | 0.36 |
| **0.12** | 72.72% | 76.60% | 0.36 | **0.63** | 98.94% | 20.75% | 0.36 |
| **0.13** | 75.16% | 74.70% | 0.37 | **0.64** | 98.99% | 20.08% | 0.35 |
| **0.14** | 77.37% | 72.69% | 0.38 | **0.65** | 99.10% | 19.27% | 0.35 |
| **0.15** | 79.16% | 71.32% | 0.39 | **0.66** | 99.18% | 18.68% | 0.35 |
| **0.16** | 80.96% | 69.03% | 0.40 | **0.67** | 99.22% | 17.87% | 0.34 |
| **0.17** | 82.41% | 67.61% | 0.41 | **0.68** | 99.26% | 17.20% | 0.33 |
| **0.18** | 83.96% | 66.13% | 0.42 | **0.69** | 99.32% | 16.56% | 0.33 |
| **0.19** | 85.16% | 64.28% | 0.42 | **0.7** | 99.35% | 15.97% | 0.32 |
| **0.2** | 86.37% | 62.83% | 0.42 | **0.71** | 99.41% | 15.44% | 0.32 |
| **0.21** | 87.43% | 61.13% | 0.43 | **0.72** | 99.46% | 15.02% | 0.32 |
| **0.22** | 88.26% | 59.45% | 0.43 | **0.73** | 99.50% | 14.33% | 0.31 |
| **0.23** | 89.10% | 57.69% | 0.43 | **0.74** | 99.54% | 13.80% | 0.31 |
| **0.24** | 89.89% | 56.46% | 0.43 | **0.75** | 99.55% | 13.07% | 0.30 |
| **0.25** | 90.57% | 55.07% | 0.44 | **0.76** | 99.57% | 12.59% | 0.29 |
| **0.26** | 91.24% | 53.70% | 0.44 | **0.77** | 99.60% | 12.15% | 0.29 |
| **0.27** | 91.88% | 52.33% | 0.44 | **0.78** | 99.63% | 11.51% | 0.28 |
| **0.28** | 92.42% | 50.99% | 0.44 | **0.79** | 99.67% | 11.09% | 0.28 |
| **0.29** | 92.90% | 50.07% | 0.44 | **0.8** | 99.69% | 10.58% | 0.27 |
| **0.3** | 93.38% | 48.90% | 0.44 | **0.81** | 99.73% | 10.16% | 0.27 |
| **0.31** | 93.74% | 48.12% | 0.45 | **0.82** | 99.74% | 9.69% | 0.26 |
| **0.32** | 94.11% | 46.89% | 0.45 | **0.83** | 99.75% | 9.16% | 0.25 |
| **0.33** | 94.37% | 45.96% | 0.44 | **0.84** | 99.78% | 8.63% | 0.25 |
| **0.34** | 94.74% | 44.76% | 0.44 | **0.85** | 99.81% | 7.90% | 0.24 |
| **0.35** | 95.03% | 43.42% | 0.44 | **0.86** | 99.86% | 7.34% | 0.23 |
| **0.36** | 95.29% | 42.11% | 0.44 | **0.87** | 99.88% | 6.65% | 0.22 |
| **0.37** | 95.51% | 40.80% | 0.43 | **0.88** | 99.90% | 6.03% | 0.21 |
| **0.38** | 95.76% | 39.77% | 0.43 | **0.89** | 99.92% | 5.17% | 0.20 |
| **0.39** | 96.04% | 38.73% | 0.43 | **0.9** | 99.93% | 4.66% | 0.19 |
| **0.4** | 96.29% | 37.70% | 0.42 | **0.91** | 99.94% | 4.19% | 0.18 |
| **0.41** | 96.52% | 36.41% | 0.42 | **0.92** | 99.96% | 2.68% | 0.14 |
| **0.42** | 96.71% | 35.41% | 0.42 | **0.93** | 99.98% | 2.01% | 0.13 |
| **0.43** | 96.91% | 34.54% | 0.42 | **0.94** | 99.99% | 1.51% | 0.11 |
| **0.44** | 97.09% | 33.73% | 0.41 | **0.95** | 99.99% | 1.23% | 0.10 |
| **0.45** | 97.25% | 33.04% | 0.41 | **0.96** | 99.99% | 0.98% | 0.09 |
| **0.46** | 97.35% | 32.20% | 0.41 | **0.97** | 100.00% | 0.70% | 0.08 |
| **0.47** | 97.48% | 31.50% | 0.41 | **0.98** | 100.00% | 0.31% | 0.05 |
| **0.48** | 97.62% | 30.58% | 0.40 | **0.99** | 100.00% | 0.03% | 0.02 |
| **0.49** | 97.75% | 29.80% | 0.40 | **1** | 100.00% | 0.00% | 0.00 |
| **0.5** | 97.95% | 28.71% | 0.40 |  |  |  |  |

1. The experiment result of smoothed PSSM with –b option in SVM by three-way data split.

| **Threshold** | **Spec.** | **Sens.** | **MCC** | **Threshold** | **Spec.** | **Sens.** | **MCC** |
| --- | --- | --- | --- | --- | --- | --- | --- |
| **0** | 0.00% | 100.00% | 0.00 | **0.51** | 96.72% | 46.10% | 0.51 |
| **0.01** | 9.61% | 99.72% | 0.12 | **0.52** | 96.82% | 45.38% | 0.51 |
| **0.02** | 22.97% | 98.49% | 0.19 | **0.53** | 96.96% | 44.68% | 0.51 |
| **0.03** | 34.95% | 97.01% | 0.24 | **0.54** | 97.07% | 43.84% | 0.51 |
| **0.04** | 44.47% | 95.48% | 0.29 | **0.55** | 97.24% | 42.81% | 0.50 |
| **0.05** | 52.20% | 93.83% | 0.32 | **0.56** | 97.32% | 42.06% | 0.50 |
| **0.06** | 58.64% | 92.40% | 0.36 | **0.57** | 97.43% | 41.30% | 0.50 |
| **0.07** | 65.30% | 90.48% | 0.39 | **0.58** | 97.58% | 40.32% | 0.49 |
| **0.08** | 69.44% | 89.03% | 0.42 | **0.59** | 97.69% | 39.15% | 0.49 |
| **0.09** | 72.87% | 87.49% | 0.44 | **0.6** | 97.76% | 38.26% | 0.48 |
| **0.1** | 75.75% | 85.95% | 0.46 | **0.61** | 97.86% | 37.36% | 0.48 |
| **0.11** | 78.11% | 84.86% | 0.48 | **0.62** | 97.98% | 36.30% | 0.47 |
| **0.12** | 80.21% | 83.55% | 0.49 | **0.63** | 98.07% | 35.33% | 0.47 |
| **0.13** | 81.86% | 82.44% | 0.50 | **0.64** | 98.16% | 34.18% | 0.46 |
| **0.14** | 83.20% | 81.15% | 0.51 | **0.65** | 98.33% | 32.73% | 0.45 |
| **0.15** | 84.42% | 79.78% | 0.52 | **0.66** | 98.40% | 31.75% | 0.45 |
| **0.16** | 85.44% | 78.58% | 0.53 | **0.67** | 98.48% | 30.44% | 0.44 |
| **0.17** | 86.34% | 77.41% | 0.53 | **0.68** | 98.54% | 29.43% | 0.43 |
| **0.18** | 87.16% | 76.68% | 0.54 | **0.69** | 98.64% | 28.09% | 0.42 |
| **0.19** | 87.82% | 75.31% | 0.54 | **0.7** | 98.70% | 27.12% | 0.42 |
| **0.2** | 88.43% | 74.25% | 0.54 | **0.71** | 98.76% | 25.94% | 0.41 |
| **0.21** | 89.00% | 73.22% | 0.55 | **0.72** | 98.84% | 25.13% | 0.40 |
| **0.22** | 89.56% | 72.27% | 0.55 | **0.73** | 98.96% | 23.93% | 0.39 |
| **0.23** | 90.13% | 71.32% | 0.55 | **0.74** | 99.05% | 22.76% | 0.39 |
| **0.24** | 90.53% | 70.43% | 0.55 | **0.75** | 99.13% | 21.47% | 0.38 |
| **0.25** | 90.92% | 69.76% | 0.56 | **0.76** | 99.24% | 20.36% | 0.37 |
| **0.26** | 91.39% | 68.61% | 0.56 | **0.77** | 99.33% | 19.18% | 0.36 |
| **0.27** | 91.81% | 67.50% | 0.56 | **0.78** | 99.41% | 17.76% | 0.35 |
| **0.28** | 92.10% | 66.85% | 0.56 | **0.79** | 99.45% | 16.28% | 0.33 |
| **0.29** | 92.46% | 66.07% | 0.56 | **0.8** | 99.51% | 15.22% | 0.32 |
| **0.3** | 92.83% | 65.15% | 0.56 | **0.81** | 99.58% | 14.16% | 0.32 |
| **0.31** | 93.11% | 64.09% | 0.56 | **0.82** | 99.62% | 12.85% | 0.30 |
| **0.32** | 93.45% | 63.22% | 0.56 | **0.83** | 99.65% | 11.59% | 0.28 |
| **0.33** | 93.62% | 62.55% | 0.56 | **0.84** | 99.71% | 10.56% | 0.27 |
| **0.34** | 93.87% | 61.71% | 0.56 | **0.85** | 99.74% | 9.63% | 0.26 |
| **0.35** | 94.14% | 60.63% | 0.56 | **0.86** | 99.78% | 8.49% | 0.25 |
| **0.36** | 94.36% | 59.76% | 0.56 | **0.87** | 99.81% | 7.48% | 0.23 |
| **0.37** | 94.56% | 58.73% | 0.55 | **0.88** | 99.84% | 6.42% | 0.21 |
| **0.38** | 94.73% | 58.06% | 0.55 | **0.89** | 99.85% | 5.67% | 0.20 |
| **0.39** | 94.93% | 57.00% | 0.55 | **0.9** | 99.87% | 4.55% | 0.18 |
| **0.4** | 95.10% | 56.35% | 0.55 | **0.91** | 99.92% | 3.60% | 0.16 |
| **0.41** | 95.28% | 55.32% | 0.55 | **0.92** | 99.94% | 2.76% | 0.14 |
| **0.42** | 95.46% | 54.54% | 0.54 | **0.93** | 99.95% | 1.98% | 0.12 |
| **0.43** | 95.60% | 53.76% | 0.54 | **0.94** | 99.97% | 1.54% | 0.11 |
| **0.44** | 95.75% | 52.86% | 0.54 | **0.95** | 99.97% | 1.09% | 0.09 |
| **0.45** | 95.94% | 52.00% | 0.54 | **0.96** | 99.97% | 0.64% | 0.06 |
| **0.46** | 96.12% | 50.91% | 0.53 | **0.97** | 99.98% | 0.39% | 0.05 |
| **0.47** | 96.25% | 49.85% | 0.53 | **0.98** | 99.99% | 0.28% | 0.04 |
| **0.48** | 96.36% | 48.81% | 0.52 | **0.99** | 100.00% | 0.11% | 0.03 |
| **0.49** | 96.48% | 47.81% | 0.52 | **1** | 100.00% | 0.00% | 0.00 |
| **0.5** | 96.66% | 46.58% | 0.52 |  |  |  |  |

1. The experiment result of standard PSSM with –b option in SVM by three-way data split.

| **Threshold** | **Spec.** | **Sens.** | **MCC** | **Threshold** | **Spec.** | **Sens.** | **MCC** |
| --- | --- | --- | --- | --- | --- | --- | --- |
| **0** | 0.00% | 100.00% | 0.00 | **0.51** | 98.10% | 26.81% | 0.38 |
| **0.01** | 2.53% | 99.89% | 0.06 | **0.52** | 98.24% | 25.80% | 0.38 |
| **0.02** | 9.94% | 99.05% | 0.11 | **0.53** | 98.32% | 24.94% | 0.37 |
| **0.03** | 18.61% | 97.18% | 0.15 | **0.54** | 98.41% | 24.24% | 0.37 |
| **0.04** | 27.52% | 95.09% | 0.18 | **0.55** | 98.49% | 23.65% | 0.37 |
| **0.05** | 35.52% | 92.96% | 0.21 | **0.56** | 98.55% | 23.07% | 0.36 |
| **0.06** | 42.99% | 90.92% | 0.24 | **0.57** | 98.63% | 22.26% | 0.36 |
| **0.07** | 50.87% | 88.19% | 0.27 | **0.58** | 98.69% | 21.59% | 0.35 |
| **0.08** | 56.31% | 85.79% | 0.29 | **0.59** | 98.79% | 20.66% | 0.35 |
| **0.09** | 60.97% | 83.13% | 0.31 | **0.6** | 98.83% | 20.11% | 0.34 |
| **0.1** | 65.08% | 80.54% | 0.32 | **0.61** | 98.89% | 19.58% | 0.34 |
| **0.11** | 68.78% | 78.02% | 0.34 | **0.62** | 98.97% | 18.96% | 0.34 |
| **0.12** | 71.88% | 75.87% | 0.35 | **0.63** | 99.02% | 18.35% | 0.33 |
| **0.13** | 74.40% | 73.95% | 0.36 | **0.64** | 99.08% | 17.70% | 0.33 |
| **0.14** | 76.78% | 72.02% | 0.37 | **0.65** | 99.17% | 16.95% | 0.32 |
| **0.15** | 78.70% | 70.06% | 0.38 | **0.66** | 99.24% | 16.48% | 0.32 |
| **0.16** | 80.60% | 68.25% | 0.39 | **0.67** | 99.27% | 15.75% | 0.31 |
| **0.17** | 82.10% | 66.60% | 0.39 | **0.68** | 99.34% | 15.25% | 0.31 |
| **0.18** | 83.56% | 64.73% | 0.40 | **0.69** | 99.37% | 14.69% | 0.31 |
| **0.19** | 84.84% | 63.25% | 0.41 | **0.7** | 99.42% | 14.24% | 0.30 |
| **0.2** | 86.06% | 61.32% | 0.41 | **0.71** | 99.44% | 13.52% | 0.30 |
| **0.21** | 87.09% | 59.73% | 0.41 | **0.72** | 99.48% | 12.96% | 0.29 |
| **0.22** | 88.01% | 58.42% | 0.42 | **0.73** | 99.51% | 12.20% | 0.28 |
| **0.23** | 88.77% | 56.80% | 0.42 | **0.74** | 99.55% | 11.64% | 0.28 |
| **0.24** | 89.59% | 55.38% | 0.42 | **0.75** | 99.59% | 11.11% | 0.27 |
| **0.25** | 90.29% | 53.98% | 0.42 | **0.76** | 99.62% | 10.42% | 0.26 |
| **0.26** | 90.99% | 52.42% | 0.42 | **0.77** | 99.64% | 9.77% | 0.25 |
| **0.27** | 91.59% | 51.33% | 0.43 | **0.78** | 99.66% | 9.49% | 0.25 |
| **0.28** | 92.10% | 49.93% | 0.42 | **0.79** | 99.71% | 8.96% | 0.25 |
| **0.29** | 92.67% | 48.81% | 0.43 | **0.8** | 99.72% | 8.43% | 0.24 |
| **0.3** | 93.08% | 47.78% | 0.43 | **0.81** | 99.76% | 7.93% | 0.23 |
| **0.31** | 93.49% | 46.58% | 0.43 | **0.82** | 99.78% | 7.48% | 0.23 |
| **0.32** | 93.90% | 45.16% | 0.43 | **0.83** | 99.81% | 7.07% | 0.22 |
| **0.33** | 94.11% | 44.48% | 0.42 | **0.84** | 99.85% | 6.42% | 0.22 |
| **0.34** | 94.53% | 43.54% | 0.43 | **0.85** | 99.88% | 6.00% | 0.21 |
| **0.35** | 94.84% | 42.22% | 0.42 | **0.86** | 99.91% | 5.45% | 0.20 |
| **0.36** | 95.22% | 41.30% | 0.43 | **0.87** | 99.92% | 5.03% | 0.20 |
| **0.37** | 95.51% | 40.13% | 0.42 | **0.88** | 99.94% | 4.64% | 0.19 |
| **0.38** | 95.83% | 39.35% | 0.43 | **0.89** | 99.94% | 4.22% | 0.18 |
| **0.39** | 96.03% | 38.23% | 0.42 | **0.9** | 99.95% | 3.71% | 0.17 |
| **0.4** | 96.29% | 37.11% | 0.42 | **0.91** | 99.97% | 3.38% | 0.16 |
| **0.41** | 96.52% | 35.94% | 0.42 | **0.92** | 99.98% | 2.23% | 0.13 |
| **0.42** | 96.72% | 34.99% | 0.41 | **0.93** | 100.00% | 1.56% | 0.11 |
| **0.43** | 96.91% | 34.29% | 0.41 | **0.94** | 100.00% | 1.20% | 0.10 |
| **0.44** | 97.10% | 33.04% | 0.41 | **0.95** | 100.00% | 0.92% | 0.09 |
| **0.45** | 97.26% | 32.23% | 0.41 | **0.96** | 100.00% | 0.73% | 0.08 |
| **0.46** | 97.44% | 31.25% | 0.40 | **0.97** | 100.00% | 0.50% | 0.07 |
| **0.47** | 97.59% | 30.27% | 0.40 | **0.98** | 100.00% | 0.17% | 0.04 |
| **0.48** | 97.75% | 29.29% | 0.39 | **0.99** | 100.00% | 0.03% | 0.02 |
| **0.49** | 97.87% | 28.57% | 0.39 | **1** | 100.00% | 0.00% | 0.00 |
| **0.5** | 98.05% | 27.23% | 0.39 |  |  |  |  |
