# Supplementary material for: Predicting RNA-binding sites of proteins using support vector machines and evolutionary information
Source: BMC Bioinformatics. 2008 Dec 12;9(Suppl 12):S6. doi: 10.1186/1471-2105-9-S12-S6 (PMC2638146; doi:10.1186/1471-2105-9-S12-S6)
Supplement: Additional file 6 — Detailed experimental results on the RBP107 data set. [file 1471-2105-9-S12-S6-S6.doc]

# Experiment results of the RBP107

Table C 1. The detail performance of the RBP107 with different sliding window size under (A) five-fold cross-validation (w1 = 7.63, w-1 = 1, other parameters: default value) and three-way data split (w1 = 7.63, w-1 = 1, other parameters: default value).

1. Five-fold cross-validation.

| **Window Size** | **Spec.** | **Sens.** | **MCC** | **Acc** |
| --- | --- | --- | --- | --- |
| **3** | 75.28% | 70.57% | 0.32 | 74.73% |
| **5** | 76.77% | 70.96% | 0.34 | 76.10% |
| **7** | 78.08% | 71.90% | 0.36 | 77.37% |
| **9** | 78.58% | 72.41% | 0.37 | 77.86% |
| **11** | 79.42% | 71.51% | 0.37 | 78.50% |
| **13** | 79.65% | 71.39% | 0.37 | 78.69% |
| **15** | 80.09% | 71.51% | 0.38 | 79.10% |
| **17** | 80.17% | 71.35% | 0.38 | 79.15% |
| **19** | 80.43% | 71.12% | 0.38 | 79.35% |
| **21** | 80.60% | 70.96% | 0.38 | 79.48% |
| **23** | 80.95% | 71.55% | 0.39 | 79.86% |
| **25** | 80.83% | 71.19% | 0.38 | 79.72% |
| **27** | 80.80% | 71.12% | 0.38 | 79.68% |
| **29** | 80.87% | 71.08% | 0.38 | 79.74% |
| **31** | 80.95% | 71.39% | 0.39 | 79.84% |
| **33** | 81.05% | 71.00% | 0.38 | 79.89% |
| **35** | 81.08% | 71.00% | 0.39 | 79.91% |
| **37** | 81.07% | 70.68% | 0.38 | 79.86% |
| **39** | 80.99% | 71.04% | 0.38 | 79.84% |
| **41** | 81.07% | 69.98% | 0.38 | 79.78% |

1. Three-way data split.

| **Window Size** | **Spec.** | **Sens.** | **MCC** | **Acc** |
| --- | --- | --- | --- | --- |
| **3** | 75.18% | 69.98% | 0.32 | 74.58% |
| **5** | 76.75% | 70.65% | 0.34 | 76.05% |
| **7** | 78.18% | 70.96% | 0.35 | 77.34% |
| **9** | 78.53% | 71.62% | 0.36 | 77.73% |
| **11** | 79.32% | 71.23% | 0.37 | 78.38% |
| **13** | 79.49% | 71.12% | 0.37 | 78.52% |
| **15** | 79.93% | 71.12% | 0.37 | 78.91% |
| **17** | 80.14% | 70.88% | 0.37 | 79.07% |
| **19** | 80.46% | 70.57% | 0.37 | 79.31% |
| **21** | 80.34% | 70.22% | 0.37 | 79.17% |
| **23** | 80.96% | 70.06% | 0.38 | 79.70% |
| **25** | 81.04% | 70.53% | 0.38 | 79.82% |
| **27** | 81.24% | 70.49% | 0.38 | 79.99% |
| **29** | 81.13% | 69.94% | 0.38 | 79.83% |
| **31** | 81.18% | 69.90% | 0.38 | 79.87% |
| **33** | 81.21% | 69.90% | 0.38 | 79.90% |
| **35** | 81.27% | 69.47% | 0.38 | 79.90% |
| **37** | 81.27% | 69.43% | 0.38 | 79.90% |
| **39** | 81.37% | 69.28% | 0.38 | 79.96% |
| **41** | 81.36% | 69.47% | 0.38 | 79.98% |

1. Five-fold cross-validation.


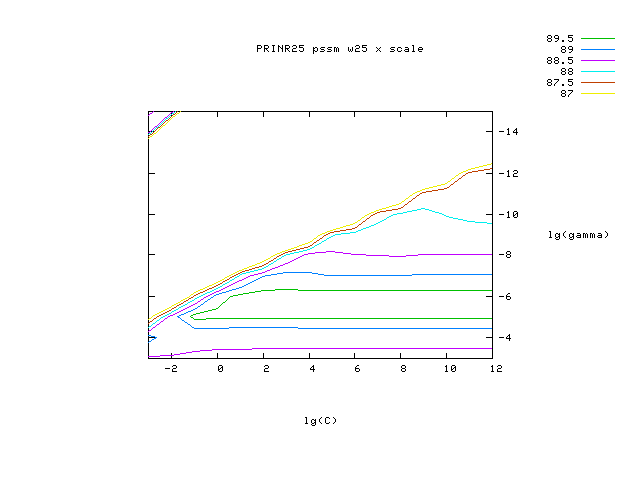


1. Three-way data split.


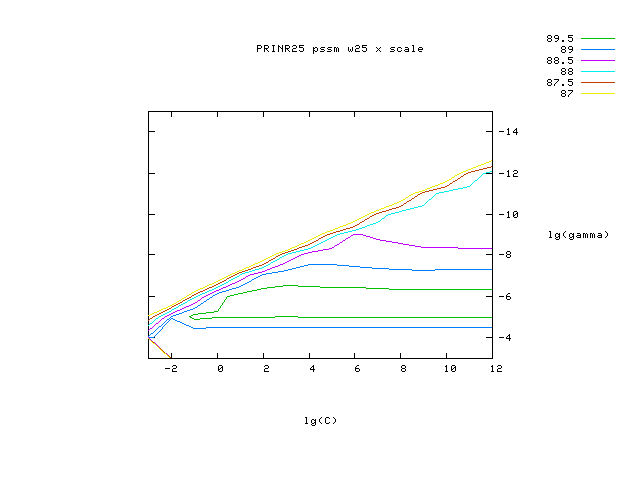


Figure C 1. The performance with different combination of C and γ in the RBP107 data set under (A) five-fold cross-validation and (B) three-way data split.

Table C 2. The detail performance of the RBP107 with (A) different smoothing window size under five-fold cross-validation (w = 25, log C = 2, log γ = -6, w1 = 7.63, w-1 = 1, other parameters: default value) and (B) different smoothing window size under three-way data split (w = 25, log C = 3, log γ = -6, w1 = 7.63, w-1 = 1, other parameters: default value).

1. Five-fold cross-validation.

| **Smoothing Window Size** | **Spec.** | **Sens.** | **MCC** | **Acc** |
| --- | --- | --- | --- | --- |
| **1** | 97.80% | 27.83% | 0.37 | 89.69% |
| **3** | 94.91% | 45.21% | 0.43 | 89.15% |
| **5** | 93.09% | 54.64% | 0.46 | 88.63% |
| **7** | 91.44% | 59.65% | 0.46 | 87.76% |
| **9** | 90.22% | 63.29% | 0.47 | 87.10% |
| **11** | 88.85% | 65.83% | 0.46 | 86.18% |

1. Three-way data split.

| **Smoothing Window Size** | **Spec.** | **Sens.** | **MCC** | **Acc** |
| --- | --- | --- | --- | --- |
| **1** | 98.04% | 26.18% | 0.36 | 89.71% |
| **3** | 95.91% | 38.20% | 0.40 | 89.23% |
| **5** | 94.64% | 45.95% | 0.43 | 89.00% |
| **7** | 93.51% | 49.98% | 0.44 | 88.47% |
| **9** | 92.68% | 53.86% | 0.45 | 88.18% |
| **11** | 91.60% | 57.10% | 0.45 | 87.61% |

Table C 3. The detail performance of the RBP107 with (A) different weight parameter w1 under five-fold cross-validation (w = 25, log C = 2, log γ = -6, ws = 7, w-1 = 1, other parameters: default value) and (B) different weight parameter w1 under three-way data split (w = 25, log C = 3, log γ = -6, ws = 7, w-1 = 1, other parameters: default value).

1. Five-fold cross-validation.

| **W1** | **Spec.** | **Sens.** | **MCC** | **Acc** |
| --- | --- | --- | --- | --- |
| **1** | 97.81% | 29.75% | 0.39 | 89.93% |
| **2** | 94.86% | 48.77% | 0.46 | 89.52% |
| **3** | 93.27% | 55.54% | 0.47 | 88.90% |
| **4** | 92.29% | 58.47% | 0.47 | 88.37% |
| **5** | 91.79% | 58.87% | 0.47 | 87.98% |
| **6** | 91.59% | 59.33% | 0.47 | 87.86% |
| **7** | 91.48% | 59.49% | 0.46 | 87.77% |
| **8** | 91.44% | 59.69% | 0.47 | 87.76% |
| **9** | 91.43% | 59.69% | 0.46 | 87.75% |
| **10** | 91.42% | 59.73% | 0.46 | 87.75% |

1. Three-way data split.

| **W1** | **Spec.** | **Sens.** | **MCC** | **Acc** |
| --- | --- | --- | --- | --- |
| **1** | 96.54% | 36.75% | 0.41 | 89.62% |
| **2** | 94.28% | 47.40% | 0.43 | 88.85% |
| **3** | 93.79% | 49.39% | 0.44 | 88.64% |
| **4** | 93.59% | 49.86% | 0.44 | 88.53% |
| **5** | 93.53% | 50.06% | 0.44 | 88.49% |
| **6** | 93.51% | 49.98% | 0.44 | 88.46% |
| **7** | 93.51% | 49.98% | 0.44 | 88.47% |
| **8** | 93.51% | 49.98% | 0.44 | 88.47% |
| **9** | 93.51% | 49.98% | 0.44 | 88.47% |
| **10** | 93.51% | 49.98% | 0.44 | 88.47% |

Table C 4. The RBP107 data set experiment results with –b option in SVM for (A) smoothed PSSM by five-fold cross-validation, (B) standard PSSM by five-fold cross-validation, (C) smoothed PSSM by three-way data split, and (D) standard PSSM by three-way data split.

1. **The experiment result of smoothed PSSM with –b option in SVM by five-fold cross-validation.**

| **Threshold** | **Spec.** | **Sens.** | **MCC** | **Threshold** | **Spec.** | **Sens.** | **MCC** |
| --- | --- | --- | --- | --- | --- | --- | --- |
| **0** | 0.00% | 100.00% | 0.00 | **0.51** | 97.47% | 32.72% | 0.41 |
| **0.01** | 7.60% | 99.61% | 0.09 | **0.52** | 97.60% | 31.86% | 0.40 |
| **0.02** | 21.19% | 98.24% | 0.16 | **0.53** | 97.72% | 30.22% | 0.39 |
| **0.03** | 34.93% | 96.09% | 0.21 | **0.54** | 97.80% | 28.65% | 0.38 |
| **0.04** | 46.04% | 93.31% | 0.26 | **0.55** | 97.90% | 27.75% | 0.37 |
| **0.05** | 54.70% | 90.53% | 0.29 | **0.56** | 98.02% | 26.81% | 0.37 |
| **0.06** | 61.88% | 88.49% | 0.32 | **0.57** | 98.14% | 25.60% | 0.36 |
| **0.07** | 68.56% | 85.68% | 0.36 | **0.58** | 98.26% | 24.31% | 0.35 |
| **0.08** | 72.79% | 83.37% | 0.38 | **0.59** | 98.35% | 23.68% | 0.35 |
| **0.09** | 76.38% | 81.10% | 0.40 | **0.6** | 98.41% | 22.54% | 0.34 |
| **0.1** | 78.88% | 79.14% | 0.41 | **0.61** | 98.53% | 21.33% | 0.33 |
| **0.11** | 80.87% | 77.14% | 0.42 | **0.62** | 98.60% | 20.27% | 0.32 |
| **0.12** | 82.65% | 74.95% | 0.43 | **0.63** | 98.70% | 19.37% | 0.32 |
| **0.13** | 83.94% | 73.58% | 0.44 | **0.64** | 98.79% | 18.24% | 0.31 |
| **0.14** | 85.10% | 72.56% | 0.45 | **0.65** | 98.90% | 17.38% | 0.31 |
| **0.15** | 86.07% | 71.23% | 0.45 | **0.66** | 98.97% | 16.40% | 0.30 |
| **0.16** | 86.93% | 69.43% | 0.45 | **0.67** | 99.04% | 15.50% | 0.29 |
| **0.17** | 87.71% | 67.83% | 0.46 | **0.68** | 99.12% | 14.40% | 0.28 |
| **0.18** | 88.37% | 66.54% | 0.46 | **0.69** | 99.22% | 13.50% | 0.27 |
| **0.19** | 89.06% | 65.44% | 0.46 | **0.7** | 99.31% | 12.76% | 0.27 |
| **0.2** | 89.66% | 64.66% | 0.47 | **0.71** | 99.37% | 12.09% | 0.27 |
| **0.21** | 90.06% | 63.52% | 0.47 | **0.72** | 99.44% | 10.80% | 0.25 |
| **0.22** | 90.52% | 62.50% | 0.47 | **0.73** | 99.50% | 9.90% | 0.24 |
| **0.23** | 90.94% | 61.33% | 0.47 | **0.74** | 99.57% | 8.88% | 0.23 |
| **0.24** | 91.36% | 60.31% | 0.47 | **0.75** | 99.60% | 8.14% | 0.22 |
| **0.25** | 91.72% | 59.84% | 0.47 | **0.76** | 99.64% | 7.08% | 0.20 |
| **0.26** | 92.13% | 58.83% | 0.47 | **0.77** | 99.69% | 6.26% | 0.19 |
| **0.27** | 92.50% | 57.69% | 0.47 | **0.78** | 99.73% | 5.28% | 0.17 |
| **0.28** | 92.81% | 56.44% | 0.47 | **0.79** | 99.74% | 4.85% | 0.17 |
| **0.29** | 93.12% | 55.23% | 0.47 | **0.8** | 99.78% | 4.27% | 0.16 |
| **0.3** | 93.43% | 53.93% | 0.47 | **0.81** | 99.80% | 3.84% | 0.15 |
| **0.31** | 93.77% | 52.96% | 0.47 | **0.82** | 99.85% | 3.21% | 0.14 |
| **0.32** | 94.05% | 51.86% | 0.46 | **0.83** | 99.87% | 2.97% | 0.13 |
| **0.33** | 94.21% | 51.15% | 0.46 | **0.84** | 99.88% | 2.58% | 0.12 |
| **0.34** | 94.49% | 50.22% | 0.46 | **0.85** | 99.90% | 2.19% | 0.12 |
| **0.35** | 94.68% | 49.28% | 0.46 | **0.86** | 99.92% | 1.76% | 0.10 |
| **0.36** | 94.90% | 48.45% | 0.46 | **0.87** | 99.94% | 1.45% | 0.09 |
| **0.37** | 95.16% | 47.16% | 0.46 | **0.88** | 99.94% | 1.33% | 0.09 |
| **0.38** | 95.36% | 45.95% | 0.45 | **0.89** | 99.96% | 0.98% | 0.08 |
| **0.39** | 95.55% | 44.97% | 0.45 | **0.9** | 99.97% | 0.74% | 0.07 |
| **0.4** | 95.73% | 43.68% | 0.44 | **0.91** | 99.97% | 0.43% | 0.05 |
| **0.41** | 95.92% | 42.86% | 0.44 | **0.92** | 99.98% | 0.31% | 0.04 |
| **0.42** | 96.11% | 42.00% | 0.44 | **0.93** | 99.98% | 0.27% | 0.04 |
| **0.43** | 96.30% | 40.94% | 0.44 | **0.94** | 100.00% | 0.20% | 0.04 |
| **0.44** | 96.41% | 40.00% | 0.43 | **0.95** | 100.00% | 0.04% | 0.02 |
| **0.45** | 96.59% | 38.71% | 0.43 | **0.96** | 100.00% | 0.00% | 0.00 |
| **0.46** | 96.74% | 37.57% | 0.42 | **0.97** | 100.00% | 0.00% | 0.00 |
| **0.47** | 96.90% | 36.83% | 0.42 | **0.98** | 100.00% | 0.00% | 0.00 |
| **0.48** | 97.06% | 35.73% | 0.42 | **0.99** | 100.00% | 0.00% | 0.00 |
| **0.49** | 97.24% | 34.32% | 0.41 | **1** | 100.00% | 0.00% | 0.00 |
| **0.5** | 97.45% | 33.15% | 0.41 |  |  |  |  |

1. **The experiment result of standard PSSM with –b option in SVM by five-fold cross-validation.**

| **Threshold** | **Spec.** | **Sens.** | **MCC** | **Threshold** | **Spec.** | **Sens.** | **MCC** |
| --- | --- | --- | --- | --- | --- | --- | --- |
| **0** | 0.00% | 100.00% | 0.00 | **0.51** | 98.52% | 21.84% | 0.34 |
| **0.01** | 3.03% | 99.61% | 0.05 | **0.52** | 98.56% | 21.17% | 0.33 |
| **0.02** | 12.75% | 98.63% | 0.11 | **0.53** | 98.60% | 20.67% | 0.33 |
| **0.03** | 23.83% | 96.48% | 0.16 | **0.54** | 98.66% | 20.00% | 0.32 |
| **0.04** | 34.14% | 93.66% | 0.19 | **0.55** | 98.71% | 19.49% | 0.32 |
| **0.05** | 43.04% | 90.57% | 0.22 | **0.56** | 98.74% | 18.83% | 0.31 |
| **0.06** | 51.69% | 87.63% | 0.25 | **0.57** | 98.79% | 18.20% | 0.31 |
| **0.07** | 59.83% | 83.87% | 0.28 | **0.58** | 98.86% | 17.57% | 0.31 |
| **0.08** | 64.93% | 80.82% | 0.30 | **0.59** | 98.92% | 16.83% | 0.30 |
| **0.09** | 69.40% | 77.65% | 0.31 | **0.6** | 98.97% | 16.01% | 0.29 |
| **0.1** | 73.06% | 75.19% | 0.33 | **0.61** | 99.03% | 15.69% | 0.29 |
| **0.11** | 76.10% | 73.03% | 0.34 | **0.62** | 99.07% | 14.95% | 0.28 |
| **0.12** | 78.74% | 70.61% | 0.36 | **0.63** | 99.11% | 14.44% | 0.28 |
| **0.13** | 80.82% | 68.53% | 0.37 | **0.64** | 99.15% | 13.86% | 0.27 |
| **0.14** | 82.69% | 66.18% | 0.37 | **0.65** | 99.22% | 13.11% | 0.27 |
| **0.15** | 84.29% | 63.84% | 0.38 | **0.66** | 99.26% | 12.29% | 0.26 |
| **0.16** | 85.71% | 61.80% | 0.38 | **0.67** | 99.28% | 11.59% | 0.25 |
| **0.17** | 86.95% | 59.69% | 0.38 | **0.68** | 99.33% | 11.08% | 0.25 |
| **0.18** | 88.16% | 57.85% | 0.39 | **0.69** | 99.36% | 10.88% | 0.24 |
| **0.19** | 89.22% | 55.93% | 0.39 | **0.7** | 99.39% | 10.06% | 0.23 |
| **0.2** | 90.06% | 54.21% | 0.40 | **0.71** | 99.41% | 9.43% | 0.22 |
| **0.21** | 90.86% | 52.80% | 0.40 | **0.72** | 99.45% | 8.73% | 0.22 |
| **0.22** | 91.66% | 51.74% | 0.41 | **0.73** | 99.47% | 8.34% | 0.21 |
| **0.23** | 92.27% | 49.67% | 0.40 | **0.74** | 99.48% | 7.98% | 0.20 |
| **0.24** | 92.85% | 48.10% | 0.40 | **0.75** | 99.50% | 7.36% | 0.19 |
| **0.25** | 93.38% | 46.54% | 0.40 | **0.76** | 99.54% | 7.08% | 0.19 |
| **0.26** | 93.81% | 45.05% | 0.40 | **0.77** | 99.56% | 6.65% | 0.19 |
| **0.27** | 94.17% | 43.87% | 0.40 | **0.78** | 99.60% | 6.34% | 0.18 |
| **0.28** | 94.53% | 42.54% | 0.40 | **0.79** | 99.63% | 5.99% | 0.18 |
| **0.29** | 94.84% | 41.68% | 0.40 | **0.8** | 99.65% | 5.48% | 0.17 |
| **0.3** | 95.17% | 40.39% | 0.40 | **0.81** | 99.67% | 4.85% | 0.16 |
| **0.31** | 95.45% | 38.75% | 0.39 | **0.82** | 99.69% | 4.62% | 0.15 |
| **0.32** | 95.72% | 37.73% | 0.39 | **0.83** | 99.72% | 4.15% | 0.15 |
| **0.33** | 95.89% | 37.14% | 0.39 | **0.84** | 99.76% | 3.76% | 0.14 |
| **0.34** | 96.09% | 35.73% | 0.38 | **0.85** | 99.78% | 3.37% | 0.13 |
| **0.35** | 96.38% | 34.76% | 0.38 | **0.86** | 99.82% | 3.05% | 0.13 |
| **0.36** | 96.63% | 33.86% | 0.38 | **0.87** | 99.86% | 2.47% | 0.12 |
| **0.37** | 96.86% | 32.84% | 0.38 | **0.88** | 99.89% | 2.11% | 0.11 |
| **0.38** | 97.07% | 32.05% | 0.38 | **0.89** | 99.92% | 1.80% | 0.10 |
| **0.39** | 97.26% | 30.61% | 0.38 | **0.9** | 99.94% | 1.29% | 0.09 |
| **0.4** | 97.43% | 29.98% | 0.38 | **0.91** | 99.97% | 0.90% | 0.08 |
| **0.41** | 97.54% | 29.47% | 0.38 | **0.92** | 99.98% | 0.74% | 0.07 |
| **0.42** | 97.66% | 28.57% | 0.37 | **0.93** | 99.99% | 0.63% | 0.07 |
| **0.43** | 97.76% | 27.87% | 0.37 | **0.94** | 99.99% | 0.51% | 0.06 |
| **0.44** | 97.91% | 26.97% | 0.37 | **0.95** | 100.00% | 0.27% | 0.05 |
| **0.45** | 98.03% | 26.18% | 0.36 | **0.96** | 100.00% | 0.16% | 0.04 |
| **0.46** | 98.12% | 25.44% | 0.36 | **0.97** | 100.00% | 0.04% | 0.02 |
| **0.47** | 98.19% | 24.62% | 0.35 | **0.98** | 100.00% | 0.00% | 0.00 |
| **0.48** | 98.28% | 24.03% | 0.35 | **0.99** | 100.00% | 0.00% | 0.00 |
| **0.49** | 98.37% | 23.17% | 0.35 | **1** | 100.00% | 0.00% | 0.00 |
| **0.5** | 98.48% | 22.07% | 0.34 |  |  |  |  |

1. The experiment result of smoothed PSSM with –b option in SVM by three-way data split.

| **Threshold** | **Spec.** | **Sens.** | **MCC** | **Threshold** | **Spec.** | **Sens.** | **MCC** |
| --- | --- | --- | --- | --- | --- | --- | --- |
| **0** | 0.00% | 100.00% | 0.00 | **0.51** | 97.78% | 27.87% | 0.37 |
| **0.01** | 5.11% | 99.80% | 0.08 | **0.52** | 97.90% | 27.24% | 0.37 |
| **0.02** | 17.04% | 98.55% | 0.14 | **0.53** | 98.00% | 26.42% | 0.36 |
| **0.03** | 29.12% | 96.48% | 0.19 | **0.54** | 98.11% | 25.60% | 0.36 |
| **0.04** | 40.02% | 94.64% | 0.23 | **0.55** | 98.18% | 24.58% | 0.35 |
| **0.05** | 49.04% | 91.90% | 0.26 | **0.56** | 98.32% | 23.25% | 0.34 |
| **0.06** | 56.72% | 89.24% | 0.29 | **0.57** | 98.39% | 22.15% | 0.34 |
| **0.07** | 64.12% | 85.87% | 0.32 | **0.58** | 98.47% | 21.14% | 0.33 |
| **0.08** | 69.08% | 83.56% | 0.35 | **0.59** | 98.57% | 20.16% | 0.32 |
| **0.09** | 72.94% | 80.86% | 0.37 | **0.6** | 98.66% | 19.10% | 0.31 |
| **0.1** | 76.05% | 77.57% | 0.37 | **0.61** | 98.73% | 18.08% | 0.31 |
| **0.11** | 78.68% | 75.58% | 0.39 | **0.62** | 98.80% | 17.26% | 0.30 |
| **0.12** | 80.65% | 73.62% | 0.40 | **0.63** | 98.90% | 16.75% | 0.30 |
| **0.13** | 82.53% | 71.51% | 0.41 | **0.64** | 98.96% | 15.85% | 0.29 |
| **0.14** | 83.96% | 69.32% | 0.41 | **0.65** | 99.09% | 14.60% | 0.28 |
| **0.15** | 85.19% | 67.71% | 0.42 | **0.66** | 99.17% | 13.74% | 0.27 |
| **0.16** | 86.25% | 66.22% | 0.42 | **0.67** | 99.24% | 12.92% | 0.27 |
| **0.17** | 87.24% | 64.85% | 0.43 | **0.68** | 99.29% | 12.09% | 0.26 |
| **0.18** | 88.05% | 62.97% | 0.43 | **0.69** | 99.32% | 11.78% | 0.26 |
| **0.19** | 88.82% | 61.33% | 0.43 | **0.7** | 99.37% | 11.08% | 0.25 |
| **0.2** | 89.52% | 60.27% | 0.43 | **0.71** | 99.44% | 10.06% | 0.24 |
| **0.21** | 90.20% | 59.14% | 0.44 | **0.72** | 99.48% | 9.35% | 0.23 |
| **0.22** | 90.74% | 57.65% | 0.44 | **0.73** | 99.54% | 8.85% | 0.23 |
| **0.23** | 91.31% | 56.28% | 0.44 | **0.74** | 99.61% | 8.49% | 0.23 |
| **0.24** | 91.72% | 55.11% | 0.44 | **0.75** | 99.68% | 7.55% | 0.22 |
| **0.25** | 92.13% | 54.05% | 0.44 | **0.76** | 99.69% | 7.05% | 0.21 |
| **0.26** | 92.53% | 52.96% | 0.44 | **0.77** | 99.71% | 6.22% | 0.19 |
| **0.27** | 92.91% | 51.90% | 0.44 | **0.78** | 99.75% | 5.83% | 0.19 |
| **0.28** | 93.26% | 50.88% | 0.44 | **0.79** | 99.78% | 5.01% | 0.18 |
| **0.29** | 93.61% | 50.02% | 0.44 | **0.8** | 99.84% | 4.23% | 0.16 |
| **0.3** | 93.89% | 48.81% | 0.44 | **0.81** | 99.87% | 3.84% | 0.16 |
| **0.31** | 94.16% | 47.83% | 0.43 | **0.82** | 99.90% | 3.44% | 0.15 |
| **0.32** | 94.42% | 46.89% | 0.43 | **0.83** | 99.92% | 2.90% | 0.14 |
| **0.33** | 94.59% | 46.18% | 0.43 | **0.84** | 99.93% | 2.39% | 0.13 |
| **0.34** | 94.83% | 44.78% | 0.43 | **0.85** | 99.93% | 1.80% | 0.11 |
| **0.35** | 95.08% | 43.84% | 0.43 | **0.86** | 99.95% | 1.49% | 0.10 |
| **0.36** | 95.30% | 42.54% | 0.42 | **0.87** | 99.95% | 1.25% | 0.09 |
| **0.37** | 95.54% | 41.49% | 0.42 | **0.88** | 99.96% | 1.21% | 0.09 |
| **0.38** | 95.73% | 40.43% | 0.42 | **0.89** | 99.97% | 0.82% | 0.07 |
| **0.39** | 95.91% | 39.22% | 0.41 | **0.9** | 99.97% | 0.59% | 0.06 |
| **0.4** | 96.10% | 37.89% | 0.40 | **0.91** | 99.97% | 0.35% | 0.04 |
| **0.41** | 96.30% | 37.18% | 0.40 | **0.92** | 99.98% | 0.31% | 0.04 |
| **0.42** | 96.47% | 36.20% | 0.40 | **0.93** | 99.98% | 0.23% | 0.03 |
| **0.43** | 96.61% | 35.23% | 0.40 | **0.94** | 99.99% | 0.20% | 0.03 |
| **0.44** | 96.77% | 34.21% | 0.39 | **0.95** | 99.99% | 0.08% | 0.02 |
| **0.45** | 96.88% | 33.19% | 0.39 | **0.96** | 99.99% | 0.04% | 0.01 |
| **0.46** | 97.09% | 32.52% | 0.39 | **0.97** | 100.00% | 0.04% | 0.02 |
| **0.47** | 97.22% | 31.62% | 0.39 | **0.98** | 100.00% | 0.00% | 0.00 |
| **0.48** | 97.39% | 30.57% | 0.38 | **0.99** | 100.00% | 0.00% | 0.00 |
| **0.49** | 97.52% | 29.47% | 0.38 | **1** | 100.00% | 0.00% | 0.00 |
| **0.5** | 97.74% | 28.30% | 0.37 |  |  |  |  |

1. The experiment result of standard PSSM with –b option in SVM by three-way data split.

| **Threshold** | **Spec.** | **Sens.** | **MCC** | **Threshold** | **Spec.** | **Sens.** | **MCC** |
| --- | --- | --- | --- | --- | --- | --- | --- |
| **0** | 0.00% | 100.00% | 0.00 | **0.51** | 98.66% | 20.08% | 0.33 |
| **0.01** | 3.04% | 99.73% | 0.05 | **0.52** | 98.73% | 19.33% | 0.32 |
| **0.02** | 12.44% | 98.79% | 0.11 | **0.53** | 98.79% | 18.55% | 0.31 |
| **0.03** | 23.07% | 96.67% | 0.16 | **0.54** | 98.85% | 17.77% | 0.31 |
| **0.04** | 33.19% | 94.52% | 0.19 | **0.55** | 98.92% | 17.26% | 0.31 |
| **0.05** | 42.39% | 91.66% | 0.22 | **0.56** | 98.94% | 16.71% | 0.30 |
| **0.06** | 50.56% | 88.22% | 0.25 | **0.57** | 98.98% | 16.20% | 0.30 |
| **0.07** | 58.88% | 84.74% | 0.28 | **0.58** | 99.02% | 15.62% | 0.29 |
| **0.08** | 64.43% | 81.53% | 0.30 | **0.59** | 99.08% | 15.19% | 0.29 |
| **0.09** | 68.83% | 78.75% | 0.32 | **0.6** | 99.14% | 14.68% | 0.29 |
| **0.1** | 72.64% | 76.16% | 0.33 | **0.61** | 99.21% | 14.09% | 0.28 |
| **0.11** | 75.86% | 73.11% | 0.34 | **0.62** | 99.23% | 13.54% | 0.28 |
| **0.12** | 78.46% | 70.57% | 0.35 | **0.63** | 99.25% | 13.27% | 0.27 |
| **0.13** | 80.62% | 67.83% | 0.36 | **0.64** | 99.28% | 12.84% | 0.27 |
| **0.14** | 82.56% | 65.83% | 0.37 | **0.65** | 99.33% | 12.25% | 0.26 |
| **0.15** | 84.25% | 63.68% | 0.37 | **0.66** | 99.37% | 11.74% | 0.26 |
| **0.16** | 85.67% | 61.25% | 0.38 | **0.67** | 99.40% | 11.55% | 0.26 |
| **0.17** | 86.86% | 58.98% | 0.38 | **0.68** | 99.42% | 11.23% | 0.26 |
| **0.18** | 88.03% | 57.30% | 0.38 | **0.69** | 99.45% | 10.88% | 0.25 |
| **0.19** | 89.02% | 55.07% | 0.38 | **0.7** | 99.46% | 10.45% | 0.25 |
| **0.2** | 89.89% | 53.31% | 0.39 | **0.71** | 99.50% | 9.82% | 0.24 |
| **0.21** | 90.59% | 51.90% | 0.39 | **0.72** | 99.53% | 9.32% | 0.23 |
| **0.22** | 91.28% | 50.72% | 0.39 | **0.73** | 99.57% | 8.85% | 0.23 |
| **0.23** | 91.95% | 49.24% | 0.39 | **0.74** | 99.59% | 8.26% | 0.22 |
| **0.24** | 92.53% | 47.48% | 0.39 | **0.75** | 99.59% | 8.06% | 0.22 |
| **0.25** | 93.10% | 45.91% | 0.39 | **0.76** | 99.62% | 7.67% | 0.21 |
| **0.26** | 93.62% | 44.58% | 0.39 | **0.77** | 99.65% | 6.97% | 0.20 |
| **0.27** | 94.10% | 43.17% | 0.39 | **0.78** | 99.67% | 6.54% | 0.19 |
| **0.28** | 94.56% | 41.76% | 0.39 | **0.79** | 99.69% | 6.14% | 0.19 |
| **0.29** | 94.96% | 40.20% | 0.39 | **0.8** | 99.70% | 5.64% | 0.18 |
| **0.3** | 95.36% | 38.83% | 0.39 | **0.81** | 99.74% | 5.24% | 0.17 |
| **0.31** | 95.66% | 37.50% | 0.39 | **0.82** | 99.75% | 4.85% | 0.17 |
| **0.32** | 95.92% | 36.59% | 0.39 | **0.83** | 99.77% | 4.54% | 0.16 |
| **0.33** | 96.07% | 35.89% | 0.39 | **0.84** | 99.78% | 3.99% | 0.15 |
| **0.34** | 96.30% | 34.56% | 0.38 | **0.85** | 99.80% | 3.64% | 0.14 |
| **0.35** | 96.53% | 33.58% | 0.38 | **0.86** | 99.83% | 3.41% | 0.14 |
| **0.36** | 96.72% | 32.52% | 0.38 | **0.87** | 99.84% | 3.09% | 0.13 |
| **0.37** | 96.91% | 31.43% | 0.37 | **0.88** | 99.87% | 2.62% | 0.12 |
| **0.38** | 97.10% | 30.53% | 0.37 | **0.89** | 99.89% | 2.00% | 0.11 |
| **0.39** | 97.26% | 29.55% | 0.37 | **0.9** | 99.93% | 1.53% | 0.10 |
| **0.4** | 97.49% | 28.57% | 0.36 | **0.91** | 99.94% | 0.98% | 0.07 |
| **0.41** | 97.56% | 27.91% | 0.36 | **0.92** | 99.96% | 0.70% | 0.06 |
| **0.42** | 97.70% | 26.97% | 0.36 | **0.93** | 99.99% | 0.47% | 0.06 |
| **0.43** | 97.83% | 26.26% | 0.35 | **0.94** | 99.99% | 0.35% | 0.05 |
| **0.44** | 97.93% | 25.28% | 0.35 | **0.95** | 99.99% | 0.12% | 0.03 |
| **0.45** | 98.03% | 24.27% | 0.34 | **0.96** | 100.00% | 0.08% | 0.03 |
| **0.46** | 98.15% | 23.56% | 0.34 | **0.97** | 100.00% | 0.00% | 0.00 |
| **0.47** | 98.26% | 22.66% | 0.34 | **0.98** | 100.00% | 0.00% | 0.00 |
| **0.48** | 98.36% | 21.76% | 0.33 | **0.99** | 100.00% | 0.00% | 0.00 |
| **0.49** | 98.47% | 21.21% | 0.33 | **1** | 100.00% | 0.00% | 0.00 |
| **0.5** | 98.62% | 20.39% | 0.33 |  |  |  |  |
